# Design, step-economical diversity-oriented synthesis of an N-heterocyclic library containing a pyrimidine moiety: discovery of novel potential herbicidal agents†

**DOI:** 10.1039/d1ra02663a

**Published:** 2021-04-26

**Authors:** Dong Ma, Yang Yin, Ying-Lu Chen, Yi-Tao Yan, Jun Wu

**Affiliations:** Department of Chemistry, Zhejiang University Hangzhou 310027 P. R. China wujunwu@zju.edu.cn +86-571-87951895

## Abstract

The synthesis of highly diverse libraries has become of paramount importance for obtaining novel leads for drug and agrochemical discovery. Herein, the step-economical diversity-oriented synthesis of a library of various pyrimidine–N-heterocycle hybrids was developed, in which a 4,6-dimethoxypyrimidine core was incorporated into nine kinds of N-heterocycles. A total of 34 structurally diverse compounds were synthesized *via* a two-step process from very simple and commercially available starting materials. Further, *in vivo* biological screening of this library identified 11 active compounds that exhibited good post-emergence herbicidal activity against *D*. *sanguinalis* at 750 g ai per ha. More importantly, pyrimidine–tetrahydrocarbazole hybrid 5q showed good to excellent herbicidal activity against five test weeds at the same dosage. Pyrimidine–tetrahydrocarbazole hybrids represent a novel class of herbicidal agents that may become promising lead compounds in the herbicidal discovery process.

## Abstract 2

A diverse library of pyrimidine–N-heterocycle hybrids was developed through a step-economical diversity-oriented synthesis strategy. *In vivo* biological screening showed some derivatives exhibited significant potential herbicidal activity.

## Graphical abstract

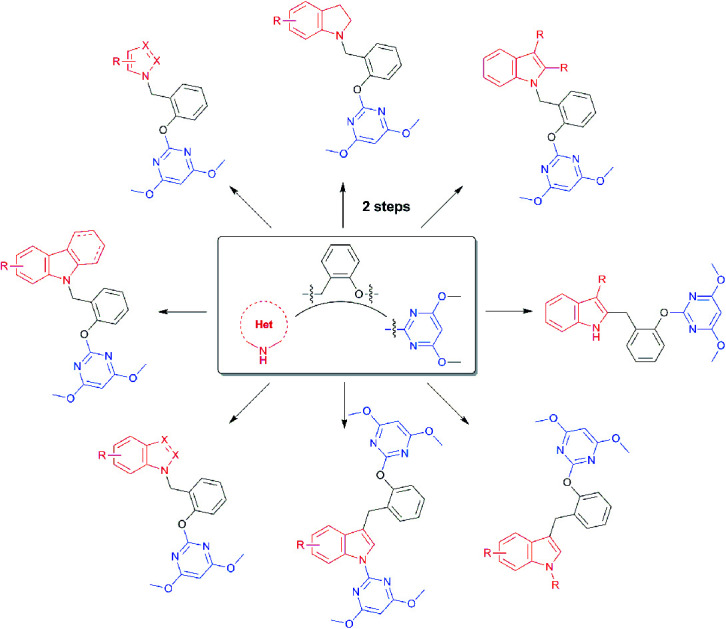

## Introduction

**Fig. 1:**
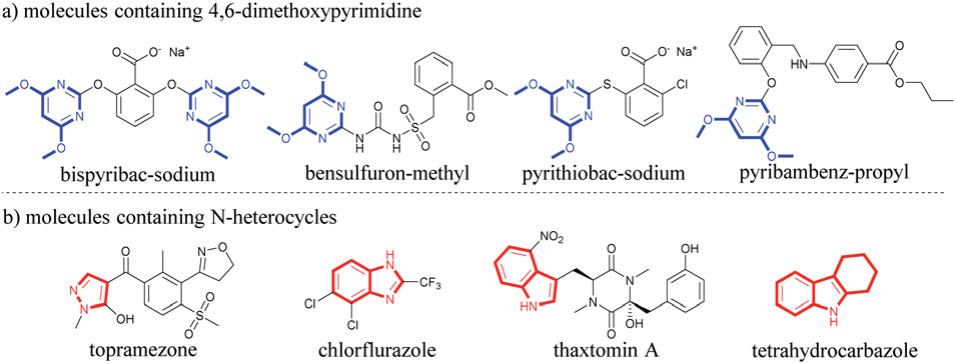
Biological molecules containing 4,6-dimethoxypyrimidine and N-heterocycle scaffolds.

Pyrimidine as a core structure widely exists in natural products.^1^ It has shown prominent pharmaceutical and agricultural activity,^2^ and acts as anti-cancer,^3^ anti-HIV,^4^ and anti-microbial^5^ agents, insecticides,^6^ fungicides^7^ and herbicides.^8^ 4,6-Dimethoxypyrimidine is a key structural motif commonly employed in herbicide molecular design in the quest for novel highly active herbicides^9^ (Fig. 1a). Many commercial herbicides contain this structural unit. For instance, bispyribac-sodium (BS) is an inhibitor of acetohydroxyacid synthase (AHAS; EC 2.2.1.6), the first enzyme involved in the branched-chain amino acid biosynthesis pathway.^10^ BS is a broad spectrum pyrimidinyl carboxy herbicide that has been used in the transplanted and direct seeded rice crops for selective post-emergence control of grasses, sedges and broad-leaved weeds.^10*a*,11^ Bensulfuron-methyl is also an AHAS-inhibiting herbicide belonging to the sulfonylurea class. It is a broad spectrum rice herbicide for pre-emergence or early post-emergence control of most broad-leaved grasses and sedges in transplanted or direct-seeded paddy rice.^12^ Another important herbicide containing 4,6-dimethoxypyrimidine scaffold is pyrithiobac-sodium, also an AHAS inhibitor, which is used for post-emergence control of broad-leaved weeds in cotton cultivation.^13^ Furthermore, pyribambenz-propyl is also a highly active herbicide belonging to the pyrimidinyloxybenzylamine class. It has been developed in China for post-emergence weed control primarily in oilseed rape.^14^

In addition to pyrimidine, other N-heterocycles have also received considerable attention because of their medicinal and agrochemical importance,^15^ including pyrazole, benzimidazole, indole, tetrahydrocarbazole, *etc.* (Fig. 1b). For example, topramezone, a safe and efficient HPPD-inhibiting herbicide, was developed by BASF in 2006 for use in corn field.^16^ Similarly, 2-trifluoromethylbenzimidazole derivatives also exhibit important herbicidal and insecticidal activities, such as chlorflurazole and fenazaflor.^17^ Indole represents one of the most important heterocyclic ring which provides privileged scaffolds in agrochemistry.^18^ Indole-3-acetic acid (IAA) is an important phytohormone,^19^ and thaxtomin A shows effective weed control ability and exerts no toxicity to rice.^20^ Moreover, tetrahydrocarbazole is a well-known privileged scaffold,^21^ possessing many different biological functions such as anti-tumor,^22^ antibiotics^23^ and anti-virus.^24^ Tetrahydrocarbazole also shows photosynthesis–inhibitory activity and acts as a herbicide prototype.^20*a*,25^ In light of the various biological activities of these N-heterocycles, there is a great need to develop an effective method to integrate the 4,6-dimethoxypyrimidine moiety with various N-heterocycles to obtain a new class of potential herbicidal agents.

Diversity-oriented synthesis (DOS), which aims to synthesize libraries of diverse small molecules in an efficient manner, is proved to be an efficient tool for the discovery of novel bioactive molecules in pharmaceutical and agrochemical chemistry.^26^ In DOS pathway, there are two principal methods for generating skeletal diversity, the reagent-based approach and the substrate-based approach.^27^ The reagent-based approach involves the use of a same starting material and different reaction conditions. In the substrate-based approach, a diverse array of substrates is subjected to the same reaction conditions to obtain diverse molecular skeletons.^28^ In this work, we used the substrate-based approach to create a N-heterocyclic library with diverse N-heterocyclic building blocks through diversity-oriented synthesis pathway.

In view of the significance of 4,6-dimethoxypyrimidine moiety and other bioactive N-heterocyclic skeletons, and as a continuation of our research on bioactive compounds,^1*a*,26*a*,29^ herein, we report the design and efficient synthesis of a novel pyrimidine–N-heterocycle hybrid library. 2-Hydroxybenzyl alcohol was used as the linkage inspired by pharmacophore structures of the commercial pyrimidine herbicides in Fig. 1a. A series of novel hybrids were synthesized by using a two-step process based on substrate-based approach, and their post-emergence herbicidal activity was investigated. Preliminary biological tests showed that some compounds exhibited good to significant herbicidal activity. To our knowledge, this diversity-oriented synthesis of pyrimidine–N-heterocycle hybrids has not been reported so far.

## Results and discussion

### Design

**Scheme 1:**
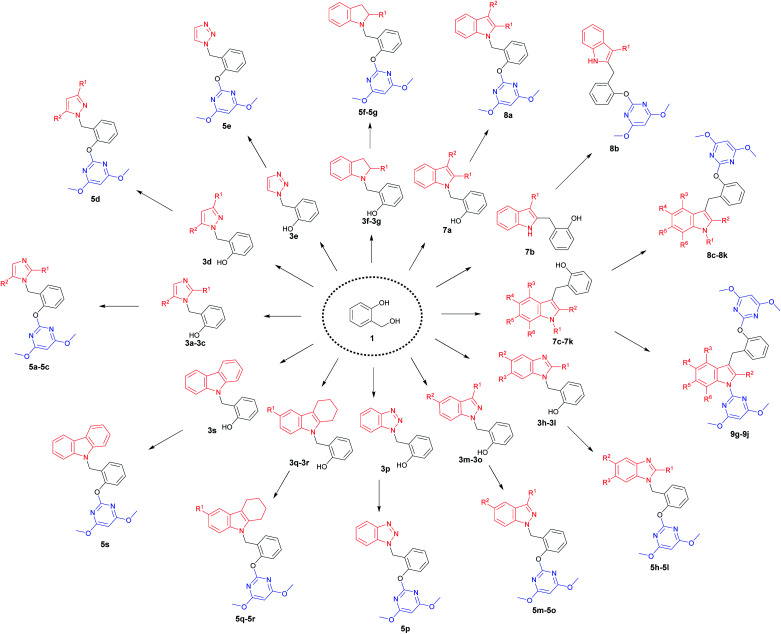
Diversity-based library obtained with a two-step diversity-oriented synthetic strategy.

A diversity-oriented synthetic strategy for the construction of the diversity-based library is illustrated in Scheme 1. 34 N-heterocyclic compounds containing 4,6-dimethoxypyrimidine moiety were achieved, which contained nine kinds of N-heterocyclic skeletons. Each final product was prepared from very simple, commercially available 2-hydroxybenzyl alcohol 1 in only two steps. This process involved *N*- or *C*-benzylation reactions between 2-hydroxybenzyl alcohol 1 and N-heterocycles, followed by the base-catalyzed S_N_Ar to give the final products. The compound library was made by using this efficient parallel synthetic techniques, where 28–241 mg of each final product were obtained. All library members were purified to ensure a high purity by thin-layer chromatography, and the compounds were fully characterized.

### Chemistry

We began the investigation with the synthesis of intermediates 3a–3s from 2-hydroxybenzyl alcohol 1. To test our hypothesis, the known *N*-benzylation reaction of 2-hydroxybenzyl alcohol 1 with imidazole 2a^30^ was chosen as a model to optimize the reaction conditions. As shown in Table 1, when a mixture of 1 and 2a was heated at 130 °C for 30 min under solvent-free condition, the desired product 3a was obtained in only 40% yield (Table 1, entry 1). To increase the yield, the reaction temperature was further investigated. The yield of the product 3a was effectively improved by elevating the temperature (Table 1, entries 2–4 *vs.* 1), while higher temperatures led to a significant decrease in yield (Table 1, entry 5). The reaction time was also surveyed, and 30 min was selected as the best option, giving 3a in 79% yield (Table 1, entries 6–8 *vs.* 4). In addition, we found that a slight excess of 2-hydroxybenzyl alcohol 1 was necessary to increase the yield (Table 1, entry 4 *vs.* 9). Therefore, the optimal conditions were observed with heating at 160 °C for 30 min under solvent-free condition using 1.2 equiv. of 2-hydroxybenzyl alcohol 1. 3a was then obtained in 60% isolated yield after recrystallization from an ethanol–DMF solvent mixture^31^ (Table 1, entry 4).

**Table 1:** Optimization of reaction conditions for intermediate 3a^a^

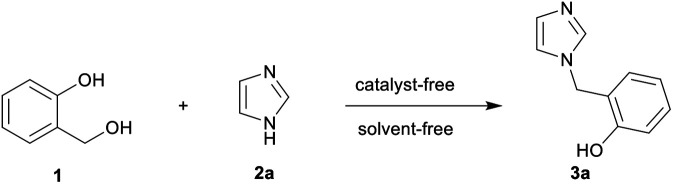				
Entry	Ratio^b^	*T* (°C)	Time (min)	Yield^c^ (%)
1	1 : 1.2	130	30	40
2	1 : 1.2	140	30	55
3	1 : 1.2	150	30	76
**4**	**1** : **1.2**	**160**	**30**	**79(60)** d
5	1 : 1.2	170	30	64
6	1 : 1.2	160	10	66
7	1 : 1.2	160	20	76
8	1 : 1.2	160	40	71
9	1 : 1.0	160	30	75

a The reactions were carried out with 4 mmol of 2a under solvent-free condition.

b The ratio of 2a : 1.

c The yields of 3a were determined by ^1^H NMR of the reaction mixture after work-up using 1,3,5-trimethoxybenzene as internal standard.

d Isolated yield.

With optimized reaction conditions in hand for the synthesis of 3a, we set out to explore the substrate scope of DOS in a two-step protocol (Table 2). In the first step, we tried to prepare the intermediate products 3*via* the *N*-benzylation reaction between 2-hydroxybenzyl alcohol 1 and N-heterocycles 2. It was found that the majority of N-heterocycles 2 were compatible with the optimal conditions, affording the desired products 3a–3s in useful isolated yields ranging from 19% to 77%. Both electron-donating (CH_3_) and electron-withdrawing (Cl, CF_3_) substituents on the N-heterocycles were tolerated under the same conditions. It was noteworthy to point out that 2.4 equiv. of 2-hydroxybenzyl alcohol 1 was required for carbazole 2s to reach the molten state.

**Table 2:** A two-step reaction sequence for the diversity-oriented-synthesis of pyrimidine–N-heterocycle hybrids 5^a^^,^^b^^,^^c^^,^^d^

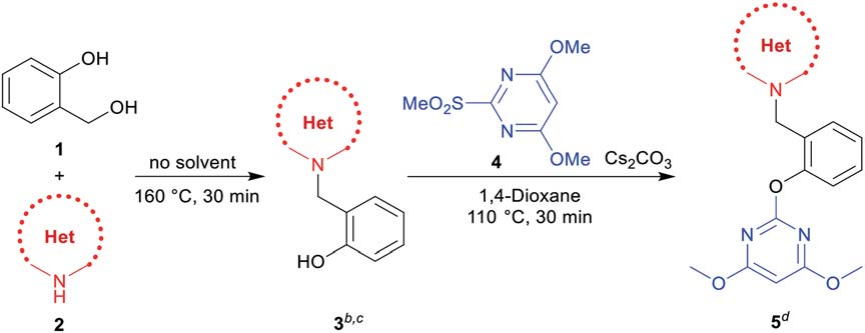
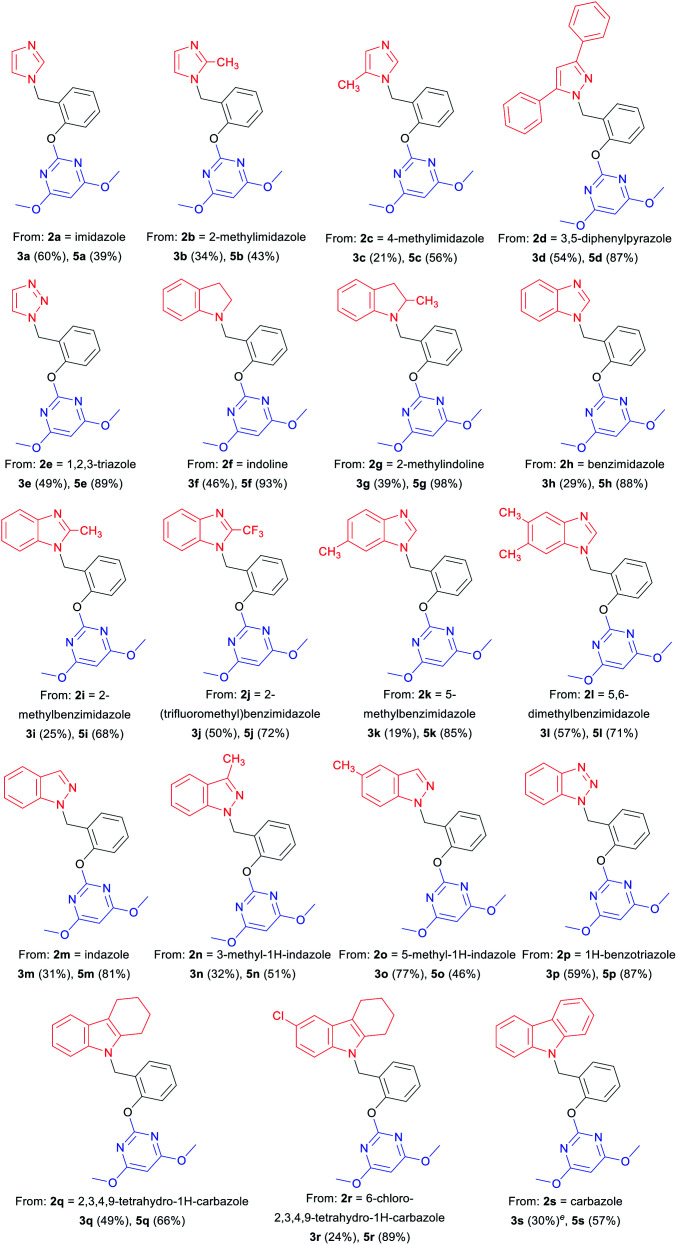

a Isolated yield.

b Reaction conditions: 2a–2e, 2h–2n, 2p (10 mmol), 1 (12 mmol), no solvent, 160 °C, 30 min.

c Reaction conditions: 2f–2g, 2o, 2q–2r (3 mmol), 1 (3.6 mmol), no solvent, 160 °C, 30 min.

d Reaction conditions: 3 (0.6 mmol), 4 (0.72 mmol), caesium carbonate (0.9 mmol), 1,4-dioxane (10 mL), 110 °C, 30 min.

e Reaction conditions: 2s (2 mmol), 1 (4.8 mmol), no solvent, 160 °C, 30 min.

Subsequently, we proceeded to evaluate the next transformation. As shown in Table 2, the reaction of the intermediate products 3 with 4,6-dimethoxy-2-(methylsulfonyl) pyrimidine 4 under our previously published S_N_Ar conditions,^1*a*^ afforded the desired products 5a–5s in modest to excellent yields (39–98%). An important advantage of this expeditious method is the ability to construct different pyrimidine–N-heterocycle hybrids 5 only by changing the N-heterocycle component in the first step, thus emphasizing the diversity-oriented feature of the protocol. For example, by using imidazoles, we obtained pyrimidine–imidazole hybrids 5a, 5b and 5c in 39%, 43% and 56% yields, respectively. The pyrimidine–pyrazole hybrid 5d and pyrimidine–1,2,3-triazole hybrid 5e were readily synthesized in 87% and 89% yields. With different N-fused heterocycles as substrates, a variety of pyrimidine–indoline hybrids 5f–5g, pyrimidine–benzimidazole hybrids 5h–5l, pyrimidine–indazoles 5m–5o, and pyrimidine–benzotriazole 5p could be smoothly prepared. Similarly, it was found that pyrimidine–carbazole hybrids 5q–5s were produced from corresponding carbazole derivatives. The successful construction of this DOS library further demonstrates the effectiveness and practicality of this new methodology.

Indoles are often found in natural products and synthetic compounds that exhibit a wide range of biological activity. Therefore, the divergent generation of indole derivatives with privileged heterocyclic structures is highly desirable and possesses great challenges. So we next expanded the scope of our DOS strategy towards the divergent synthesis of indole derivatives *via N*-benzylation, C-2 benzylation and C-3 benzylation at various points of indole ring, using the same two-step conditions shown as above (Table 3). We first designed and synthesized compound 8a*via N*-benzylation as a key step. 2,3-Dimethylindole 6a reacted with 2-hydroxybenzyl alcohol 1 under solvent-free condition to afford the intermediate 7a and then, reacted with 4 to generate the desired product 8a in 72% yield. Next, we employed C-2 reactive site of 3-methylindole 6b for the synthesis of 8b. In this case, the 2-position of indole was unoccupied, so the *C*-benzylation of 6b with 1 proceeded at the 2-position forming a C–C bond. Then, the S_N_Ar reaction of 7b with 4 produced 8b in 60% yield. Finally, we used C-3 reactive site of indoles 6c–6k for the synthesis of 8c–8k and 9g–9j. When the 3-position of indole was unoccupied, the *C*-benzylation of indole occurred first at 3-position to generate intermediates 7c–7k and the mechanism of the regioselectivity has been explained in a previous report.^32^ For intermediates 7c–7f, the S_N_Ar reactions worked efficiently and afforded the desired products 8c–8f in good yields ranging from 65 to 99%. However, when the methyl or chlorine groups were attached to C-4, 5 and 6 positions, the S_N_Ar reaction gave the mixtures of the mono-substituted pyrimidine compounds 8g–8j and the di-substituted pyrimidine compounds 9g–9j, while the yields of mono-substituted products were significantly higher than di-substituted products under the standard conditions. It is worth noting that when the C-7 position of indole contained a chlorine group, only mono-substituted pyrimidine product 8k was obtained. It is probably due to the steric effect of C-7 chlorine group on indole ring which hindered the formation of the di-substituted product. Collectively, we confirmed that our DOS strategy was suitable for the synthesis of pyrimidine–indole hybrids, which could be used to populate the areas of new chemical space.

**Table 3:** Diversity-oriented synthesis of pyrimidine–indole hybrids 8 and 9^a^^,^^b^

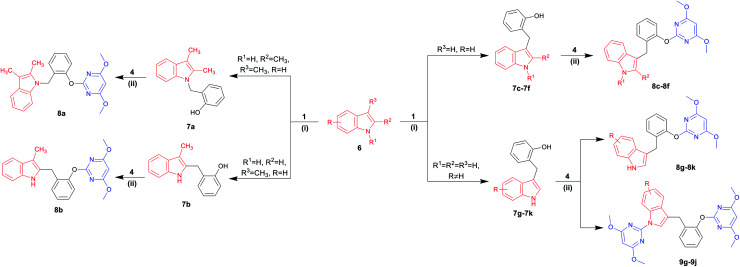
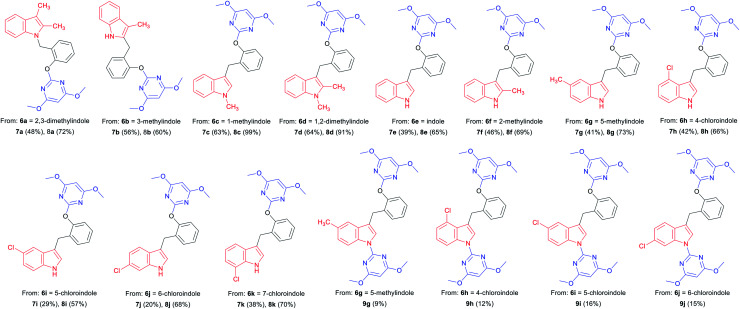

a Reaction conditions: (i) 6 (3 mmol), 1 (3.6 mmol), no solvent, 160 °C, 30 min; (ii) 7 (0.6 mmol), 4 (0.72 mmol), caesium carbonate (0.9 mmol), 1,4-dioxane (10 mL), 110 °C, 30 min.

b Isolated yield.

### Biological assessment

The post-emergence herbicidal activity of 34 title compounds was evaluated against several representative weeds at 750 g ai per ha under greenhouse conditions (see ESI Tables S2 and S3† for the full data set). On the basis of the preliminary bioassays, a selection of 11 active compounds was presented in Table 4. The herbicidal activity evaluation indicated that the active compounds showed good to excellent herbicidal activity against the test weeds. When the N-heterocycles were imidazole (5a), triazole (5e), benzimidazole (5j), indazoles (5n and 5o) and indoles (8e, 8i, 8k and 9i), compounds exhibited ≥60% inhibition against the tested monocotyledonous weeds such as *Digitaria sanguinalis* at the rate of 750 g ai per ha. It confirms that N-heterocyclic types, do have an effect on herbicidal activity. Among them, imidazole, triazole, benzimidazole, indazole and indole are the preferred N-heterocycles for maintaining herbicidal activity. The further understanding requires the study of the interaction between these active molecules and their possible target. Compound 8h with 4-chloroindole skeleton displayed over 60% control efficiency against the tested dicotyledonous weeds (*Abutilon theophrasti* and *Amaranthus retroflexus*) and the tested monocotyledonous weeds (*Digitaria sanguinalis*) as well. Compared with other herbicidal indoles 8e, 8i and 8k, 8h exhibited better herbicidal activity than they did. It suggests that C-3 benzylation of indole and chloro atom at 4-position on indole ring may be important factors for keeping herbicidal activity. The structure–activity relationships deserve further investigation. Very promisingly, compound 5q with tetrahydrocarbazole skeleton displayed over 60% inhibition against all tested weeds, and its inhibition rates against *Abutilon theophrasti* and *Cassia tora* were even over 80%. 5q showed higher herbicidal activity against dicotyledonous plants than monocotyledons for post-emergence application. Obviously, the tetrahydrocarbazole skeleton makes a critical contribution to the herbicidal activity. A possible explanation for the high herbicidal activity of compound 5q is that the presence of methylene groups at the tetrahydrocarbazole skeleton increases the compound's lipophilicity and facilitates their uptake by plants.^25^ In generally, the above results enable these active compounds to be of much potential for the optimization. Among them, compound 5q with high and broad spectrum herbicidal activity is the most promising candidate for the further development of new herbicides.

**Table 4:** Herbicidal activity of 11 active compounds^a^

Compound	Post-emergence, 750 g ai per ha				
	AT	EC	AR	DS	CT
5a	−	−	+	+++	+
5e	+	−	−	+++	+
5j	−	−	+	+++	+
5n	−	−	−	+++	+
5o	−	−	+	+++	+
5q	++++	+++	+++	+++	++++
8e	+	−	−	+++	+
8h	+++	+	+++	+++	+
8i	+	−	−	+++	−
8k	+	−	−	+++	−
9i	+	+	+	+++	−

a AT for *Abutilon theophrasti*; EC for *Echinochloa crusgalli*; AR for *Amaranthus retroflexus*; DS for *Digitaria sanguinalis*; CT for *Cassia tora*. Rating system for the growth inhibition percentage: ++++, ≥80%; +++, 60–79%; ++, 50–59%; +, 30–49%; −, <30%.

## Conclusions

In conclusion, this study has achieved the step-economical diversity-oriented synthesis of 34 novel target compounds 5, 8 and 9*via* a two-step process. These target compounds formed a pyrimidine–N-heterocycle hybrid library with prominent features of high structural diversity. More importantly, these novel hybrids have been subjected to the test of *in vivo* herbicidal activity, resulting in the finding that 11 active compounds exhibited good to excellent post-emergence herbicidal activity at 750 g ai per ha. Among them, 5q, a novel pyrimidine–tetrahydrocarbazole hybrid showed high and broad spectrum herbicidal activity against five test weeds, especially for effective control of *Abutilon theophrasti* and *Cassia tora*. 5q may become a novel lead compound for the further development of new herbicides. To further investigate the mechanism of these active compounds, enzymatic kinetics and molecular-modelling experiments are currently underway in our laboratory.

## Conflicts of interest

There are no conflicts to declare.

## Supplementary Material

RA-011-D1RA02663A-s001
