# Supplementary material for: Design, step-economical diversity-oriented synthesis of an N-heterocyclic library containing a pyrimidine moiety: discovery of novel potential herbicidal agents
Source: RSC Adv. 2021 Apr 26;11(25):15380–6. doi: 10.1039/d1ra02663a (PMC8698718; doi:10.1039/d1ra02663a)

Electronic Supplementary Material (ESI) for RSC Advances.
This journal is © The Royal Society of Chemistry 2021

Supporting information

Design, step-economical diversity-oriented synthesis of N-heterocyclic library containing a pyrimidine moiety: discovery of novel potential herbicidal agents

Dong Ma,[a] Yang Yin,[a] Ying-Lu Chen,[a] Yi-Tao Yan[a] and Jun Wu[*a]*

[a]Department of Chemistry, Zhejiang University, Hangzhou 310027, P. R. of China.

E-mail: wujunwu@zju.edu.cn

Table of contents

1. General information……………………………………………………………………...S2
2. Experimental Procedures……………………………………………………………………S3
3. Synthesis of target products and characterization data…………………………………...S6
4. Biological Testing………………………………………………………………………….S27
5. References…………………………………………………………………………….......S29
6. NMR spectra…………………………………………………………………………...S30

1. General information

Unless otherwise indicated, all reagents were purchased from commercial distributors and used without further purification. Column chromatography was performed using silica gel (300-400 mesh). Thin-layer chromatography (TLC) was carried out using silica gel GF254 plates. Nuclear magnetic resonance (NMR) spectra were recorded with a Bruker AVANCE 400 MHz instrument. ^{1}H, and ^{13}C chemical shifts are reported in ppm downfield of tetramethylsilane and referenced to residual solvent peak ($CHCl_3$ = 7.26 ppm for ^{1}H NMR, 77.16 ppm for ^{13}C NMR; DMSO-d_6 = 2.50 ppm for ^{1}H NMR, 39.52 ppm for ^{13}C NMR) unless otherwise noted. Multiplicities are reported using the following abbreviations: s = singlet, d = doublet, t = triplet, q = quartet, m = multiplet, dd = doublet of doublets. High resolution mass spectra (HRMS) were recorded on WATERS GCT Premier using EI-TOF or SHIMADZU LCMS-IT-TOF using ESI-TOF operating in positive ion mode. Melting points were determined using WRR apparatus and not corrected.

2. Experimental Procedures

2.1 Synthesis of intermediates 3a, 3h-3n and 3p

Example for the synthesis of **3a**:

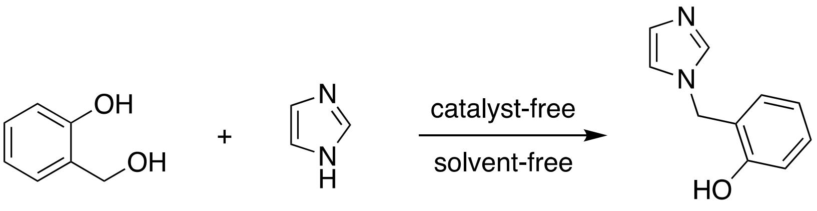

1 **2a** **3a**

A mixture of imidazole **2a** (10 mmol) and 2-hydroxybenzyl alcohol **1** (12 mmol) was heated at 160 °C with intense stirring for 30 min. Upon cooling, the reaction mass was washed with cold ethanol and then recrystallized from an ethanol - DMF mixture.[1, 2]

2.2 Synthesis of intermediates 3b-3e

Example for the synthesis of **3e**:

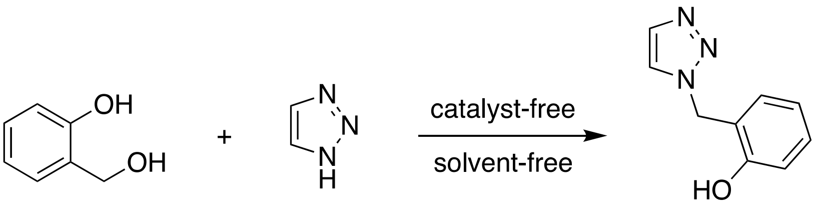

1 **2e** **3e**

A mixture of 1,2,3-triazole **2e** (10 mmol) and 2-hydroxybenzyl alcohol **1** (12 mmol) was heated at 160 °C with intense stirring for 30 min. Upon cooling, the reaction mass was washed with cold ethyl acetate and then recrystallized from an ethyl acetate - DMF mixture.

2.3 Synthesis of intermediates 3f-3g, 3o, 3q-3r and 7a-7k

Example for the synthesis of **3f**:

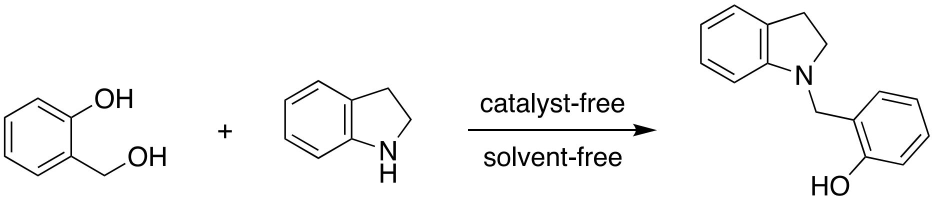

1 **2f** **3f**

A mixture of 1 indoline **2f** (3 mmol) and 2-hydroxybenzyl alcohol **1** (3.6 mmol) was heated at 160 °C with intense stirring for 30 min. Upon cooling, the reaction mass was isolated by column

chromatography on silica gel with petroleum ether - ethyl acetate (V/V=5/1) as the eluent to afford the desired products.

2.4 Synthesis of intermediate 3s

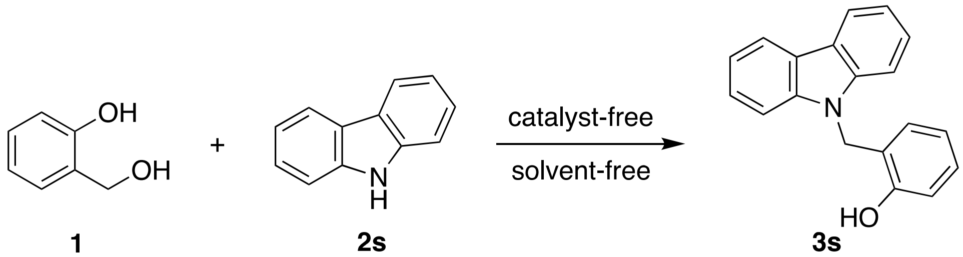

A mixture of 1 carbazole **2s** (2 mmol) and 2-hydroxybenzyl alcohol **1** (4.8 mmol) was heated at 160 °C with intense stirring for 30 min. Upon cooling, the reaction mass was isolated by column chromatography on silica gel with petroleum ether - ethyl acetate (V/V=5/1) as the eluent to afford the desired products.

Table S1 intermediates **3a-3s and 7a-7k**

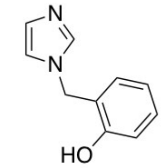	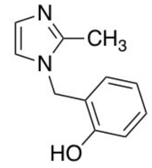	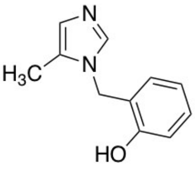	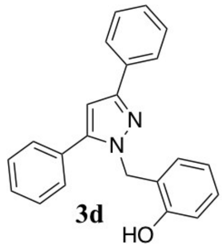	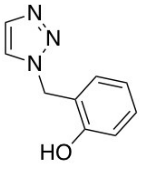
**3a**	**3b**	**3c**	**3d**	**3e**
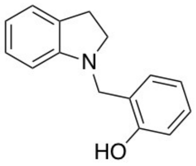	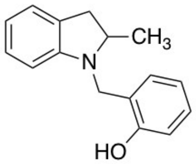	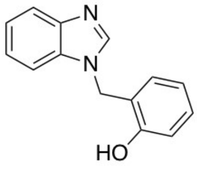	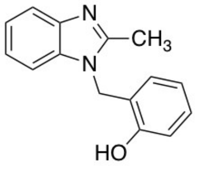	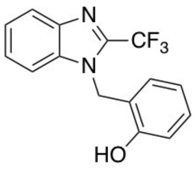
**3f**	**3g**	**3h**	**3i**	**3j**
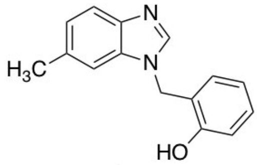	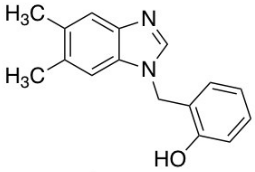	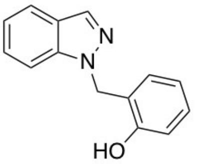	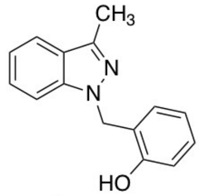	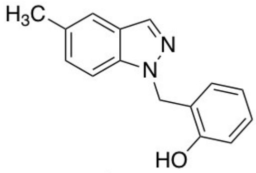
**3k**	**3l**	**3m**	**3n**	**3o**
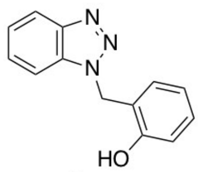	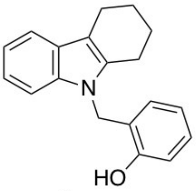	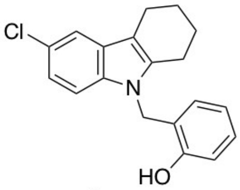	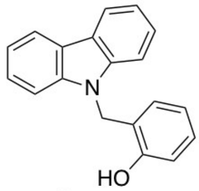	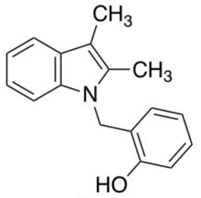
**3p**	**3q**	**3r**	**3s**	**7a**
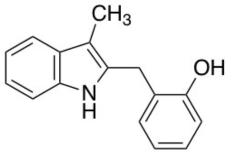	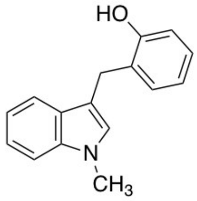	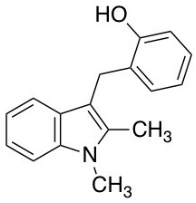	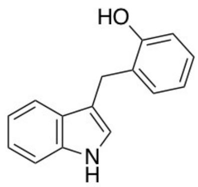	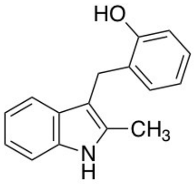
**7b**	**7c**	**7d**	**7e**	**7f**
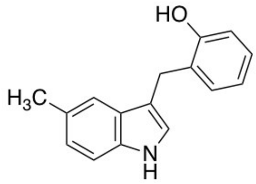	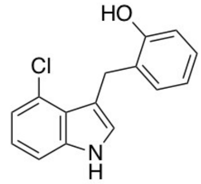	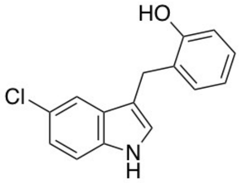	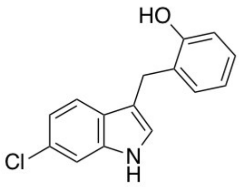	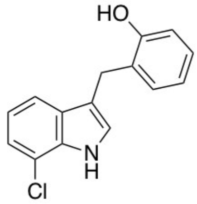
**7g**	**7h**	**7i**	**7j**	**7k**

3. Synthesis of target products and characterization data

3.1 General procedure A for the synthesis of products 5a-5c, 5h, 5i, 5k and 5l

Example for the synthesis of **5a**:

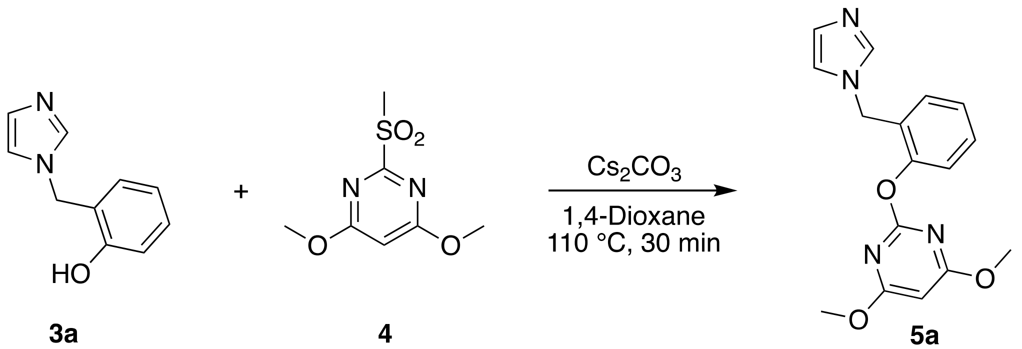

General procedure A: To a 50 mL round-bottom flask, were added **3a** (0.6 mmol), **4** (0.72 mmol), Cs_2CO_3 (0.9 mmol) and 1,4-dioxane (10 mL). The mixture was heated at 110 °C for 30 min until complete consumption of **3a**. Afterwards, the reaction mixture was quenched with brine, the aqueous layer was extracted with ethyl acetate (3×10 mL), and combined organic extracts were dried over Na_2SO_4.[3] After filtration and evaporation of the solvents under vacuum, the crude product was purified by thin-layer chromatography on silica gel with dichloromethane - methanol (V/V=40/1) as the developing agent to afford the desired product **5a**.

Analytical data for the products **5a-5c**, **5h**, **5i**, **5k** and **5l**:

2-(2-((1*H*-imidazol-1-yl)methyl)phenoxy)-4,6-dimethoxypyrimidine

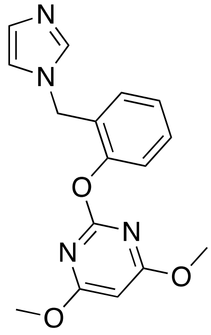

5a

The title compound **5a** was prepared according to general procedure A. **5a** was obtained as colorless liquid (73 mg, yield 39%). ^1H NMR (400 MHz, $CDCl_3$) δ 7.90 (s, 1H), 7.41 – 7.36 (m, 1H), 7.24 – 7.17 (m, 3H), 7.05 (s, 1H), 6.96 (s, 1H), 5.78 (s, 1H), 5.20 (s, 2H), 3.79 (s, 6H). ^{13}C NMR (101 MHz, $CDCl_3$) δ 173.09, 163.87, 150.91, 137.19, 129.85, 129.32, 128.35, 127.87, 126.17, 123.23, 119.73, 85.13, 54.41, 46.36. HRMS m/z (EI-TOF): calcd for $C_{16}H_{16}N_4O_3$ (M^+): 312.1222; found 312.1219.

4,6-dimethoxy-2-(2-((2-methyl-1*H*-imidazol-1-yl)methyl)phenoxy)pyrimidine

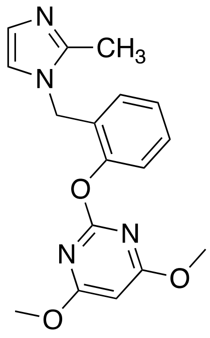

5b

The title compound **5b** was prepared according to general procedure A. **5b** was obtained as colorless liquid (84 mg, yield 43%). ^{1}H NMR (400 MHz, $CDCl_3$) δ 7.37 – 7.32 (m, 1H), 7.21 – 7.16 (m, 2H), 6.91 – 6.80 (m, 3H), 5.79 (s, 1H), 5.05 (s, 2H), 3.80 (s, 6H), 2.32 (s, 3H). ^{13}C NMR (101 MHz, $CDCl_3$) δ 173.11, 163.84, 150.49, 145.09, 129.22, 128.90, 128.04, 126.79, 126.09, 123.01, 120.21, 85.12, 54.38, 45.26, 12.99. HRMS m/z (EI-TOF): calcd for $C_{17}H_{18}N_4O_3$ (M^+): 326.1379; found 326.1380.

4,6-dimethoxy-2-(2-((5-methyl-1*H*-imidazol-1-yl)methyl)phenoxy)pyrimidine

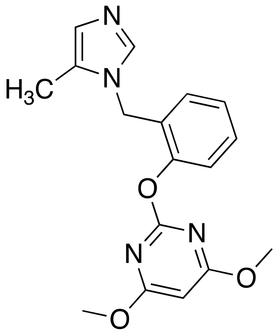

5c

The title compound **5c** was prepared according to general procedure A. **5c** was obtained as colorless liquid (109 mg, yield 56%). ^{1}H NMR (400 MHz, $CDCl_3$) δ 7.85 (s, 1H), 7.38 (td, J = 7.8, 1.5 Hz, 1H), 7.24 – 7.18 (m, 2H), 6.95 (d, J = 7.4 Hz, 1H), 6.85 (s, 1H), 5.77 (s, 1H), 5.09 (s, 2H), 3.80 (s, 6H), 2.12 (s, 3H). ^{13}C NMR (101 MHz, $CDCl_3$) δ 173.13, 163.68, 150.68, 129.89, 128.90, 128.43, 127.30, 126.29, 123.23, 85.24, 54.53, 44.67, 9.32. HRMS m/z (EI-TOF): calcd for $C_{17}H_{18}N_4O_3$ (M^+): 326.1379; found 326.1380.

1-(2-((4,6-dimethoxypyrimidin-2-yl)oxy)benzyl)-1*H*-benzo[*d*]imidazole

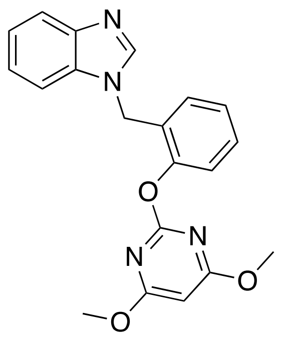

5h

The title compound **5h** was prepared according to general procedure A. **5h** was obtained as colorless liquid (192 mg, yield 88%). ^1H NMR (400 MHz, $CDCl_3$) δ 7.94 (s, 1H), 7.74 (d, *J* = 7.2 Hz, 1H), 7.38 – 7.31 (m, 2H), 7.23 – 7.13 (m, 5H), 5.70 (s, 1H), 5.37 (s, 2H), 3.70 (s, 6H). ^{13}C NMR (101 MHz, $CDCl_3$) δ 172.92, 163.79, 151.06, 143.43, 133.83, 129.64, 129.09, 128.00, 126.03, 123.38, 123.06, 122.32, 120.26, 110.23, 85.08, 54.27, 44.42. HRMS m/z (EI-TOF): calcd for $C_{20}H_{18}N_4O_3$ (M^+): 362.1379; found 362.1380.

1-(2-((4,6-dimethoxypyrimidin-2-yl)oxy)benzyl)-2-methyl-1*H*-benzo[*d*]imidazole

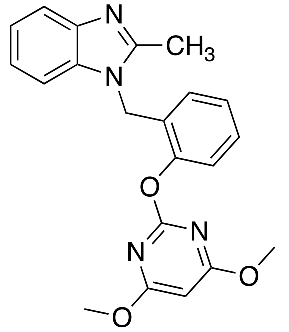

5i

The title compound **5i** was prepared according to general procedure A. **5i** was obtained as white solid (153 mg, yield 68%; mp: 114.1-115.0 °C). ^1H NMR (400 MHz, $CDCl_3$) δ 7.71 (d, *J* = 7.7 Hz, 1H), 7.38 – 7.33 (m, 1H), 7.30 – 7.20 (m, 3H), 7.16 (dd, *J* = 15.0, 7.7 Hz, 2H), 6.88 (d, *J* = 7.5 Hz, 1H), 5.74 (s, 1H), 5.37 (s, 2H), 3.75 (s, 6H), 2.69 (s, 3H). ^{13}C NMR (101 MHz, $CDCl_3$) δ 173.02, 163.58, 151.66, 150.74, 134.24, 129.65, 127.88, 127.18, 126.16, 123.51, 123.49, 123.36, 117.92, 110.37, 85.07, 54.38, 43.68, 13.46. HRMS m/z (EI-TOF): calcd for $C_{21}H_{20}N_4O_3$ (M^+): 376.1535; found 376.1535.

1-(2-((4,6-dimethoxypyrimidin-2-yl)oxy)benzyl)-6-methyl-1*H*-benzo[*d*]imidazole

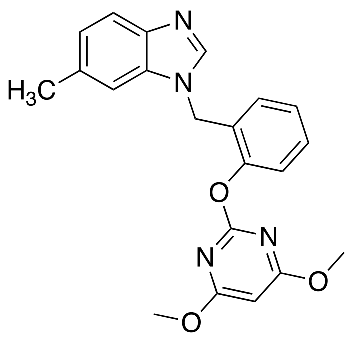

5k

The title compound **5k** was prepared according to general procedure A. **5k** was obtained as white solid (191 mg, yield 85%; mp: 127.8-128.9 °C). ^{1}H NMR (400 MHz, $CDCl_3$) δ 8.35 (s, 1H), 7.67 (d, J = 8.3 Hz, 1H), 7.40 – 7.35 (m, 1H), 7.23 – 7.16 (m, 4H), 7.12 (d, J = 8.3 Hz, 1H), 5.70 (s, 1H), 5.41 (s, 2H), 3.74 (s, 6H), 2.43 (s, 3H). ^{13}C NMR (101 MHz, $CDCl_3$) δ 173.00, 163.78, 151.06, 142.33, 134.01, 133.52, 129.90, 129.19, 127.56, 126.19, 125.00, 123.38, 118.89, 110.33, 85.07, 54.36, 44.52, 21.90. HRMS m/z (EI-TOF): calcd for $C_{21}H_{20}N_4O_3$ (M^+): 376.1535; found 376.1534.

1-(2-((4,6-dimethoxypyrimidin-2-yl)oxy)benzyl)-5,6-dimethyl-1*H*-benzo[*d*]imidazole

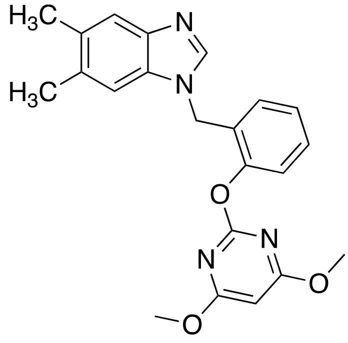

5l

The title compound **5l** was prepared according to general procedure A. **5l** was obtained as white solid (166 mg, yield 71%; mp: 153.3-154.3 °C). ^{1}H NMR (400 MHz, $CDCl_3$) δ 7.85 (s, 1H), 7.51 (s, 1H), 7.37 – 7.32 (m, 1H), 7.20 – 7.14 (m, 2H), 7.07 (d, J = 6.5 Hz, 2H), 5.72 (s, 1H), 5.32 (s, 2H), 3.74 (s, 6H), 2.33 (s, 3H), 2.30 (s, 3H). ^{13}C NMR (101 MHz, $CDCl_3$) δ 173.01, 163.86, 150.86, 142.61, 132.38, 131.37, 129.47, 128.75, 128.37, 126.08, 123.23, 120.13, 110.27, 85.02, 54.30, 44.10, 20.66, 20.34. HRMS m/z (ESI-TOF): calcd for $C_{22}H_{22}N_4O_3$ $(M+H)^+$: 391.1765; found 391.1765.

3.2 General procedure B for the synthesis of products 5d, 5m-5p, 8a-8b, 8e-8f and 8k

Example for the synthesis of **5d**:

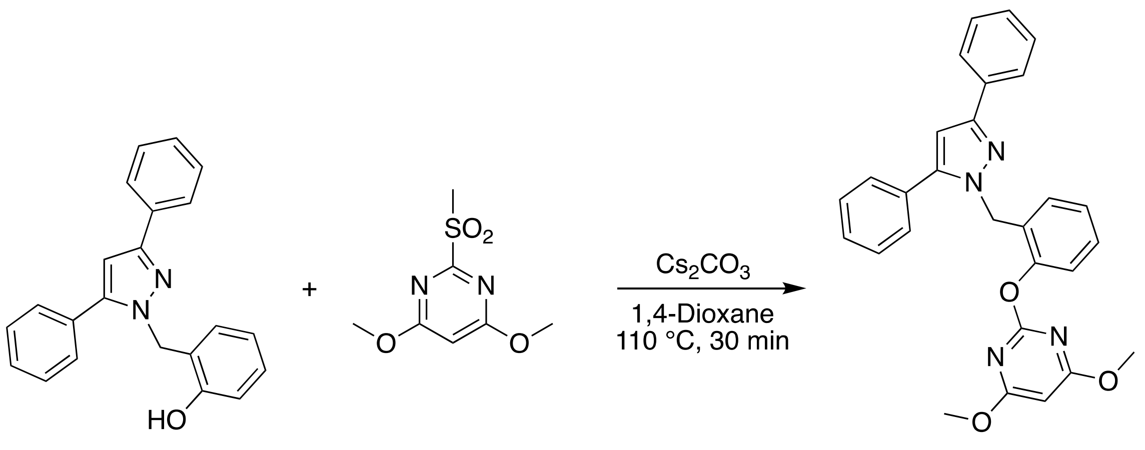

3d **4** **5d**

General procedure B: To a 50 mL round-bottom flask, were added **3d** (0.6 mmol), **4** (0.72 mmol), Cs_2CO_3 (0.9 mmol) and 1,4-dioxane (10 mL). The mixture was heated at 110 °C for 30 min until complete consumption of **3d**, as detected by TLC. Afterwards, the reaction mixture was quenched with brine, the aqueous layer was extracted with ethyl acetate (3×10 mL), and combined organic extracts were dried over Na_2SO_4. After filtration and evaporation of the solvents under vacuum, the crude product was purified by thin-layer chromatography on silica gel with petroleum ether - ethyl acetate (V/V=5/1) as the developing agent to afford the desired product **5d**.

Analytical data for the products **5d**, **5m-5p**, **8a-8b**, **8e-8f** and **8k**:

2-(2-((3,5-diphenyl-1*H*-pyrazol-1-yl)methyl)phenoxy)-4,6-dimethoxypyrimidine

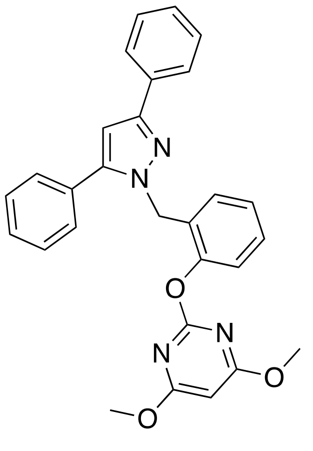

5d

The title compound **5d** was prepared according to general procedure B. **5d** was obtained as white solid (241 mg, yield 87%; mp: 149.4-149.8 °C). ^1H NMR (400 MHz, $CDCl_3$) δ 7.88 – 7.82 (m, 2H), 7.41 (t, *J* = 7.5 Hz, 2H), 7.37 – 7.27 (m, 7H), 7.17 – 7.10 (m, 2H), 6.97 (d, *J* = 6.9 Hz, 1H), 6.67 (s, 1H), 5.73 (s, 1H), 5.49 (s, 2H), 3.75 (s, 6H). ^{13}C NMR (101 MHz, $CDCl_3$) δ 172.96, 163.85, 150.99, 149.81, 145.99, 133.16, 130.45, 130.26, 128.82, 128.77, 128.75, 128.69, 128.38, 128.02, 127.99, 125.88,

125.84, 122.52, 103.79, 84.97, 54.33, 48.97. HRMS m/z (EI-TOF): calcd for $C_{28}H_{24}N_4O_3$ (M^+): 464.1848; found 464.1850.

1-(2-((4,6-dimethoxypyrimidin-2-yl)oxy)benzyl)-1*H*-indazole

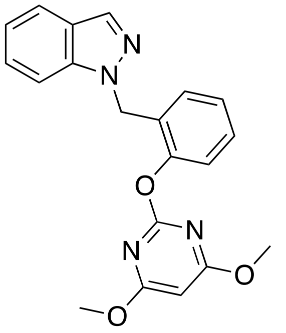

5m

The title compound **5m** was prepared according to general procedure B. **5m** was obtained as colorless liquid (176 mg, yield 81%). ^{1}H NMR (400 MHz, $CDCl_3$) δ 7.97 (s, 1H), 7.68 (d, *J* = 8.1 Hz, 1H), 7.35 (d, *J* = 8.4 Hz, 1H), 7.32 – 7.26 (m, 2H), 7.16 – 7.07 (m, 3H), 7.06 – 7.01 (m, 1H), 5.69 (s, 1H), 5.62 (s, 2H), 3.74 (s, 6H). ^{13}C NMR (101 MHz, $CDCl_3$) δ 172.87, 164.03, 150.52, 139.60, 133.32, 129.55, 129.03, 128.92, 126.40, 125.95, 124.12, 122.85, 121.07, 120.76, 109.62, 84.86, 54.27, 47.99. HRMS m/z (EI-TOF): calcd for $C_{20}H_{18}N_4O_3$ (M^+): 362.1379; found 362.1378.

1-(2-((4,6-dimethoxypyrimidin-2-yl)oxy)benzyl)-3-methyl-1*H*-indazole

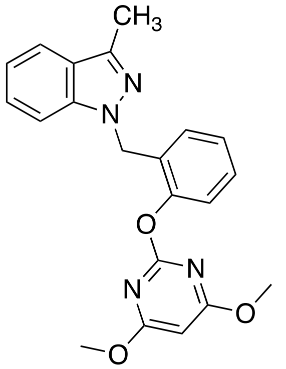

5n

The title compound **5n** was prepared according to general procedure B. **5n** was obtained as white solid (115 mg, yield 51%; mp: 103.2-104.0 °C). ^{1}H NMR (400 MHz, DMSO-d_6) δ 7.60 (d, *J* = 8.1 Hz, 1H), 7.46 (d, *J* = 8.5 Hz, 1H), 7.37 – 7.31 (m, 1H), 7.26 (t, *J* = 7.6 Hz, 1H), 7.23 – 7.14 (m, 3H), 7.03 (t, *J* = 7.4 Hz, 1H), 5.83 (s, 1H), 5.49 (s, 2H), 3.61 (s, 6H), 2.35 (s, 3H). ^{13}C NMR (101 MHz, DMSO-d_6) δ 172.36, 163.56, 150.44, 140.42, 139.98, 129.73, 128.95, 125.82, 125.66, 122.95, 122.75, 120.01, 119.50, 109.57, 83.68, 53.95, 47.17, 11.52. HRMS m/z (EI-TOF): calcd for $C_{21}H_{20}N_4O_3$ (M^+):

376.1535; found 376.1533.

1-(2-((4,6-dimethoxypyrimidin-2-yl)oxy)benzyl)-5-methyl-1*H*-indazole

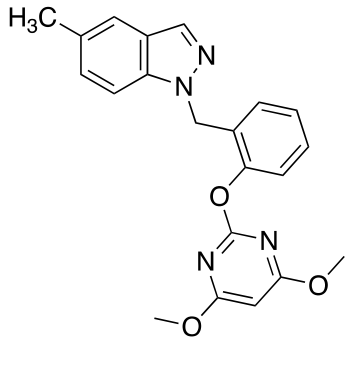

5o

The title compound **5o** was prepared according to general procedure B. **5o** was obtained as colorless liquid (103 mg, yield 46%). ^{1}H NMR (400 MHz, $CDCl_3$) δ 7.88 (s, 1H), 7.43 (s, 1H), 7.29 (d, J = 7.3 Hz, 1H), 7.23 (d, J = 8.6 Hz, 1H), 7.15 – 7.08 (m, 3H), 6.99 (d, J = 7.4 Hz, 1H), 5.70 (s, 1H), 5.59 (s, 2H), 3.75 (s, 6H), 2.41 (s, 3H). ^{13}C NMR (101 MHz, $CDCl_3$) δ 172.91, 164.04, 150.46, 138.37, 132.71, 130.15, 129.74, 128.88, 128.81, 128.50, 125.93, 124.57, 122.79, 120.02, 109.31, 84.86, 54.26, 48.01, 21.36. HRMS m/z (ESI-TOF): calcd for $C_{21}H_{20}N_4O_3$ $(M+H)^+$: 377.1608; found 377.1606.

1-(2-((4,6-dimethoxypyrimidin-2-yl)oxy)benzyl)-1*H*-benzo[*d*][1,2,3]triazole

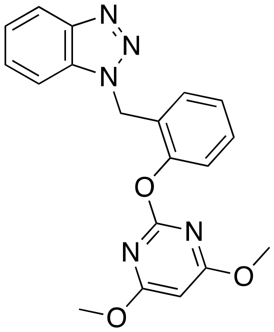

5p

The title compound **5p** was prepared according to general procedure B. **5p** was obtained as colorless liquid (189 mg, yield 87%). ^{1}H NMR (400 MHz, $CDCl_3$) δ 7.99 (d, J = 8.2 Hz, 1H), 7.44 (d, J = 8.1 Hz, 1H), 7.37 – 7.28 (m, 3H), 7.20 – 7.14 (m, 3H), 5.86 (s, 2H), 5.70 (s, 1H), 3.72 (s, 6H). ^{13}C NMR (101 MHz, $CDCl_3$) δ 172.93, 163.92, 150.78, 146.10, 132.95, 129.71, 129.48, 127.65, 127.30, 126.14, 123.92, 123.13, 119.96, 110.04, 85.07, 54.29, 47.08. HRMS m/z (EI-TOF): calcd for $C_{19}H_{17}N_5O_3$ (M^+): 363.1331; found 363.1330.

1-(2-((4,6-dimethoxypyrimidin-2-yl)oxy)benzyl)-2,3-dimethyl-1*H*-indole

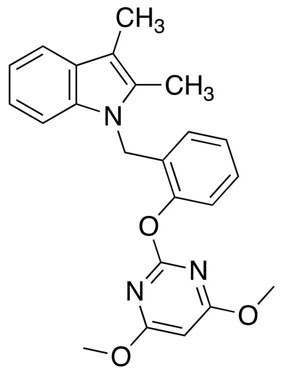

8a

The title compound **8a** was prepared according to general procedure B. **8a** was obtained as pink solid (168 mg, yield 72%; mp: 131.7-132.5 °C). ^{1}H NMR (400 MHz, CDCl$_3$) δ 7.51 – 7.46 (m, 1H), 7.26 – 7.22 (m, 1H), 7.15 – 7.10 (m, 2H), 7.08 – 7.00 (m, 3H), 6.49 (d, *J* = 7.1 Hz, 1H), 5.79 (s, 1H), 5.27 (s, 2H), 3.80 (s, 6H), 2.25 (s, 3H), 2.25 (s, 3H). ^{13}C NMR (101 MHz, CDCl$_3$) δ 173.05, 164.04, 150.13, 136.43, 132.62, 130.77, 128.74, 128.20, 127.20, 126.00, 122.56, 120.74, 118.90, 118.04, 108.95, 107.04, 84.93, 54.30, 42.22, 10.20, 9.00. HRMS m/z (EI-TOF): calcd for $C_{23}H_{23}N_3O_3$ (M^+): 389.1739; found 389.1737.

2-(2-((4,6-dimethoxypyrimidin-2-yl)oxy)benzyl)-3-methyl-1*H*-indole

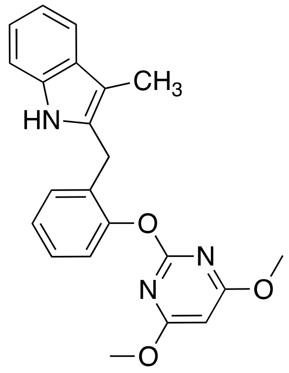

8b

The title compound **8b** was prepared according to general procedure B. **8b** was obtained as yellow liquid (136 mg, yield 60%). ^{1}H NMR (400 MHz, CDCl$_3$) δ 8.42 (s, 1H), 7.45 (d, *J* = 7.1 Hz, 1H), 7.29 – 7.24 (m, 1H), 7.23 – 7.19 (m, 1H), 7.18 – 7.12 (m, 3H), 7.09 – 7.00 (m, 2H), 5.70 (s, 1H), 4.04 (s, 2H), 3.84 (s, 6H), 2.28 (s, 3H). ^{13}C NMR (101 MHz, CDCl$_3$) δ 173.23, 164.39, 151.02, 135.54, 133.02, 132.00, 130.99, 129.05, 127.98, 126.19, 122.84, 121.16, 118.84, 118.25, 110.35, 107.47, 83.99, 54.62, 26.68, 8.66. HRMS m/z (EI-TOF): calcd for $C_{22}H_{21}N_3O_3$ (M^+): 375.1583; found 375.1585.

3-(2-((4,6-dimethoxypyrimidin-2-yl)oxy)benzyl)-1*H*-indole

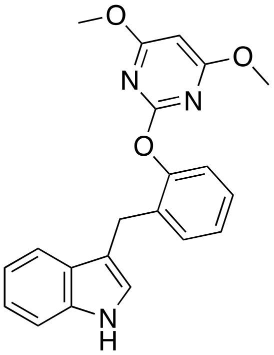

8e

The title compound **8e** was prepared according to general procedure B. **8e** was obtained as yellow liquid (141 mg, yield 65%). ^{1}H NMR (400 MHz, CDCl$_3$) δ 7.91 (s, 1H), 7.48 (d, *J* = 7.8 Hz, 1H), 7.31 (d, *J* = 8.1 Hz, 1H), 7.26 – 7.18 (m, 2H), 7.17 – 7.08 (m, 3H), 7.02 (t, *J* = 7.4 Hz, 1H), 6.92 (s, 1H), 5.71 (s, 1H), 4.08 (s, 2H), 3.74 (s, 6H). ^{13}C NMR (101 MHz, CDCl$_3$) δ 172.93, 164.45, 151.17, 136.42, 133.71, 130.37, 127.64, 127.02, 125.54, 122.91, 122.71, 121.98, 119.39, 119.30, 114.29, 111.09, 84.40, 54.25, 25.97. HRMS m/z (EI-TOF): calcd for $C_{21}H_{19}N_3O_3$ (M^+): 361.1426; found 361.1427.

3-(2-((4,6-dimethoxypyrimidin-2-yl)oxy)benzyl)-2-methyl-1*H*-indole

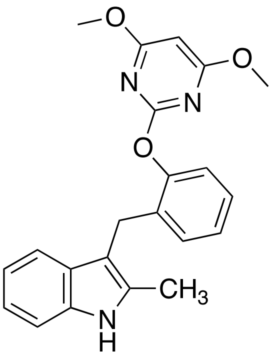

8f

The title compound **8f** was prepared according to general procedure B. **8f** was obtained as yellow liquid (155 mg, yield 69%). ^{1}H NMR (400 MHz, CDCl$_3$) δ 7.75 (s, 1H), 7.31 (d, *J* = 7.8 Hz, 1H), 7.24 – 7.17 (m, 2H), 7.12 – 7.04 (m, 4H), 6.99 – 6.93 (m, 1H), 5.72 (s, 1H), 4.01 (s, 2H), 3.74 (s, 6H), 2.33 (s, 3H). ^{13}C NMR (101 MHz, CDCl$_3$) δ 172.92, 164.41, 151.17, 135.37, 133.73, 132.11, 129.89, 129.12, 126.81, 125.47, 122.50, 120.99, 119.18, 118.61, 110.12, 109.12, 84.43, 54.15, 24.59, 11.88. HRMS m/z (EI-TOF): calcd for $C_{22}H_{21}N_3O_3$ (M^+): 375.1583; found 375.1585.

7-chloro-3-(2-((4,6-dimethoxypyrimidin-2-yl)oxy)benzyl)-1*H*-indole

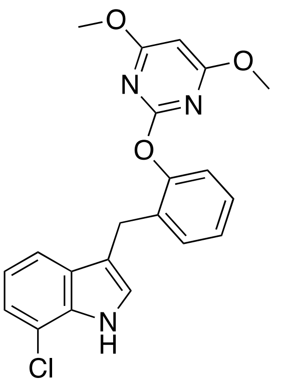

8k

The title compound **8k** was prepared according to general procedure B. **8k** was obtained as colorless liquid (167 mg, yield 70%). ^1H NMR (400 MHz, $CDCl_3$) δ 8.13 (s, 1H), 7.36 (d, J = 7.9 Hz, 1H), 7.25 – 7.20 (m, 2H), 7.12 (t, J = 7.8 Hz, 3H), 7.02 (s, 1H), 6.94 (t, J = 7.8 Hz, 1H), 5.70 (s, 1H), 4.07 (s, 2H), 3.73 (s, 6H). ^{13}C NMR (101 MHz, $CDCl_3$) δ 172.87, 164.22, 151.12, 133.62, 133.30, 130.32, 129.08, 127.24, 125.66, 123.60, 122.86, 121.33, 120.12, 118.10, 116.57, 115.51, 84.29, 54.31, 26.19. HRMS m/z (EI-TOF): calcd for $C_{21}H_{18}N_3O_3Cl$ (M^+): 395.1037; found 395.1038.

3.3 General procedure C for the synthesis of products 5e and 5s

Example for the synthesis of **5e**:

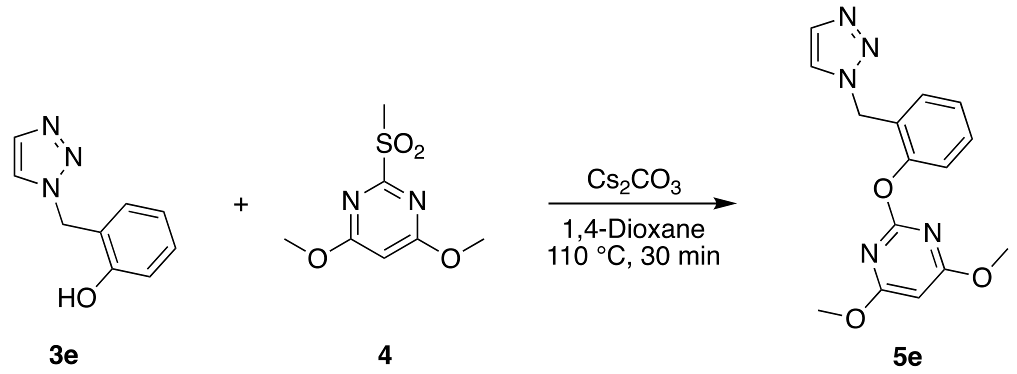

General procedure C: To a 50 mL round-bottom flask, were added **3e** (0.6 mmol), **4** (0.72 mmol), Cs_2CO_3 (0.9 mmol) and 1,4-dioxane (10 mL). The mixture was heated at 110 °C for 30 min until complete consumption of **3e**, as detected by TLC. Afterwards, the reaction mixture was quenched with brine, the aqueous layer was extracted with ethyl acetate (3×10 mL), and combined organic extracts were dried over Na_2SO_4. After filtration and evaporation of the solvents under vacuum, the crude product was purified by thin-layer chromatography on silica gel with petroleum ether - ethyl acetate (V/V=2/1) as the developing agent to afford the desired product **5e**.

Analytical data for the products **5e** and **5s**:

2-(2-((1*H*-1,2,3-triazol-1-yl)methyl)phenoxy)-4,6-dimethoxypyrimidine

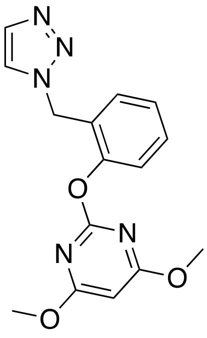

5e

The title compound **5e** was prepared according to general procedure C. **5e** was obtained as colorless liquid (167 mg, yield 89%). ^{1}H NMR (400 MHz, $CDCl_3$) δ 7.65 (s, 1H), 7.57 (s, 1H), 7.42 – 7.36 (m, 1H), 7.28 (dd, *J* = 7.8, 1.8 Hz, 1H), 7.25 – 7.18 (m, 2H), 5.79 (s, 1H), 5.58 (s, 2H), 3.79 (s, 6H). ^{13}C NMR (101 MHz, $CDCl_3$) δ 173.13, 163.91, 150.97, 133.61, 130.17, 130.14, 127.32, 126.27, 124.12, 123.11, 85.16, 54.44, 49.08. HRMS m/z (ESI-TOF): calcd for $C_{15}H_{15}N_5O_3$ $(M+Na)^+$: 336.1067; found 336.1067.

9-(2-((4,6-dimethoxypyrimidin-2-yl)oxy)benzyl)-9*H*-carbazole

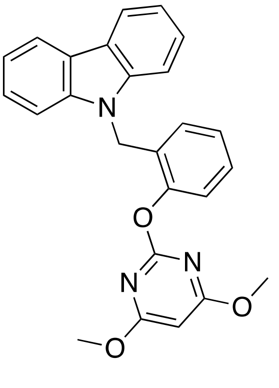

5s

The title compound **5s** was prepared according to general procedure C. **5s** was obtained as white solid (140 mg, yield 57%; mp: 153.9-154.8 °C). ^{1}H NMR (400 MHz, $CDCl_3$) δ 8.07 (d, *J* = 7.7 Hz, 2H), 7.42 – 7.37 (m, 2H), 7.35 (d, *J* = 8.0 Hz, 2H), 7.28 (d, *J* = 7.5 Hz, 1H), 7.24 – 7.19 (m, 2H), 7.18 – 7.13 (m, 1H), 7.03 (t, *J* = 7.5 Hz, 1H), 6.84 (d, *J* = 7.0 Hz, 1H), 5.72 (s, 1H), 5.52 (s, 2H), 3.73 (s, 6H). ^{13}C NMR (101 MHz, $CDCl_3$) δ 172.88, 163.85, 150.73, 140.71, 129.61, 128.59, 127.88, 125.96, 125.85, 123.02, 122.91, 120.38, 119.26, 109.18, 84.80, 54.30, 42.34. HRMS m/z (EI-TOF): calcd for $C_{25}H_{21}N_3O_3$ (M^+): 411.1583; found 411.1585.

3.4 General procedure D for the synthesis of products 5f and 5g

Example for the synthesis of **5f**:

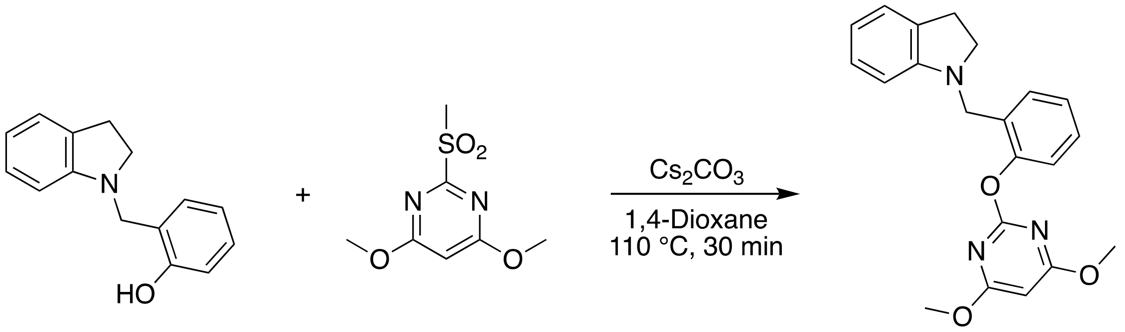

3f **4** **5f**

General procedure D: To a 50 mL round-bottom flask, were added **3f** (0.6 mmol), **4** (0.72 mmol), Cs_2CO_3 (0.9 mmol) and 1,4-dioxane (10 mL). The mixture was heated at 110 °C for 30 min until complete consumption of **3f**, as detected by TLC. Afterwards, the reaction mixture was quenched with brine, the aqueous layer was extracted with ethyl acetate (3×10 mL), and combined organic extracts were dried over Na_2SO_4. After filtration and evaporation of the solvents under vacuum, the crude product was purified by thin-layer chromatography on silica gel with petroleum ether - ethyl acetate (V/V=20/1) as the developing agent to afford the desired product **5f**.

Analytical data for the products **5f** and **5g**:

1-(2-((4,6-dimethoxypyrimidin-2-yl)oxy)benzyl)indoline

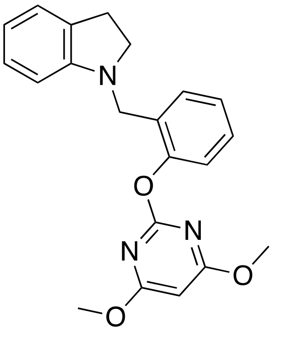

5f

The title compound **5f** was prepared according to general procedure D. **5f** was obtained as pink liquid (203 mg, yield 93%). ^{1}H NMR (400 MHz, $CDCl_3$) δ 7.50 (d, *J* = 7.6 Hz, 1H), 7.31 (td, *J* = 7.8, 1.6 Hz, 1H), 7.21 (td, *J* = 7.5, 1.1 Hz, 1H), 7.16 (dd, *J* = 8.0, 1.0 Hz, 1H), 7.04 (d, *J* = 7.2 Hz, 1H), 7.00 (t, *J* = 7.7 Hz, 1H), 6.65 (t, *J* = 7.3 Hz, 1H), 6.54 – 6.37 (m, 1H), 5.72 (s, 1H), 4.24 (s, 2H), 3.78 (s, 6H), 3.34 (t, *J* = 8.2 Hz, 2H), 2.89 (t, *J* = 8.3 Hz, 2H). ^{13}C NMR (101 MHz, DMSO-d_6) δ 172.60, 163.85, 151.96, 150.77, 130.45, 129.42, 129.29, 128.26, 126.88, 125.57, 124.05, 122.85, 117.16, 106.77, 83.64, 54.09, 53.07, 47.89, 27.84. HRMS m/z (EI-TOF): calcd for $C_{21}H_{21}N_3O_3$ (M^+): 363.1583; found 363.1585.

1-(2-((4,6-dimethoxypyrimidin-2-yl)oxy)benzyl)-2-methylindoline

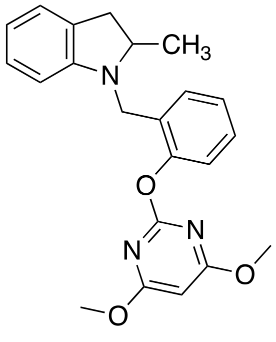

5g

The title compound **5g** was prepared according to general procedure D. **5g** was obtained as yellow liquid (222 mg, yield 98%). ^{1}H NMR (400 MHz, $CDCl_3$) δ 7.50 (d, *J* = 7.3 Hz, 1H), 7.29 (t, *J* = 7.4 Hz, 1H), 7.18 (t, *J* = 7.5 Hz, 1H), 7.15 – 7.11 (m, 1H), 7.02 (d, *J* = 7.1 Hz, 1H), 6.93 (t, *J* = 7.6 Hz, 1H), 6.60 (t, *J* = 7.3 Hz, 1H), 6.23 (d, *J* = 7.8 Hz, 1H), 5.76 (s, 1H), 4.23 (s, 2H), 3.81 (s, 6H), 3.76–3.69 (m, 1H), 3.13 (dd, *J* = 15.5, 8.6 Hz, 1H), 2.66 (dd, *J* = 15.5, 9.4 Hz, 1H), 1.26 (d, *J* = 6.1 Hz, 3H). ^{13}C NMR (101 MHz, $CDCl_3$) δ 173.07, 164.25, 152.70, 150.58, 131.78, 128.87, 128.51, 127.71, 127.33, 125.72, 124.10, 122.50, 117.45, 106.91, 84.71, 61.23, 54.26, 46.47, 37.48, 19.86. HRMS m/z (EI-TOF): calcd for $C_{22}H_{23}N_3O_3$ (M^+): 377.1739; found 377.1737.

3.5 General procedure E for the synthesis of products 5j, 5q, 5r, 8c and 8d

Example for the synthesis of **5j**:

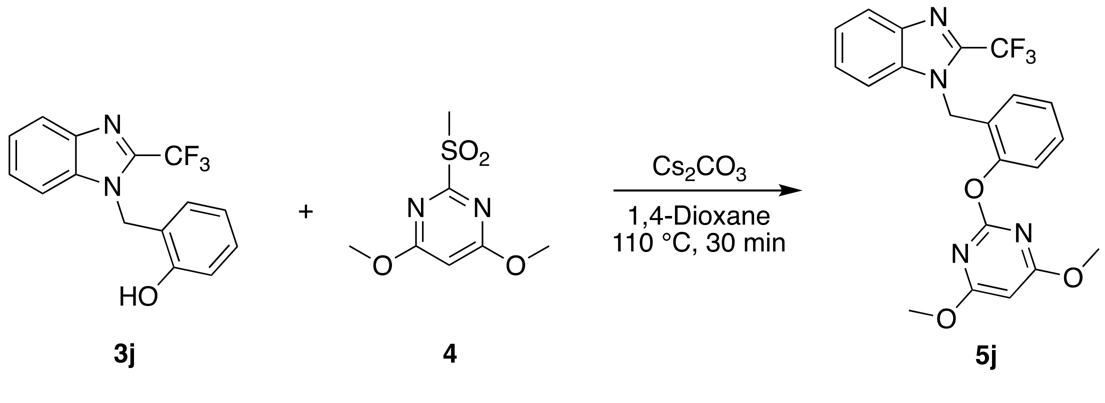

General procedure E: To a 50 mL round-bottom flask, were added **3j** (0.6 mmol), **4** (0.72 mmol), Cs_2CO_3 (0.9 mmol) and 1,4-dioxane (10 mL). The mixture was heated at 110 °C for 30 min until complete consumption of **3j**, as detected by TLC. Afterwards, the reaction mixture was quenched with brine, the aqueous layer was extracted with ethyl acetate (3×10 mL), and combined organic extracts were dried over Na_2SO_4. After filtration and evaporation of the solvents under vacuum, the crude product was purified by thin-layer chromatography on silica gel with petroleum ether - ethyl acetate

(V/V=10/1) as the developing agent to afford the desired product **5j**.

Analytical data for the products **5j**, **5q**, **5r**, **8c** and **8d**:

1-(2-((4,6-dimethoxypyrimidin-2-yl)oxy)benzyl)-2-(trifluoromethyl)-1*H*-benzo[*d*]imidazole

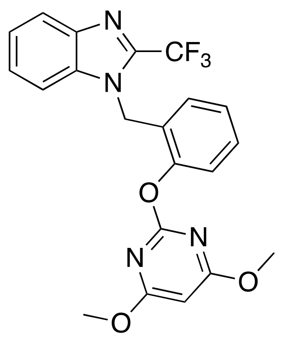

5j

The title compound **5j** was prepared according to general procedure E. **5j** was obtained as colorless liquid (185 mg, yield 72%). ^{1}H NMR (400 MHz, $CDCl_3$) δ 7.88 (dd, *J* = 6.9, 1.7 Hz, 1H), 7.37 – 7.28 (m, 4H), 7.20 (dd, *J* = 8.1, 0.9 Hz, 1H), 7.11 – 7.07 (m, 1H), 6.65 (d, *J* = 7.5 Hz, 1H), 5.80 (s, 1H), 5.56 (s, 2H), 3.82 (s, 6H). ^{13}C NMR (101 MHz, $CDCl_3$) δ 173.12, 163.80, 150.20, 141.18 (q, *J* = 38.7 Hz), 141.15, 135.56, 129.24, 127.70, 126.72, 126.16, 125.63, 123.95, 122.92, 121.71, 119.19 (q, *J* = 271.7 Hz), 111.36, 85.24, 54.34, 43.89. HRMS m/z (EI-TOF): calcd for $C_{21}H_{17}N_4O_3F_3$ (M^+): 430.1253; found 430.1254.

9-(2-((4,6-dimethoxypyrimidin-2-yl)oxy)benzyl)-2,3,4,9-tetrahydro-1*H*-carbazole

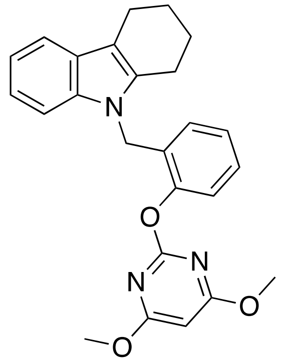

5q

The title compound **5q** was prepared according to general procedure E. **5q** was obtained as white solid (164 mg, yield 66%; mp: 143.8-144.5 °C). ^{1}H NMR (400 MHz, $CDCl_3$) δ 7.50 – 7.44 (m, 1H), 7.24 (d, *J* = 7.8 Hz, 1H), 7.15 – 7.11 (m, 2H), 7.07 – 7.02 (m, 3H), 6.57 (d, *J* = 7.4 Hz, 1H), 5.79 (s, 1H), 5.23 (s, 2H), 3.79 (s, 6H), 2.72 (t, *J* = 5.7 Hz, 2H), 2.60 (t, *J* = 5.8 Hz, 2H), 1.91 – 1.81 (m, 4H). ^{13}C NMR (101 MHz, $CDCl_3$) δ 173.08, 164.07, 150.23, 136.70, 135.78, 130.81, 128.22, 127.62, 127.39,

125.99, 122.56, 120.75, 118.94, 117.83, 109.95, 109.16, 84.91, 54.30, 41.86, 23.33, 23.27, 22.08, 21.21. HRMS m/z (EI-TOF): calcd for $C_{25}H_{25}N_3O_3$ (M^+): 415.1896; found 415.1890.

6-chloro-9-(2-((4,6-dimethoxypyrimidin-2-yl)oxy)benzyl)-2,3,4,9-tetrahydro-1*H*-carbazole

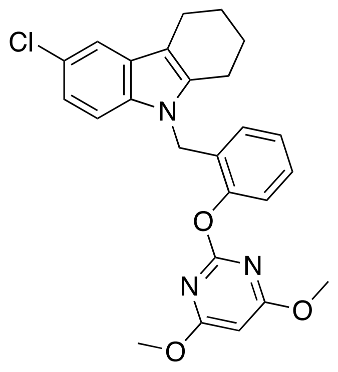

5r

The title compound **5r** was prepared according to general procedure E. **5r** was obtained as white solid (240 mg, yield 89%; mp: 142.5-143.4 °C). ^{1}H NMR (400 MHz, $CDCl_3$) δ 7.39 (d, J = 1.9 Hz, 1H), 7.30 – 7.24 (m, 1H), 7.13 (dd, J = 8.1, 1.0 Hz, 1H), 7.09 – 7.02 (m, 2H), 6.98 (dd, J = 8.6, 2.0 Hz, 1H), 6.60 (d, J = 7.5 Hz, 1H), 5.76 (s, 1H), 5.20 (s, 2H), 3.76 (s, 6H), 2.66 – 2.57 (m, 4H), 1.89 – 1.79 (m, 4H). ^{13}C NMR (101 MHz, $CDCl_3$) δ 173.03, 163.95, 150.33, 137.38, 135.07, 130.29, 128.66, 128.50, 127.48, 126.00, 124.66, 122.80, 120.78, 117.41, 110.19, 109.73, 84.83, 54.28, 42.25, 23.16, 23.07, 22.16, 21.03. HRMS m/z (EI-TOF): calcd for $C_{25}H_{24}N_3O_3Cl$ (M^+): 449.1506; found 449.1506.

3-(2-((4,6-dimethoxypyrimidin-2-yl)oxy)benzyl)-1-methyl-1*H*-indole

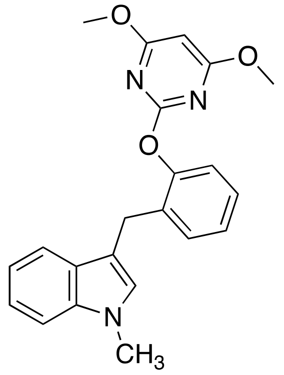

8c

The title compound **8c** was prepared according to general procedure E. **8c** was obtained as yellow liquid (223 mg, yield 99%). ^{1}H NMR (400 MHz, $CDCl_3$) δ 7.47 (d, J = 7.9 Hz, 1H), 7.30 (d, J = 7.6 Hz, 1H), 7.25 – 7.20 (m, 2H), 7.19 – 7.09 (m, 3H), 7.03 – 6.98 (m, 1H), 6.74 (s, 1H), 5.68 (s, 1H), 4.07 (s, 2H), 3.70 (s, 6H), 3.65 (s, 3H). ^{13}C NMR (101 MHz, $CDCl_3$) δ 172.79, 164.41, 151.18, 137.07,

133.87, 130.49, 128.01, 127.69, 127.02, 125.58, 122.84, 121.47, 119.46, 118.71, 112.67, 109.08, 84.16, 54.18, 32.59, 26.15. HRMS m/z (EI-TOF): calcd for $C_{22}H_{21}N_3O_3$ (M^+): 375.1583; found 375.1583.

3-(2-((4,6-dimethoxypyrimidin-2-yl)oxy)benzyl)-1,2-dimethyl-1*H*-indole

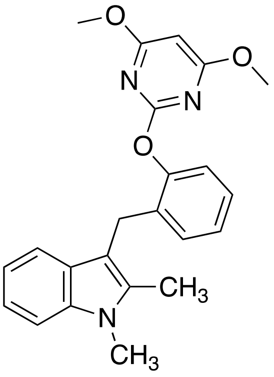

8d

The title compound **8d** was prepared according to general procedure E. **8d** was obtained as yellow solid (212 mg, yield 91%; mp: 93.2-94.7 °C). ^{1}H NMR (400 MHz, $CDCl_3$) δ 7.34 (d, *J* = 7.8 Hz, 1H), 7.22 – 7.16 (m, 2H), 7.13 – 7.04 (m, 4H), 6.96 (t, *J* = 7.4 Hz, 1H), 5.71 (s, 1H), 4.04 (s, 2H), 3.72 (s, 6H), 3.60 (s, 3H), 2.30 (s, 3H). ^{13}C NMR (101 MHz, $CDCl_3$) δ 172.81, 164.35, 151.15, 136.67, 133.95, 133.93, 130.00, 128.20, 126.79, 125.49, 122.59, 120.54, 118.77, 118.54, 108.46, 108.32, 84.26, 54.17, 29.56, 25.12, 10.48. HRMS m/z (EI-TOF): calcd for $C_{23}H_{23}N_3O_3$ (M^+): 389.1739; found 389.1737.

3.6 General procedure F for the synthesis of products 8g-8j and 9g-9j

Example for the synthesis of **8g** and **9g**:

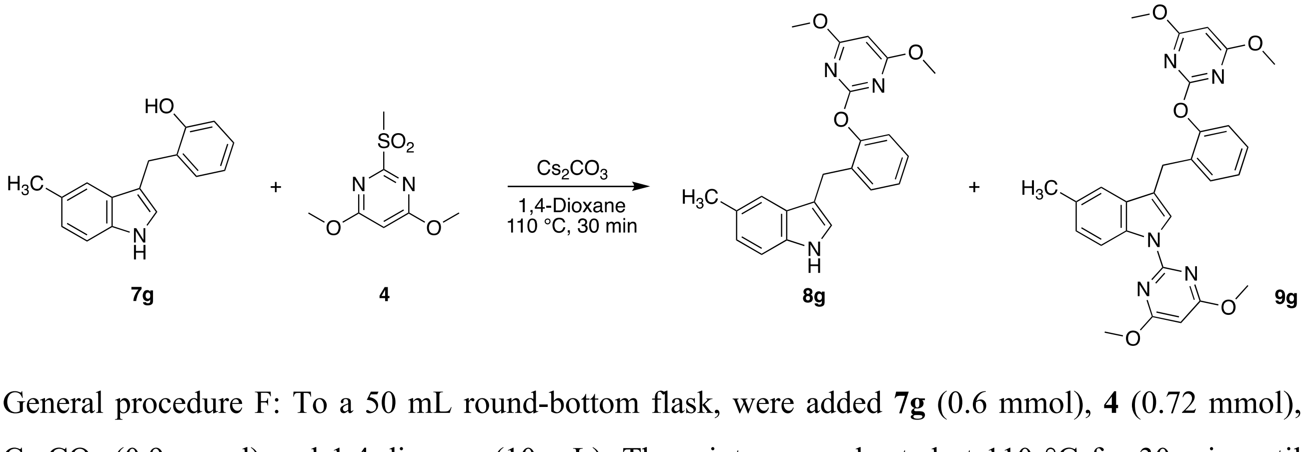

General procedure F: To a 50 mL round-bottom flask, were added **7g** (0.6 mmol), **4** (0.72 mmol), Cs_2CO_3 (0.9 mmol) and 1,4-dioxane (10 mL). The mixture was heated at 110 °C for 30 min until complete consumption of **7g**, as detected by TLC. Afterwards, the reaction mixture was quenched with brine, the aqueous layer was extracted with ethyl acetate (3×10 mL), and combined organic extracts

were dried over Na_2SO_4. After filtration and evaporation of the solvents under vacuum, the crude product was separated and purified by thin-layer chromatography on silica gel with petroleum ether - ethyl acetate (V/V=5/1) as the developing agent to afford the desired products **8g** and **9g**.

Analytical data for the products **8g-8j** and **9g-9j**:

3-(2-((4,6-dimethoxypyrimidin-2-yl)oxy)benzyl)-5-methyl-1*H*-indole

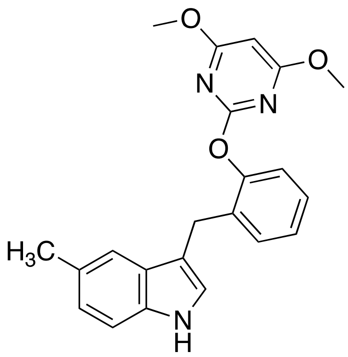

8g

The title compound **8g** was prepared according to general procedure F. **8g** was obtained as yellow liquid (165 mg, yield 73%). ^{1}H NMR (400 MHz, $CDCl_3$) δ 7.82 (s, 1H), 7.25 – 7.21 (m, 3H), 7.19 (s, 1H), 7.13 – 7.09 (m, 2H), 6.97 (d, *J* = 8.2 Hz, 1H), 6.90 (s, 1H), 5.71 (s, 1H), 4.04 (s, 2H), 3.75 (s, 6H), 2.38 (s, 3H). ^{13}C NMR (101 MHz, $CDCl_3$) δ 172.96, 164.46, 151.13, 134.75, 133.84, 130.32, 128.52, 127.89, 126.96, 125.56, 123.62, 123.11, 122.63, 118.92, 113.75, 110.75, 84.37, 54.23, 25.77, 21.58. HRMS m/z (EI-TOF): calcd for $C_{22}H_{21}N_3O_3$ (M^+): 375.1583; found 375.1581.

4-chloro-3-(2-((4,6-dimethoxypyrimidin-2-yl)oxy)benzyl)-1*H*-indole

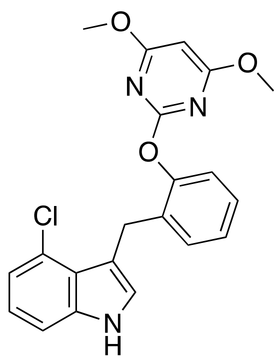

8h

The title compound **8h** was prepared according to general procedure F. **8h** was obtained as yellow liquid (156 mg, yield 66%). ^{1}H NMR (400 MHz, $CDCl_3$) δ 8.13 (s, 1H), 7.23 (t, *J* = 7.4 Hz, 2H), 7.18 – 7.08 (m, 3H), 7.06 – 7.00 (m, 2H), 6.84 (s, 1H), 5.72 (s, 1H), 4.33 (s, 2H), 3.79 (s, 6H). ^{13}C NMR

(101 MHz, $CDCl_3$) δ 172.97, 164.40, 151.02, 137.89, 134.48, 130.45, 126.90, 126.69, 125.51, 124.72, 124.56, 122.56, 122.52, 120.49, 114.65, 109.99, 84.45, 54.34, 26.91. HRMS m/z (EI-TOF): calcd for $C_{21}H_{18}N_3O_3Cl$ (M^+): 395.1037; found 395.1037.

5-chloro-3-(2-((4,6-dimethoxypyrimidin-2-yl)oxy)benzyl)-1*H*-indole

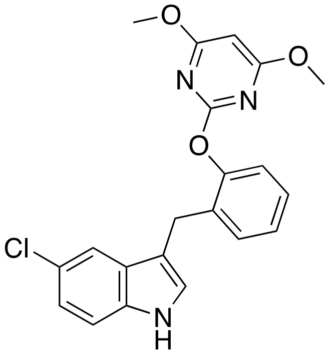

8i

The title compound **8i** was prepared according to general procedure F. **8i** was obtained as yellow liquid (136 mg, yield 57%). ^{1}H NMR (400 MHz, $CDCl_3$) δ 7.94 (s, 1H), 7.41 (d, *J* = 1.8 Hz, 1H), 7.26 – 7.18 (m, 3H), 7.16 – 7.06 (m, 3H), 6.95 (s, 1H), 5.69 (s, 1H), 4.02 (s, 2H), 3.74 (s, 6H). ^{13}C NMR (101 MHz, $CDCl_3$) δ 172.92, 164.36, 151.15, 134.70, 133.28, 130.25, 128.72, 127.26, 125.67, 125.10, 124.39, 122.92, 122.30, 118.85, 114.19, 112.14, 84.46, 54.39, 25.95. HRMS m/z (EI-TOF): calcd for $C_{21}H_{18}N_3O_3Cl$ (M^+): 395.1037; found 395.1039.

6-chloro-3-(2-((4,6-dimethoxypyrimidin-2-yl)oxy)benzyl)-1*H*-indole

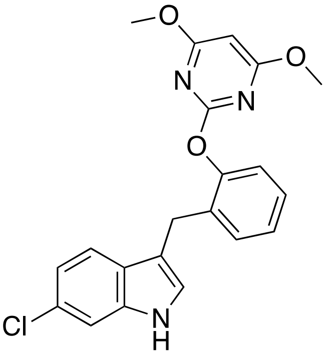

8j

The title compound **8j** was prepared according to general procedure F. **8j** was obtained as yellow liquid (161 mg, yield 68%). ^{1}H NMR (400 MHz, DMSO-d_6) δ 10.94 (s, 1H), 7.39 – 7.32 (m, 2H), 7.27 – 7.22 (m, 2H), 7.17 – 7.12 (m, 2H), 7.07 (d, *J* = 2.2 Hz, 1H), 6.90 (dd, *J* = 8.4, 1.9 Hz, 1H), 5.93 (s, 1H), 3.90 (s, 2H), 3.70 (s, 6H). ^{13}C NMR (101 MHz, DMSO-d_6) δ 172.67, 163.84, 150.51, 136.58,

133.30, 130.18, 127.13, 125.72, 125.66, 125.46, 124.70, 122.53, 119.83, 118.50, 112.56, 110.92, 83.58, 54.13, 25.20. HRMS m/z (ESI-TOF): calcd for $C_{21}H_{18}ClN_3O_3$ $(M+H)^+$: 396.1109; found 396.1110.

1-(4,6-dimethoxypyrimidin-2-yl)-3-(2-((4,6-dimethoxypyrimidin-2-yl)oxy)benzyl)-5-methyl-1*H*-indole

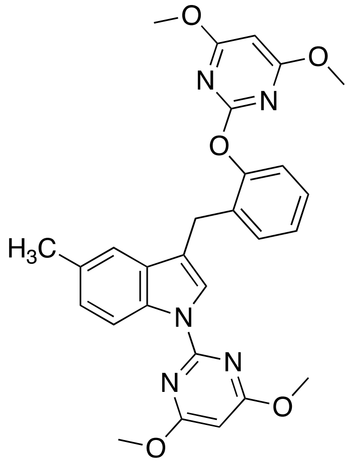

9g

The title compound **9g** was prepared according to general procedure F. **9g** was obtained as colorless liquid (28 mg, yield 9%). ^{1}H NMR (400 MHz, $CDCl_3$) δ 8.53 (d, *J* = 8.5 Hz, 1H), 7.92 (s, 1H), 7.31 (d, *J* = 7.6 Hz, 1H), 7.25 – 7.21 (m, 1H), 7.18 (s, 1H), 7.13 (t, *J* = 7.6 Hz, 2H), 7.08 (d, *J* = 8.5 Hz, 1H), 5.80 (s, 1H), 5.60 (s, 1H), 4.07 (s, 2H), 4.04 (s, 6H), 3.67 (s, 6H), 2.37 (s, 3H). ^{13}C NMR (101 MHz, $CDCl_3$) δ 172.85, 171.99, 151.22, 134.04, 132.86, 131.49, 131.12, 130.33, 127.25, 125.63, 125.05, 124.75, 122.88, 119.41, 117.54, 115.83, 84.39, 84.12, 54.44, 54.15, 26.08, 21.48. HRMS m/z (EI-TOF): calcd for $C_{28}H_{27}N_5O_5$ (M^+): 513.2012; found 513.2014.

4-chloro-1-(4,6-dimethoxypyrimidin-2-yl)-3-(2-((4,6-dimethoxypyrimidin-2-yl)oxy)benzyl)-1*H*-indole

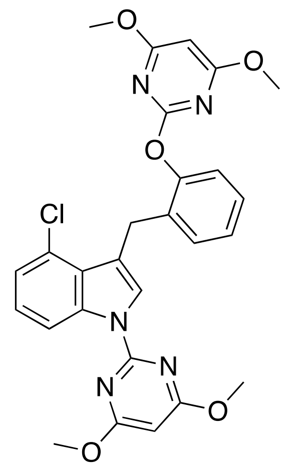

9h

The title compound **9h** was prepared according to general procedure F. **9h** was obtained as colorless liquid (38 mg, yield 12%). ^{1}H NMR (400 MHz, $CDCl_3$) δ 8.69 (d, J = 8.1 Hz, 1H), 7.92 (s, 1H), 7.25 – 7.20 (m, 1H), 7.20 – 7.07 (m, 5H), 5.84 (s, 1H), 5.68 (s, 1H), 4.35 (s, 2H), 4.02 (s, 6H), 3.75 (s, 6H). ^{13}C NMR (101 MHz, $CDCl_3$) δ 172.93, 171.99, 164.40, 155.78, 151.02, 137.29, 133.81, 130.12, 128.02, 127.00, 126.76, 126.55, 125.45, 124.23, 123.03, 122.59, 117.54, 114.79, 85.22, 84.46, 54.55, 54.28, 27.14. HRMS m/z (EI-TOF): calcd for $C_{27}H_{24}N_5O_5Cl$ (M^+): 533.1466; found 533.1467.

5-chloro-1-(4,6-dimethoxypyrimidin-2-yl)-3-(2-((4,6-dimethoxypyrimidin-2-yl)oxy)benzyl)-1*H*-indole

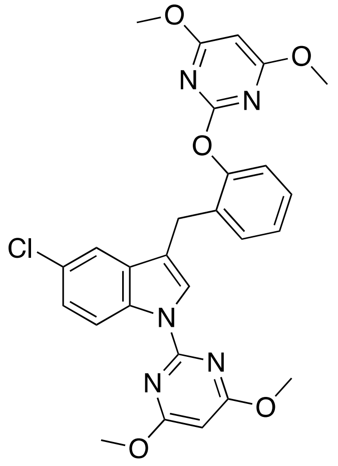

9i

The title compound **9i** was prepared according to general procedure F. **9i** was obtained as colorless liquid (50 mg, yield 16%). ^{1}H NMR (400 MHz, $CDCl_3$) δ 8.57 (d, J = 8.9 Hz, 1H), 7.98 (s, 1H), 7.35 (d, J = 2.0 Hz, 1H), 7.31 (d, J = 7.5 Hz, 1H), 7.28 – 7.23 (m, 1H), 7.22 – 7.15 (m, 2H), 7.11 (d, J = 8.0 Hz, 1H), 5.83 (s, 1H), 5.56 (s, 1H), 4.06 (s, 2H), 4.03 (s, 6H), 3.65 (s, 6H). ^{13}C NMR (101 MHz,

CDCl$_3$) δ 172.81, 172.03, 164.30, 155.85, 151.27, 134.08, 132.42, 132.32, 130.29, 127.54, 127.39, 125.95, 125.75, 123.74, 123.18, 119.15, 117.28, 117.14, 84.94, 84.13, 54.54, 54.16, 26.31. HRMS m/z (EI-TOF): calcd for $C_{27}H_{24}N_5O_5Cl$ (M^+): 533.1466; found 533.1467.

6-chloro-1-(4,6-dimethoxypyrimidin-2-yl)-3-(2-((4,6-dimethoxypyrimidin-2-yl)oxy)benzyl)-1*H*-indole

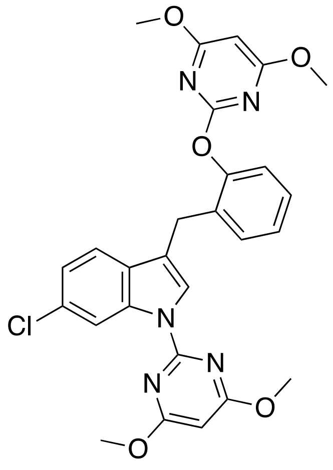

9j

The title compound **9j** was prepared according to general procedure F. **9j** was obtained as white solid (48 mg, yield 15%; mp: 170.3-171.5 °C). ^{1}H NMR (400 MHz, CDCl$_3$) δ 8.71 (d, *J* = 1.8 Hz, 1H), 7.93 (s, 1H), 7.33 – 7.29 (m, 2H), 7.23 (dd, *J* = 7.5, 1.6 Hz, 1H), 7.17 – 7.06 (m, 3H), 5.83 (s, 1H), 5.55 (s, 1H), 4.07 (s, 2H), 4.05 (s, 6H), 3.63 (s, 6H). ^{13}C NMR (101 MHz, CDCl$_3$) δ 172.83, 172.01, 164.32, 155.80, 151.26, 136.00, 132.46, 130.34, 129.67, 129.41, 127.50, 125.67, 125.10, 123.14, 122.14, 120.36, 117.74, 116.51, 85.02, 84.03, 54.56, 54.11, 26.39. HRMS m/z (ESI-TOF): calcd for $C_{27}H_{24}ClN_5O_5$ $(M+H)^+$: 534.1539; found 534.1542.

4. Biological Testing

The herbicidal activity was evaluated in the Green Pesticide Collaborative Innovation Center of Zhejiang A&F University (Hangzhou, China). All test compounds (**5a-5s**, **8a-8k** and **9g-9j**) were dissolved in DMSO to 1.0% and then diluted with Tween-80 (0.1%) to the appropriate concentrations before use. The post-emergence herbicidal activity of 28 compounds (**5a-5c**, **5e**, **5h-5q**, **5s**, **8a-8b**, **8e-8k** and **9g-9j**) was evaluated against three dicotyledonous weeds, *Abutilon theophrasti* (AT), *Amaranthus retroflexus* (AR), and *Cassia tora* (CT), and two monocotyledonous weeds, *Echinochloa crusgalli* (EC), and *Digitaria sanguinalis* (DS). Flowerpots with an inner diameter of 8.0 cm were filled with soil. The seeds were sown in the pot and covered with soil to a thickness of 0.2～0.5 cm and grown at 25±8 °C in a greenhouse. When the weeds had grown to about the two-leaf stage, they were treated by the solutions of synthesized compounds at the required concentrations (750 g ai/ha). Moreover, untreated seedlings were used as the control group, the solvent (DMSO + Tween-80) treated seedlings were used as the solvent control group, and oxyfluorfen (30 g ai/ha) was selected as the positive control group. The post-emergence herbicidal activity of the compounds was visually evaluated after 21 days of treatment, and the results of the herbicidal activity are shown in Table S2, with two replicates per treatment and averaged figures.

Table S2 Herbicidal activity of 28 synthesized compounds against five kinds of weeds[a]

	Post-emergence, 750 g ai/ha				
compound	AT	EC	AR	DS	CT
**5a**	−	−	+	+++	+
**5b**	−	−	−	−	+
**5c**	−	−	−	+	−
**5e**	+	−	−	+++	+
**5h**	+	+	+	++	+
**5i**	−	−	−	−	++
**5j**	−	−	+	+++	+
**5k**	−	−	−	+	−
**5l**	−	−	−	−	−
**5m**	+	+	−	++	++
**5n**	−	−	−	+++	+
**5o**	−	−	+	+++	+
**5p**	−	−	−	++	+

**5q**	++++	+++	+++	+++	++++
**5s**	−	−	−	++	−
**8a**	−	−	−	−	−
**8b**	−	−	−	−	−
**8e**	+	−	−	+++	+
**8f**	−	−	−	−	−
**8g**	+	−	−	++	−
**8h**	+++	+	+++	+++	+
**8i**	+	−	−	+++	−
**8j**	−	−	−	−	−
**8k**	+	−	−	+++	−
**9g**	+	−	−	++	+
**9h**	−	−	−	++	−
**9i**	+	+	+	+++	−
**9j**	−	−	−	++	+
oxyfluorfen[b]	++++	++++	++++	++++	++

[a] AT for *Abutilon theophrasti*; EC for *Echinochloa crusgalli*; AR for *Amaranthus retroflexus*; DS for *Digitaria sanguinalis*; CT for *Cassia tora*. Rating system for the growth inhibition percentage: ++++, ≥80%; +++, 60−79%; ++, 50−59%; +, 30−49%; −, <30%. [b] 30 g ai/ha.

Furthermore, the post-emergence herbicidal activity of other 6 compounds (**5d**, **5f-5g**, **5r** and **8c-8d**) was evaluated against two dicotyledonous weeds, *Galium spurium* (GS), and *Sonchus oleraceus* (SO), and three monocotyledonous weeds, *Alopecurus aequalis* (AA), *Polypogon fugax* (PF), and *Bromus japonicus* (BJ). The seeds were grown at 15±8 °C in a greenhouse, and diflufenican (150 g ai/ha) was selected as the positive control. Other conditions were the same as above. Herbicidal activity was evaluated visually 22 days post treatment, and the results are shown in Table S3, two replicates per treatment.

Table S3 Herbicidal activity of 6 synthesized compounds against other five kinds of weeds[a]

	Post-emergence, 750 g ai/ha				
compound	AA	PF	GS	BJ	SO
**5d**	−	−	−	−	−
**5f**	−	−	−	+	−

**5g**	++	−	+	−	−
**5r**	+	−	−	−	−
**8c**	−	−	−	−	−
**8d**	−	−	−	−	−
diflufenican[b]	++++	+++	−	−	−

[a] AA for *Alopecurus aequalis*; PF for *Polypogon fugax*; GS for *Galium spurium*; BJ for *Bromus japonicus*; SO for *Sonchus oleraceus*. Rating system for the growth inhibition percentage: ++++, ≥80%; +++, 60−79%; ++, 50−59%; +, 30−49%; −, <30%. [b] 150 g ai/ha.

5. References

1. N. E. Sidorina and V. A. Osyanin, *Chem. Heterocyclic Com.*, 2007, **43**, 1065-1071.
2. V. A. Osyanin, E. S. Selezneva, Z. P. Belousova, L. F. Zarina, N. E. Krel and P. P. Purygin, *Pharm. Chem. J.*, 2003, **37**, 482-484.
3. J.-P. Meng, W.-W. Wang, Y.-L. Chen, S. Bera and J. Wu, *Org. Chem. Front.*, 2020, **7**, 267-272.

6. NMR spectra

5a (400 MHz, $CDCl_3$)

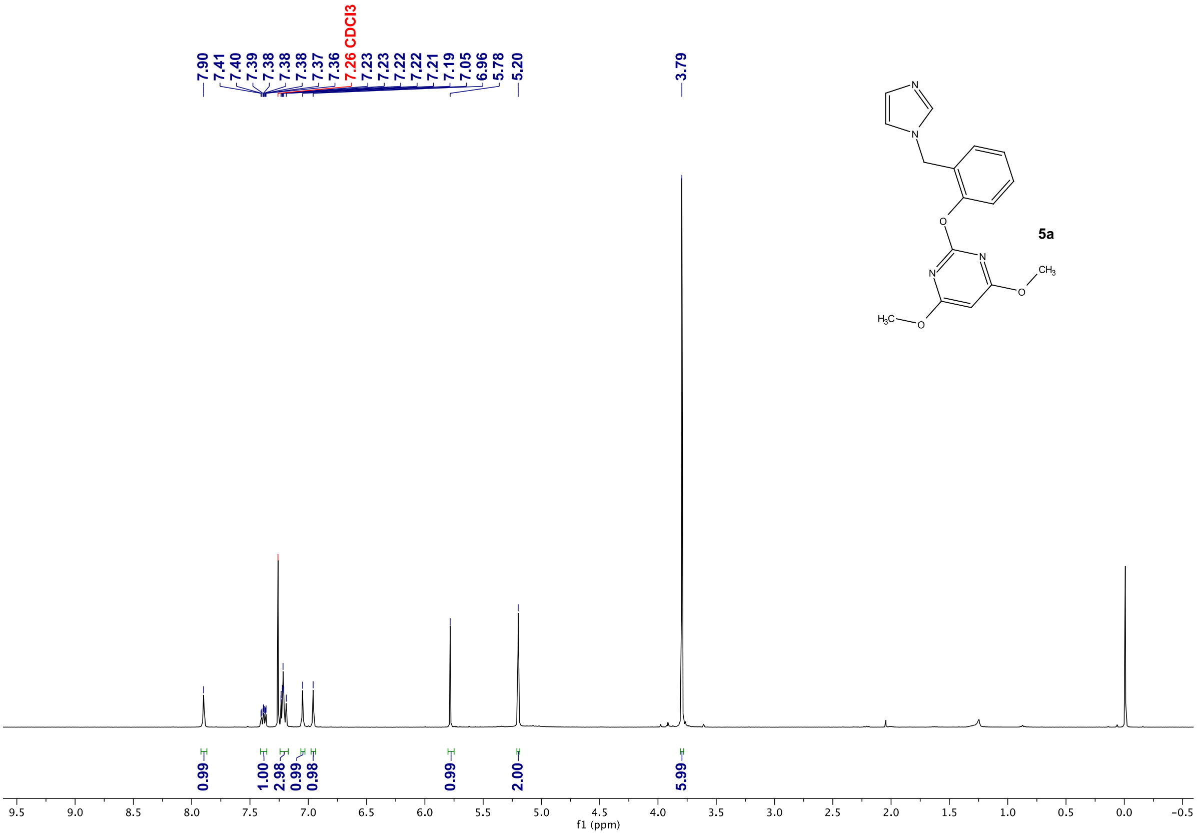

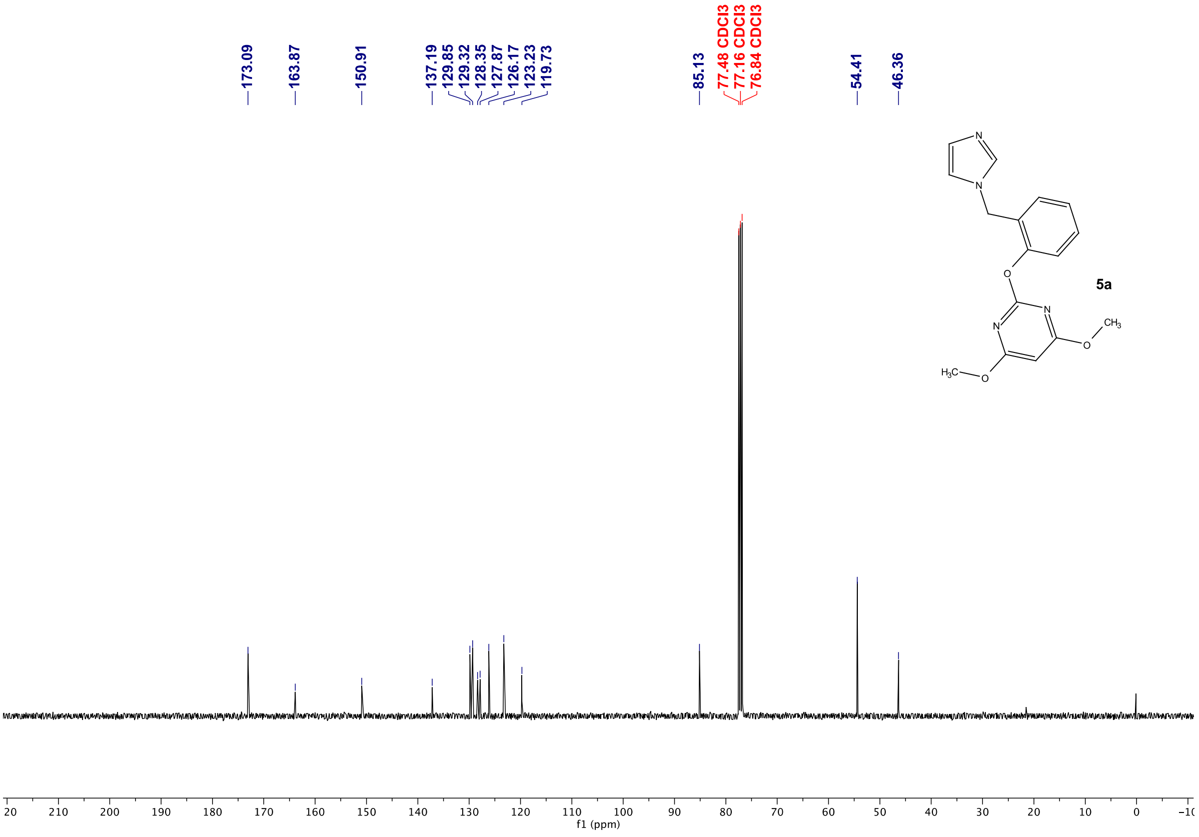

5b (400 MHz, $CDCl_3$)

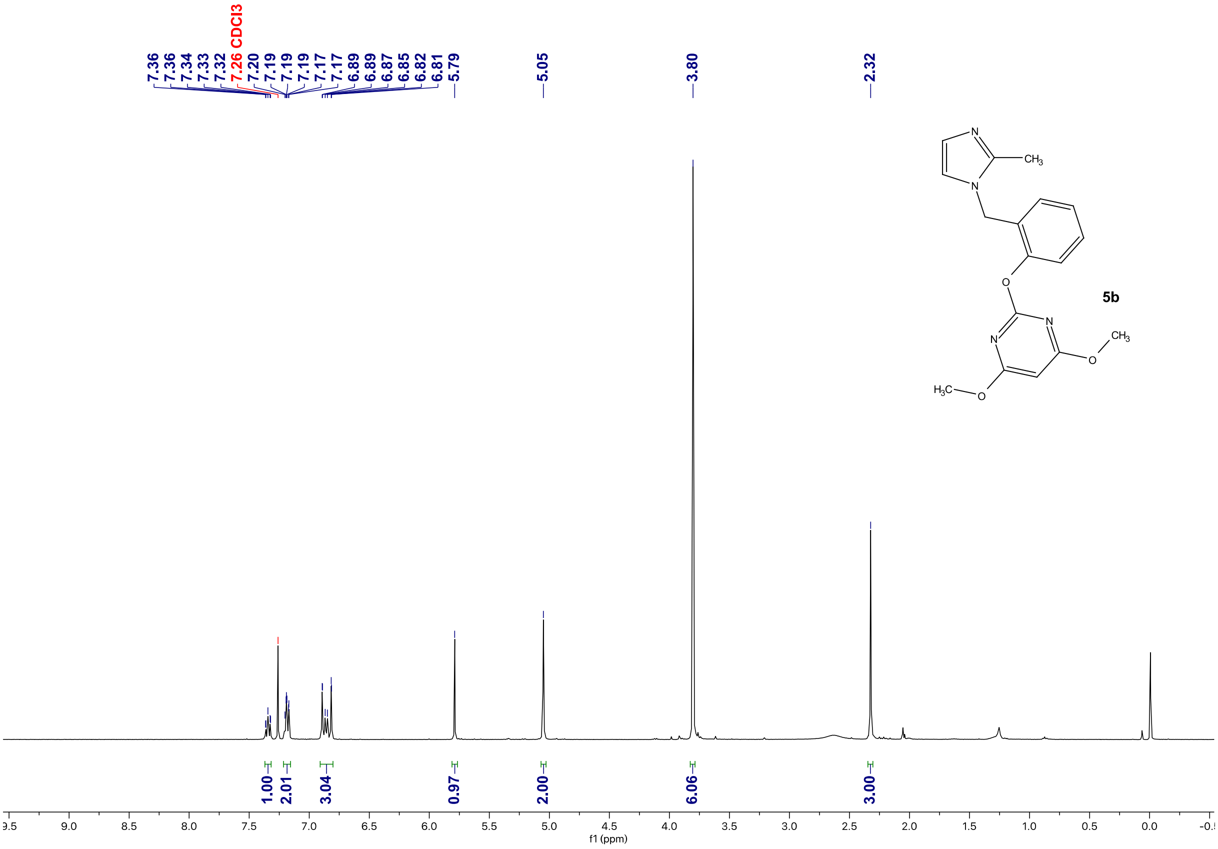

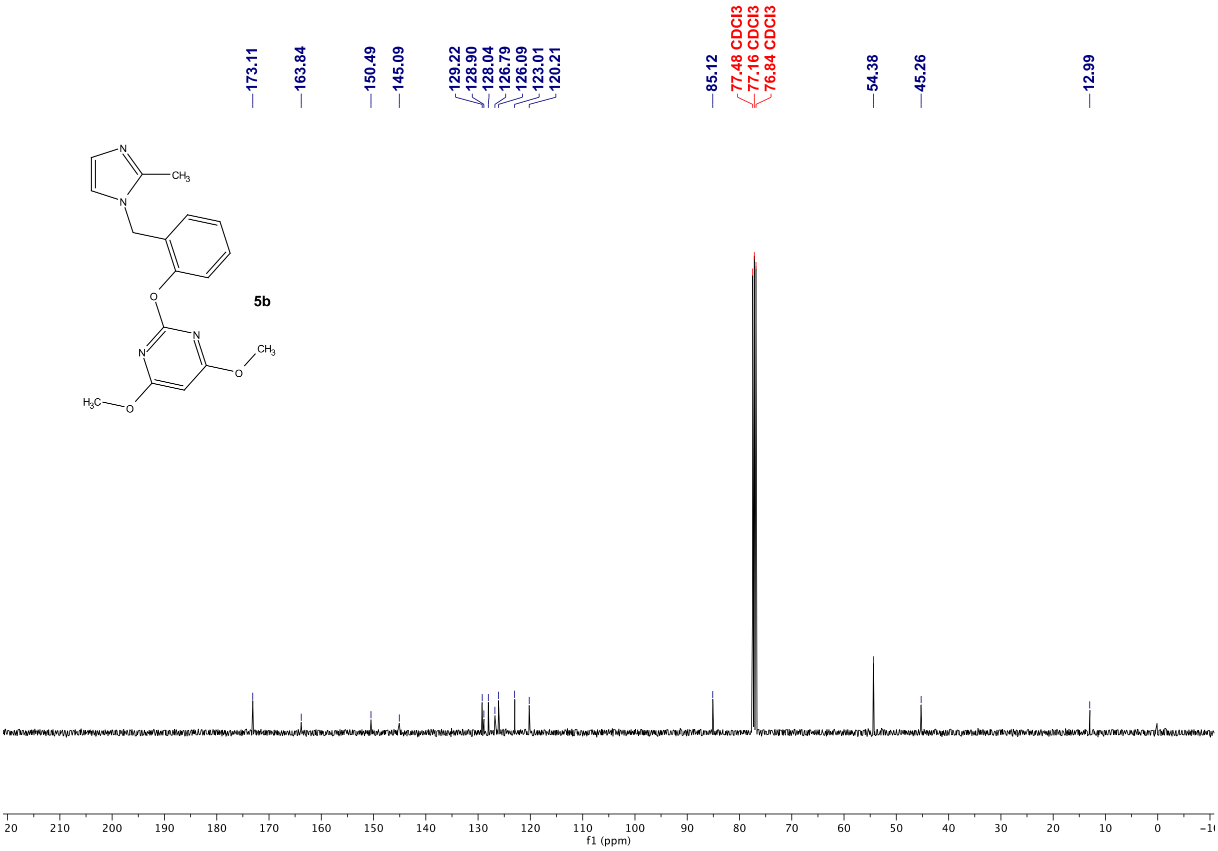

5c (400 MHz, $CDCl_3$)

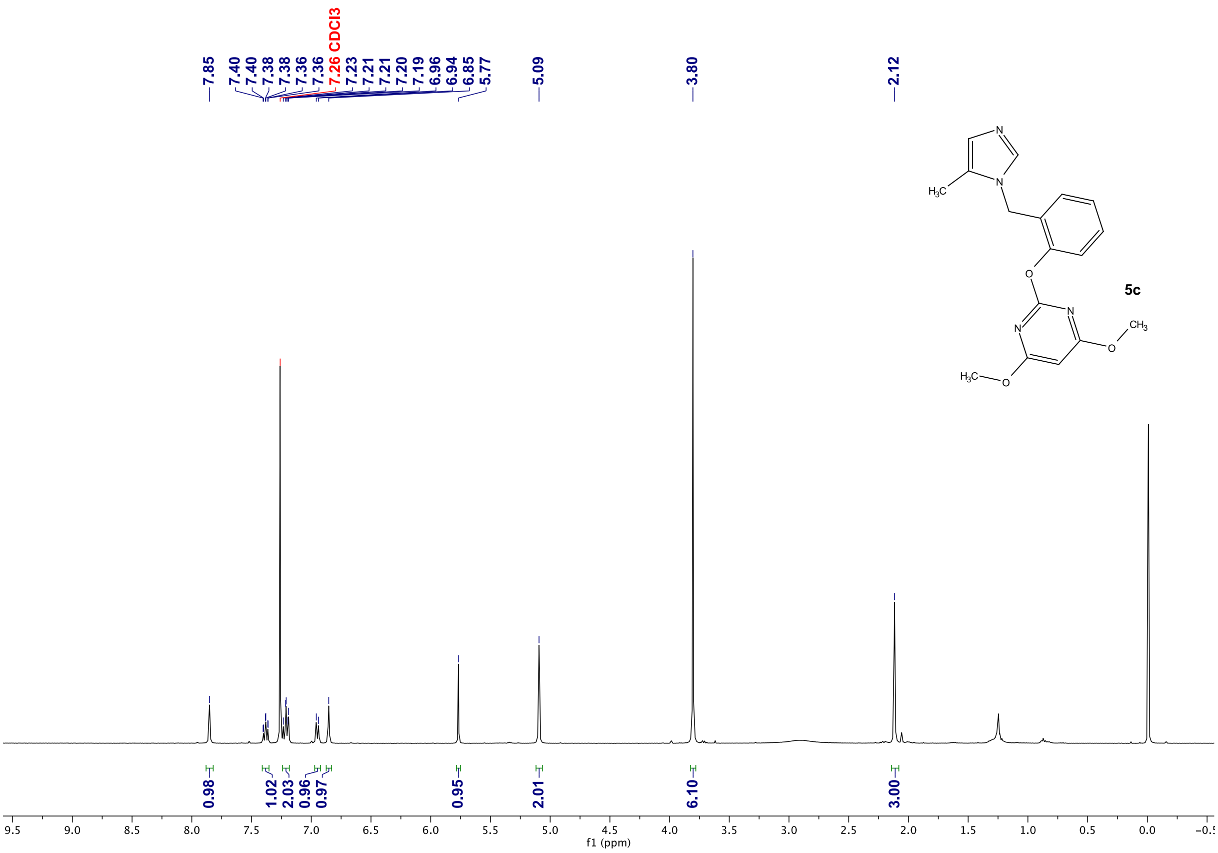

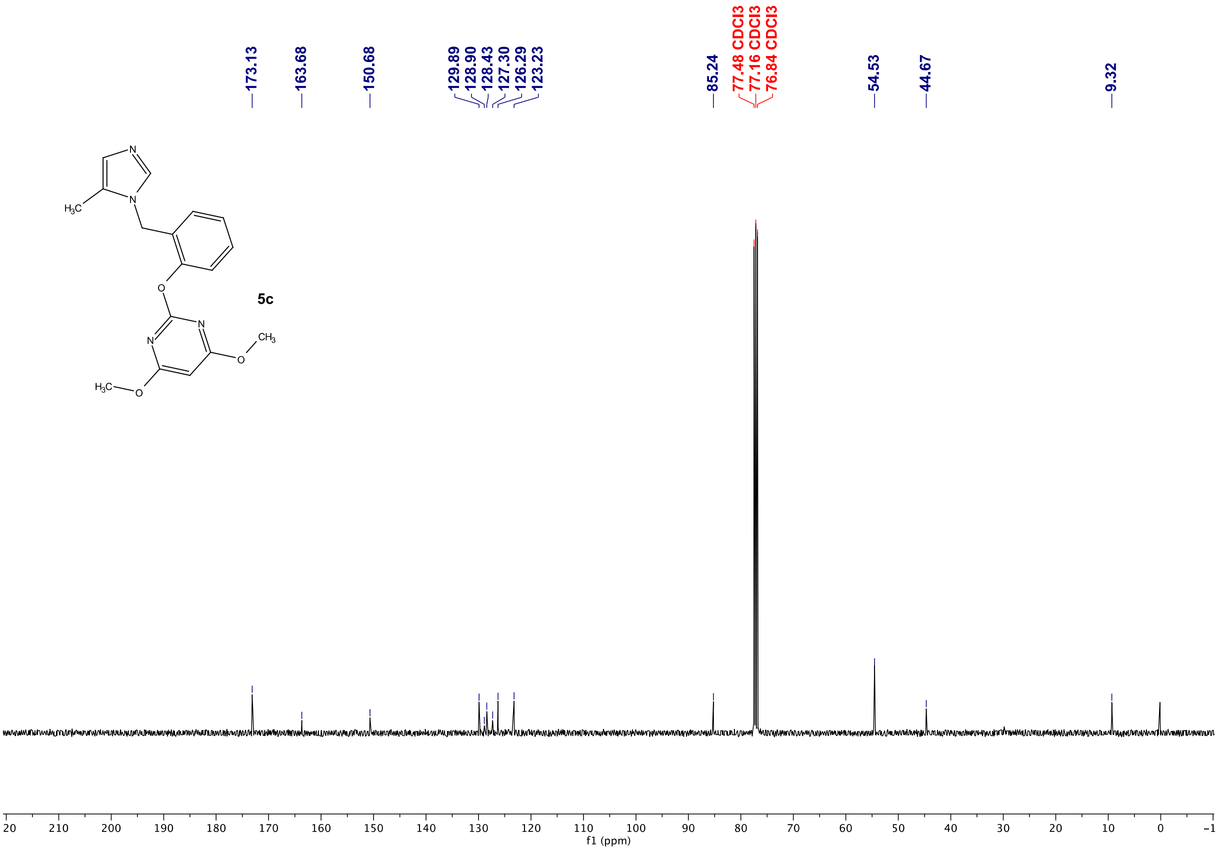

5d (400 MHz, $CDCl_3$)

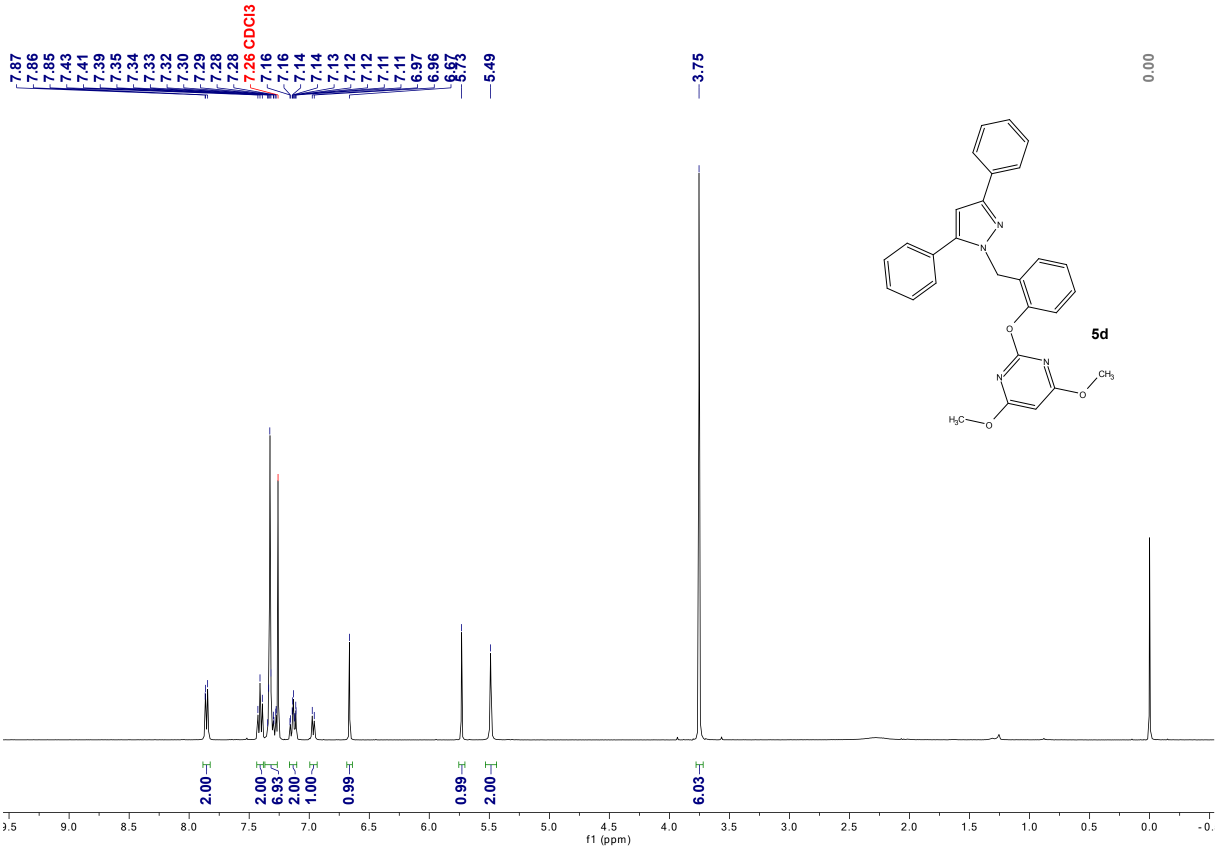

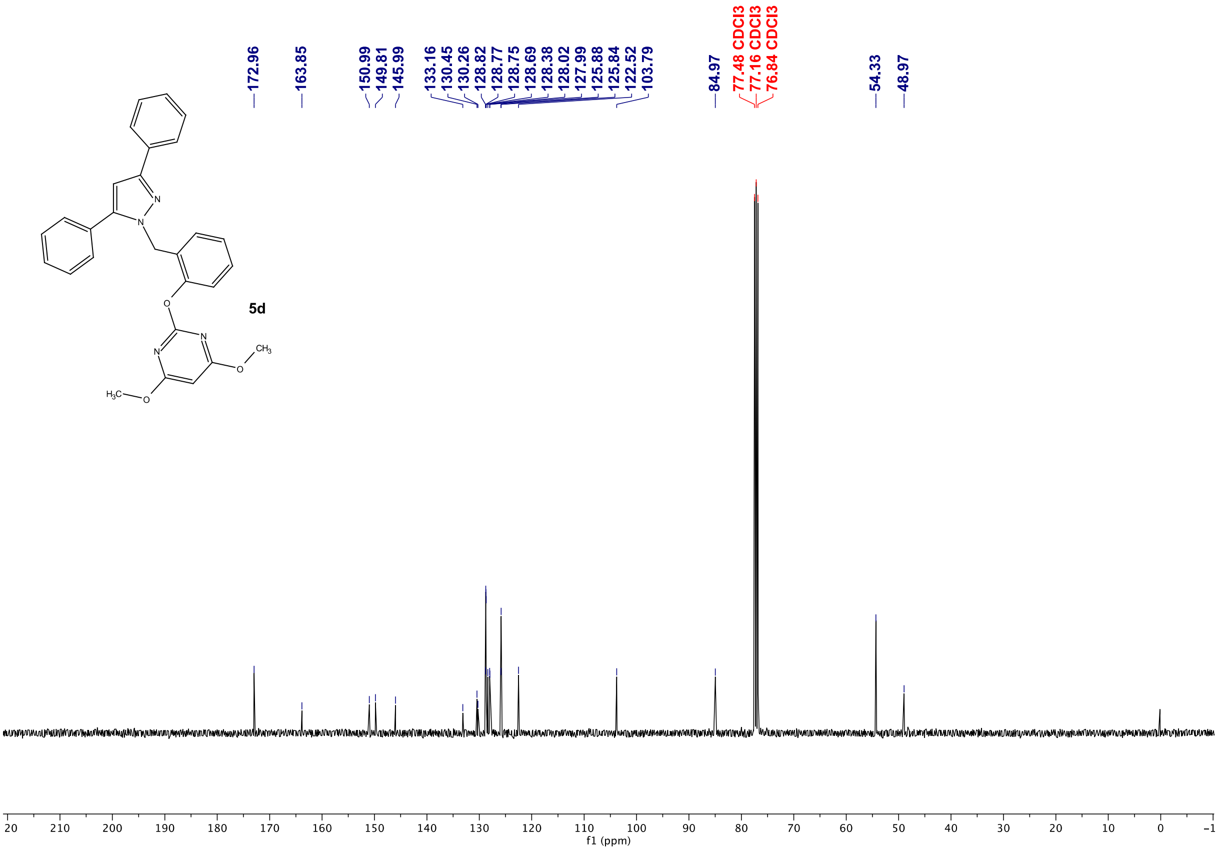

5e (400 MHz, $CDCl_3$)

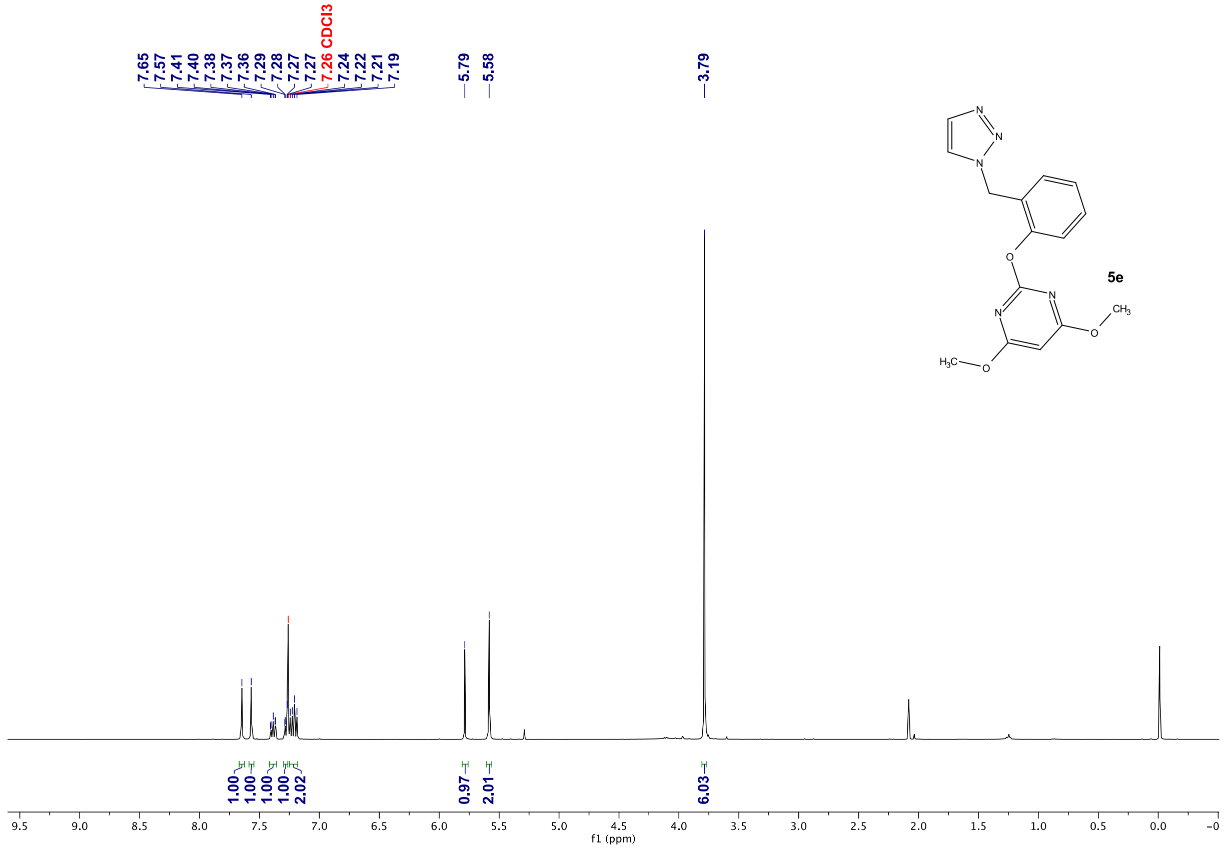

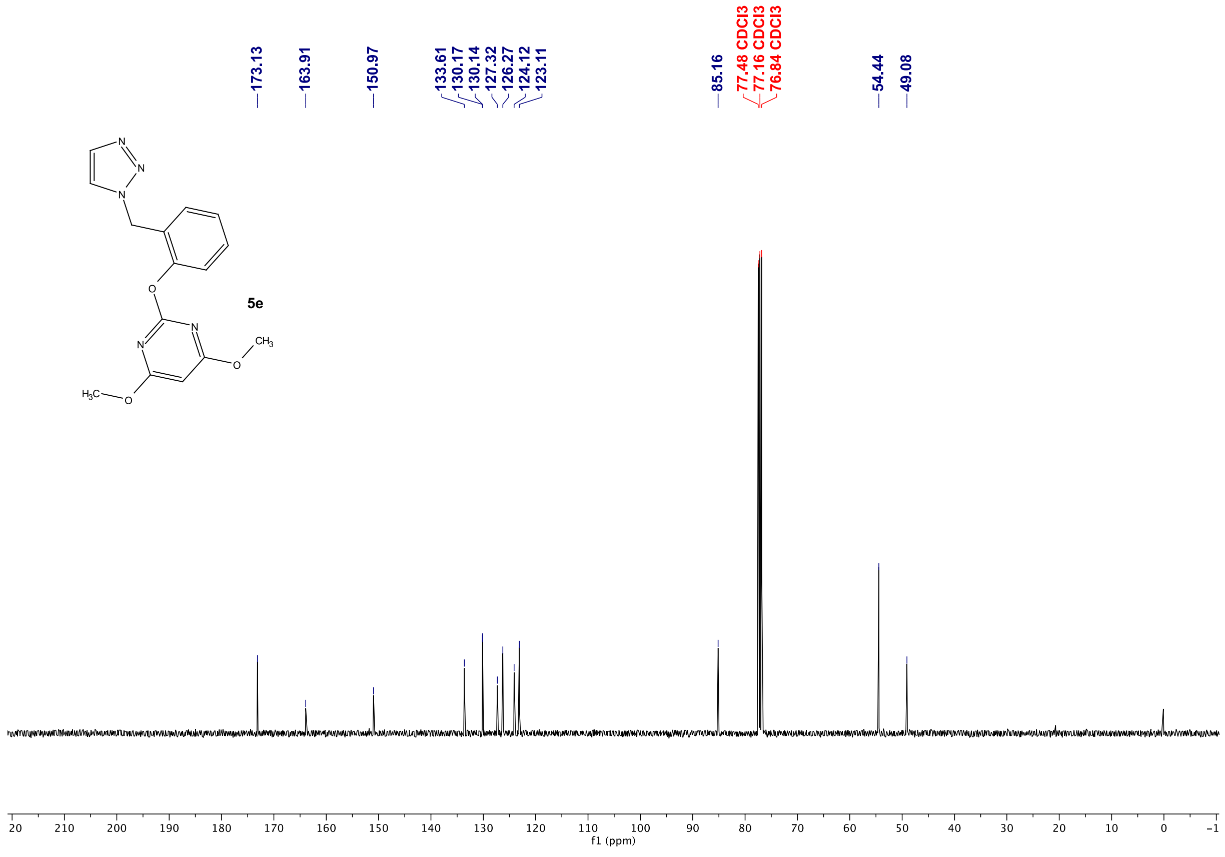

5f (400 MHz, $CDCl_3$)

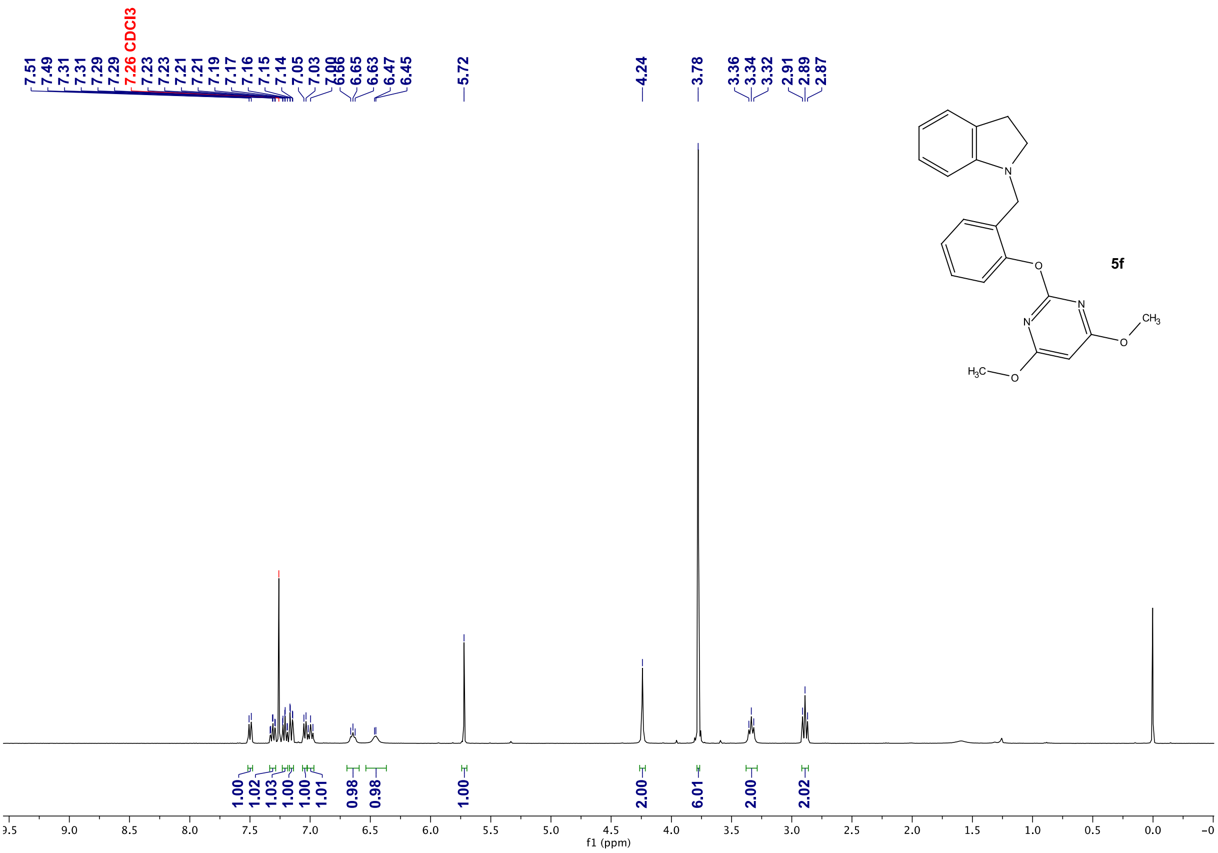

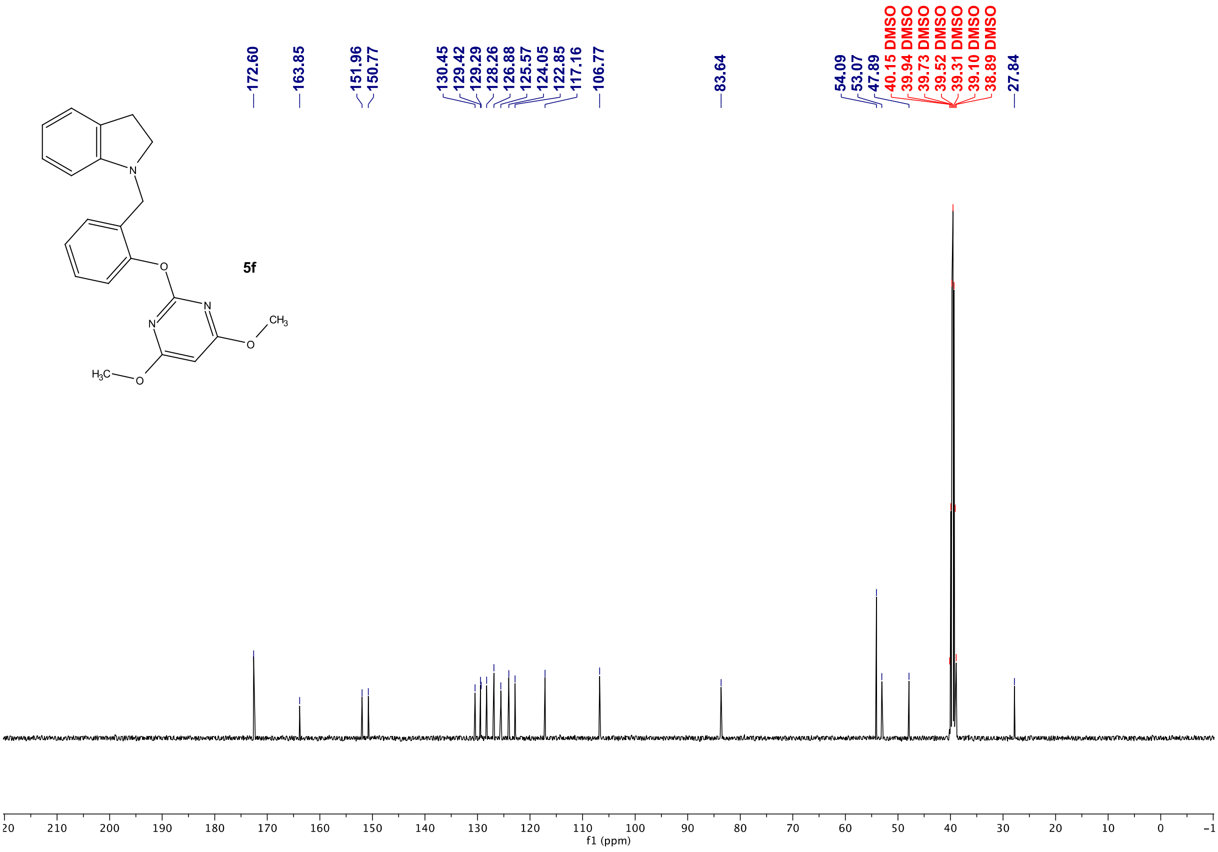

5g (400 MHz, $CDCl_3$)

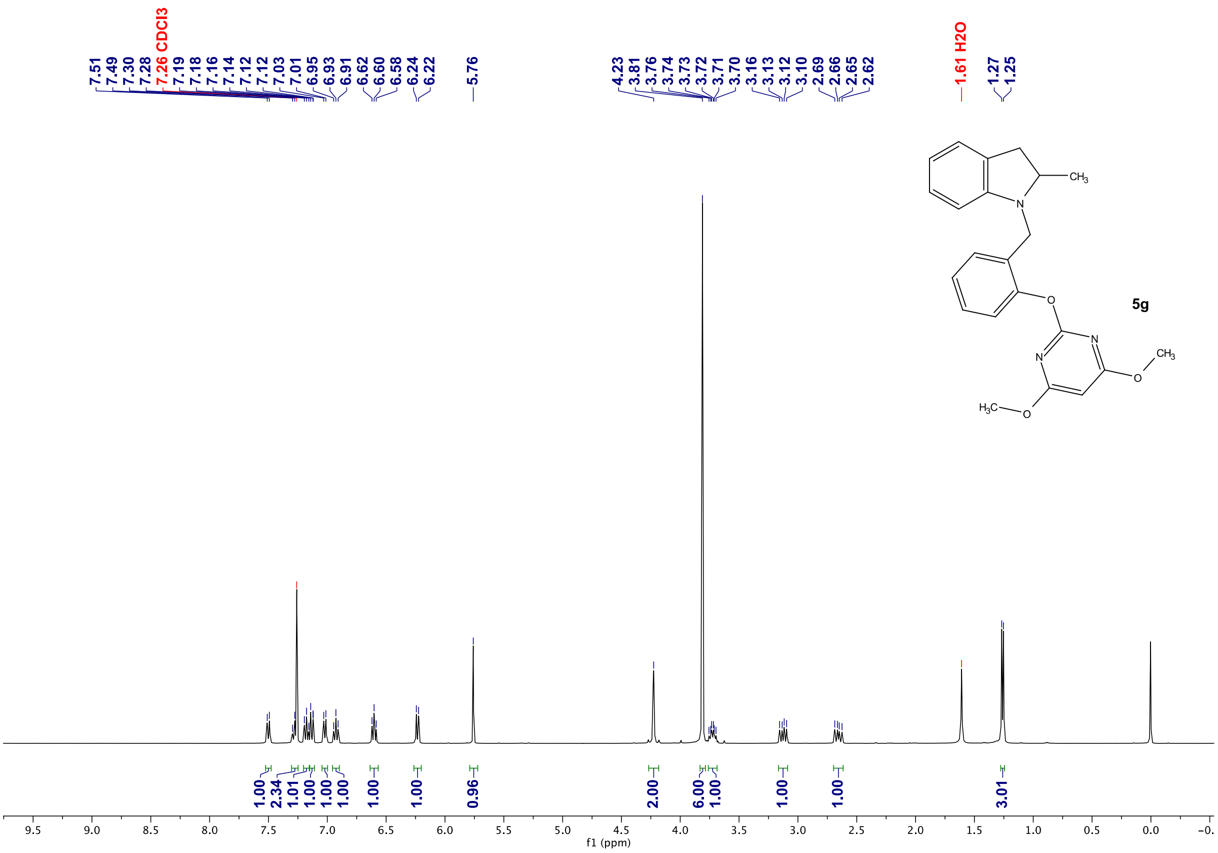

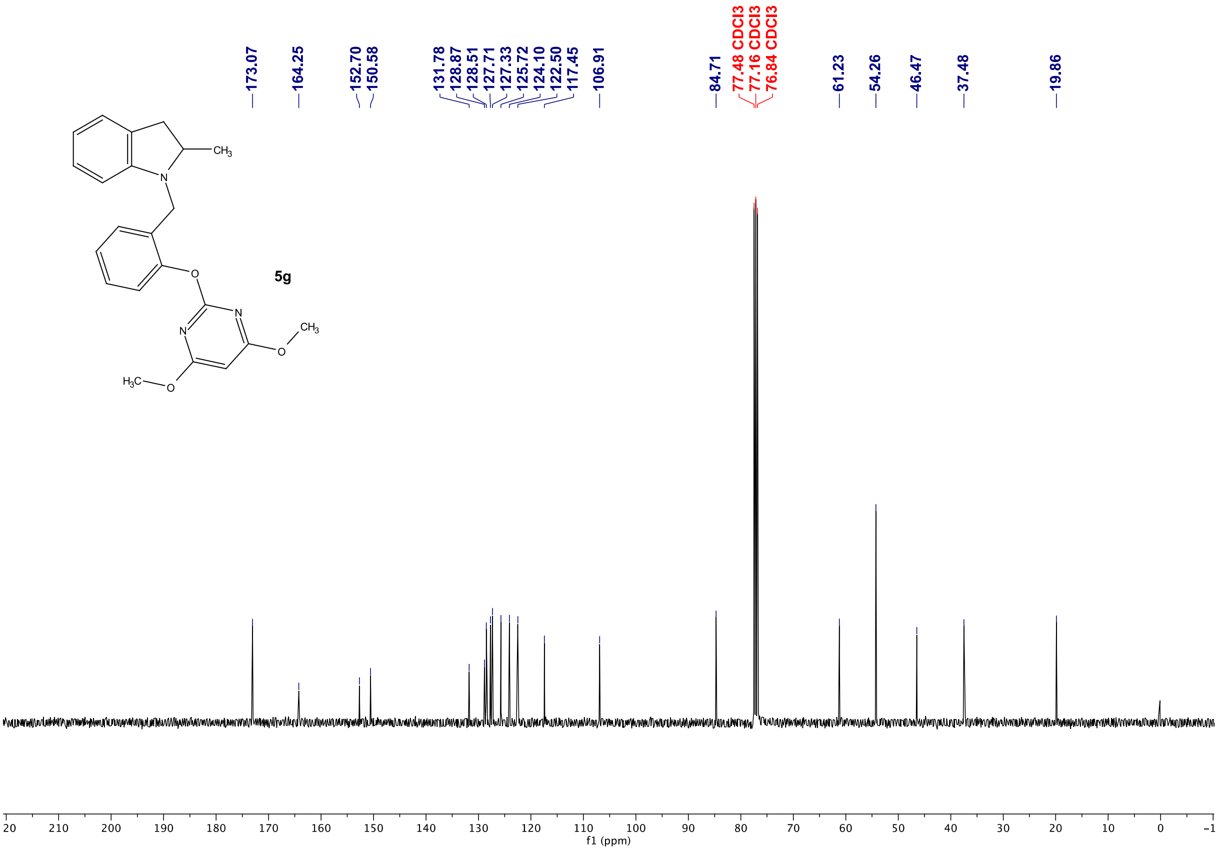

5h (400 MHz, $CDCl_3$)

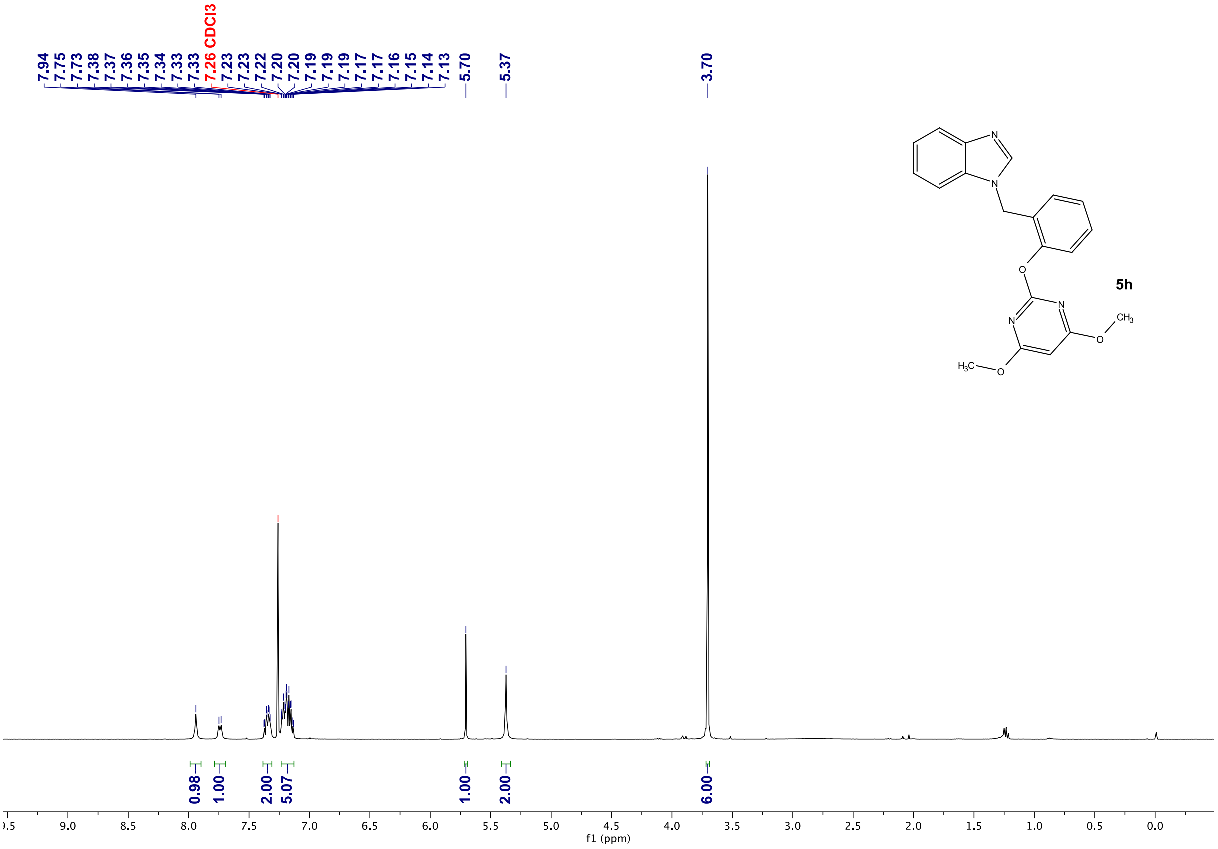

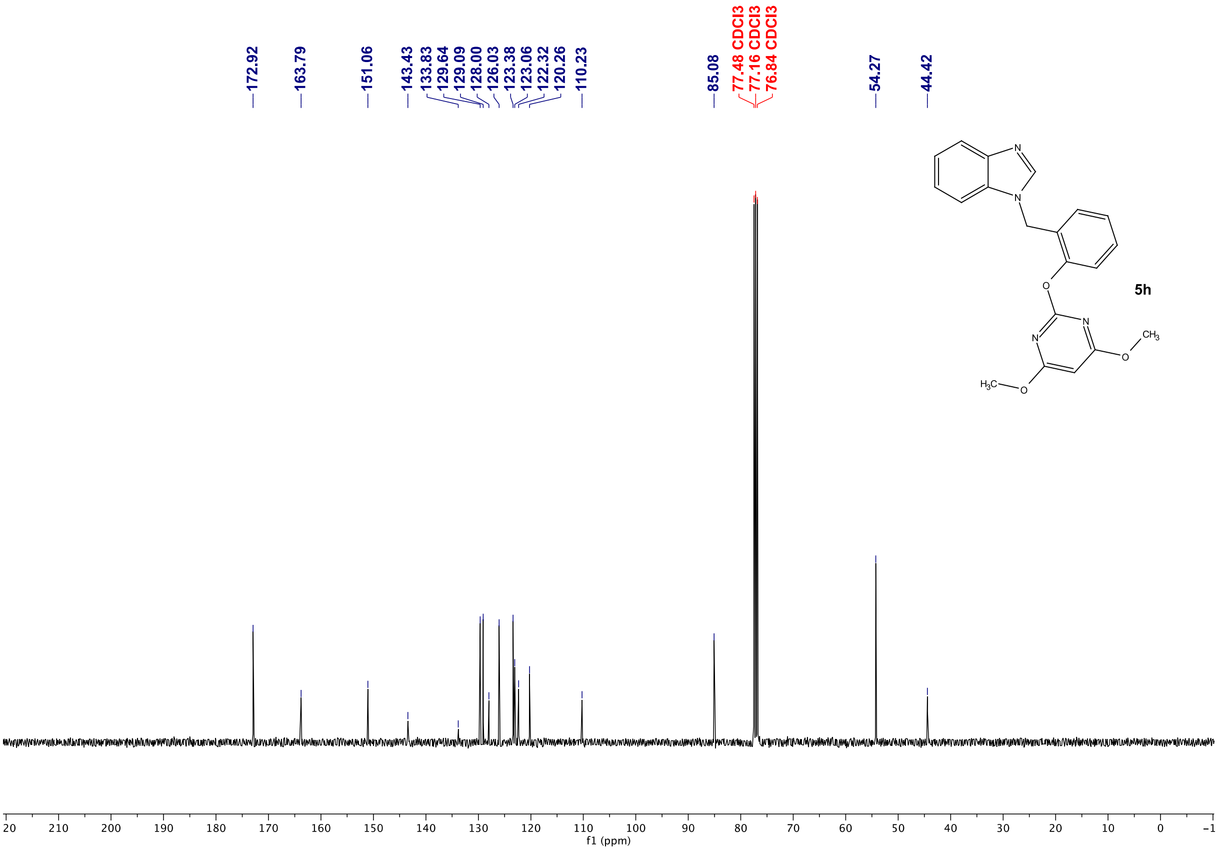

5i (400 MHz, $CDCl_3$)

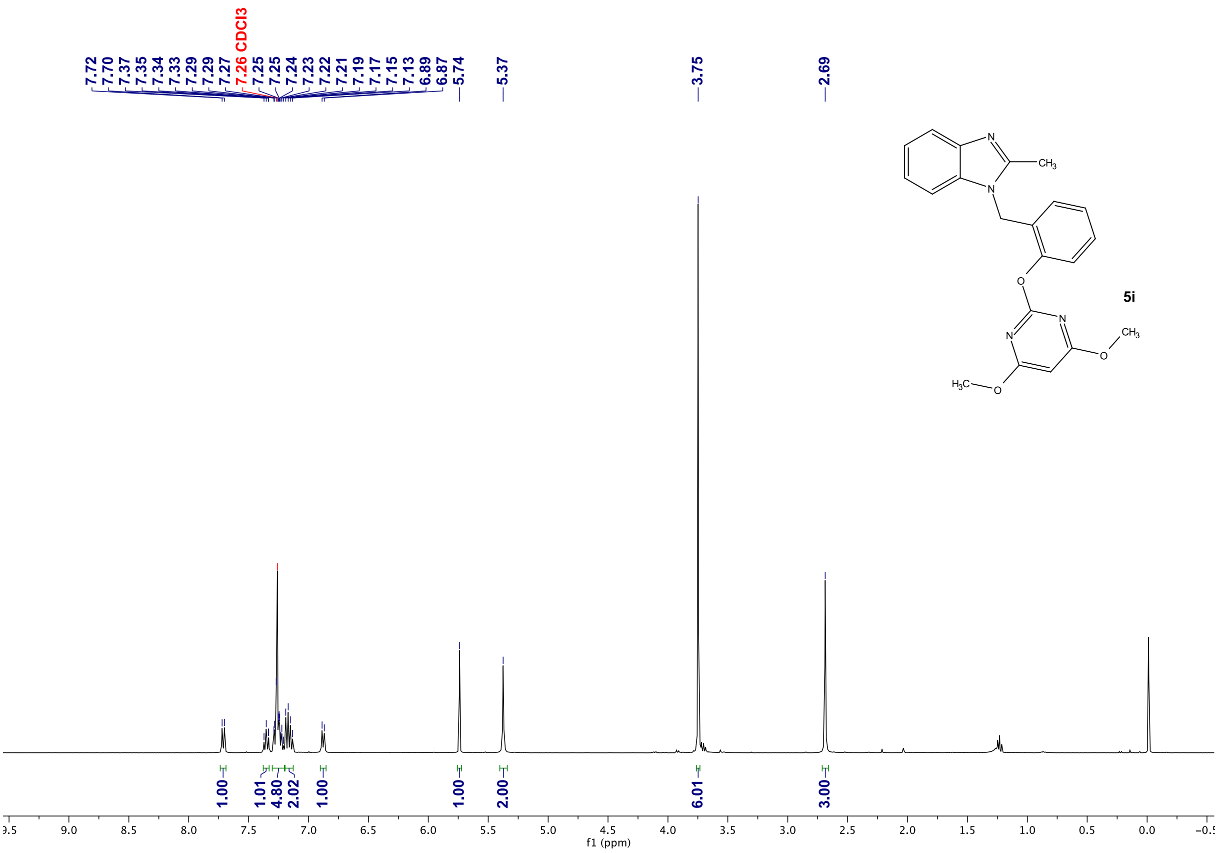

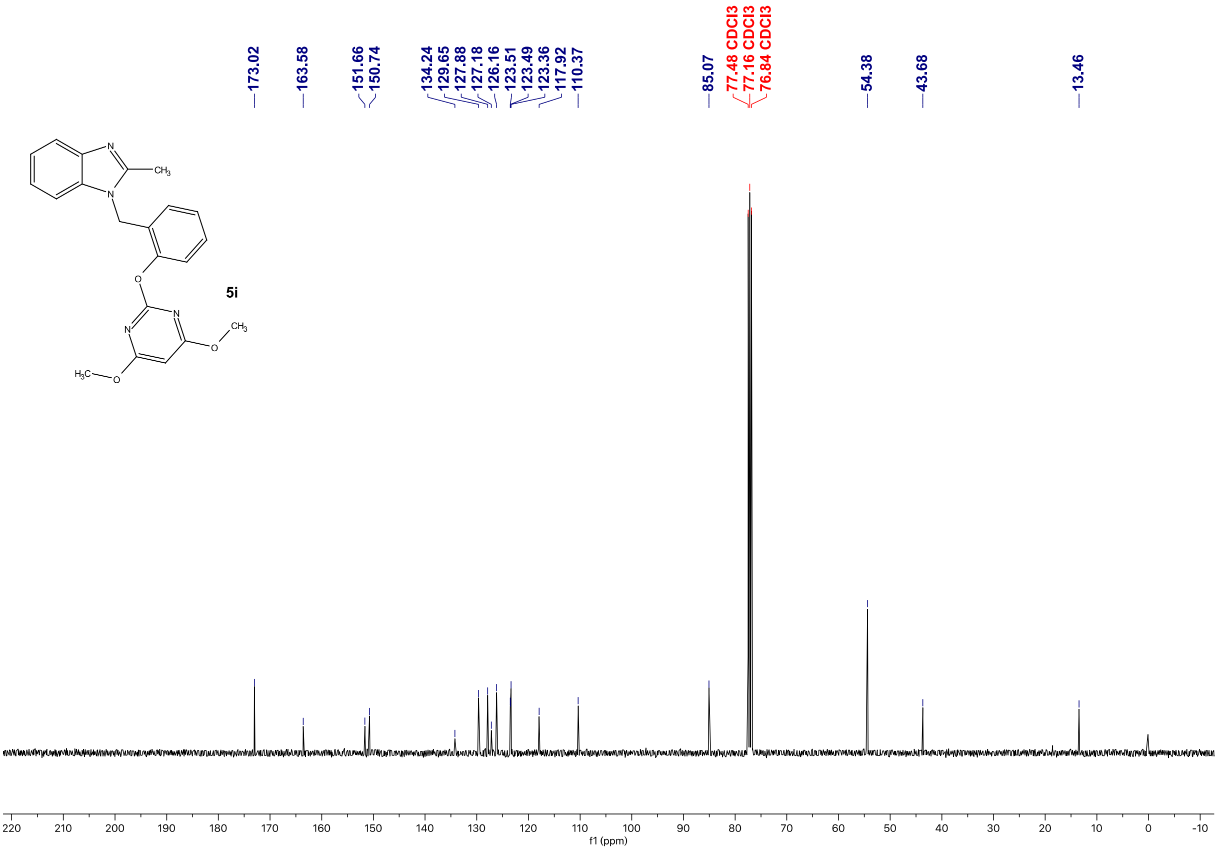

5j (400 MHz, $CDCl_3$)

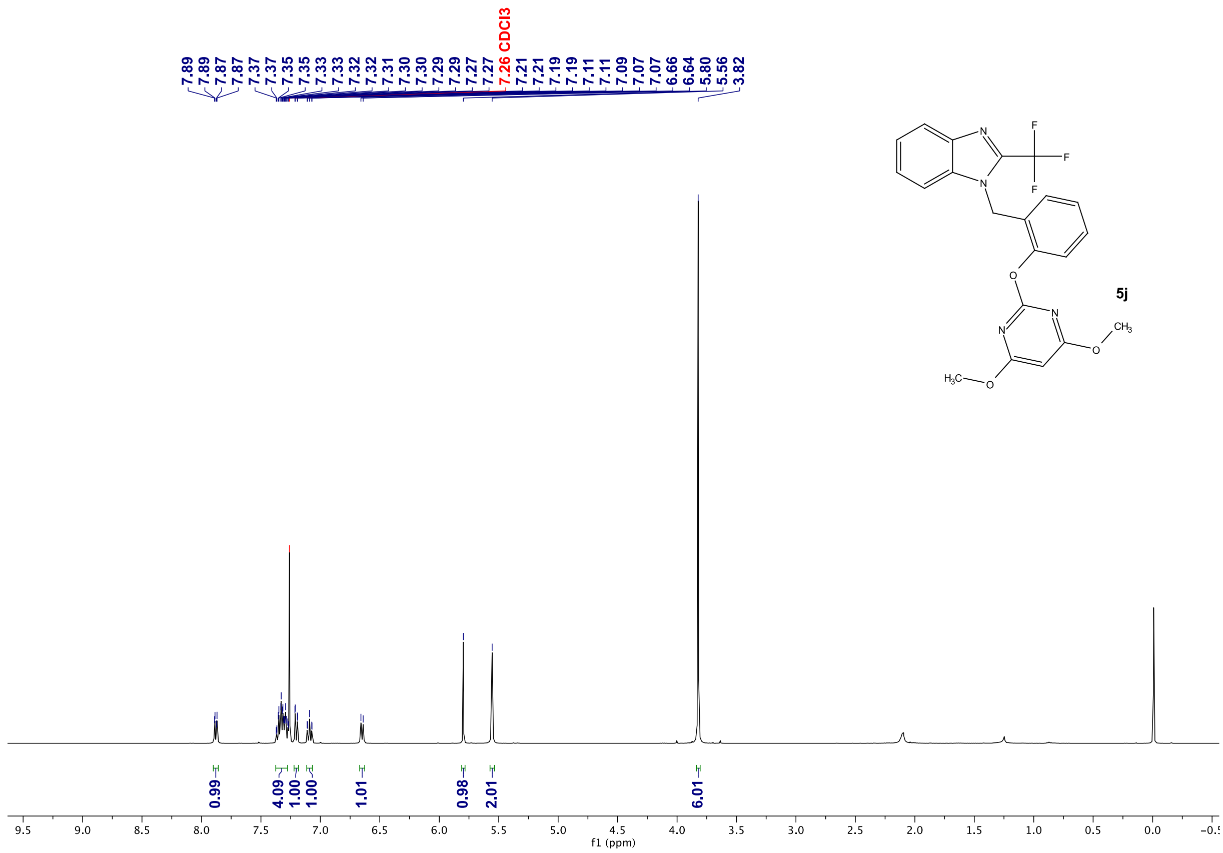

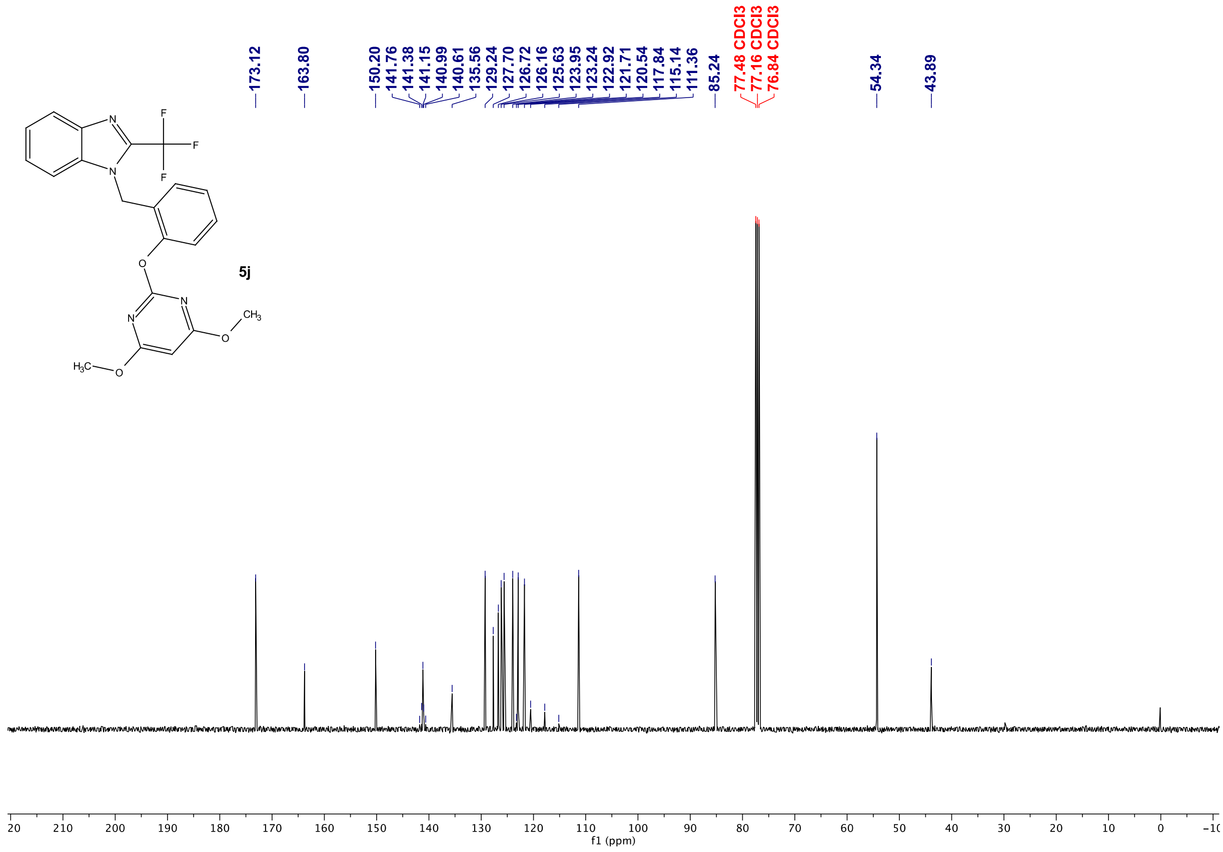

5k (400 MHz, $CDCl_3$)

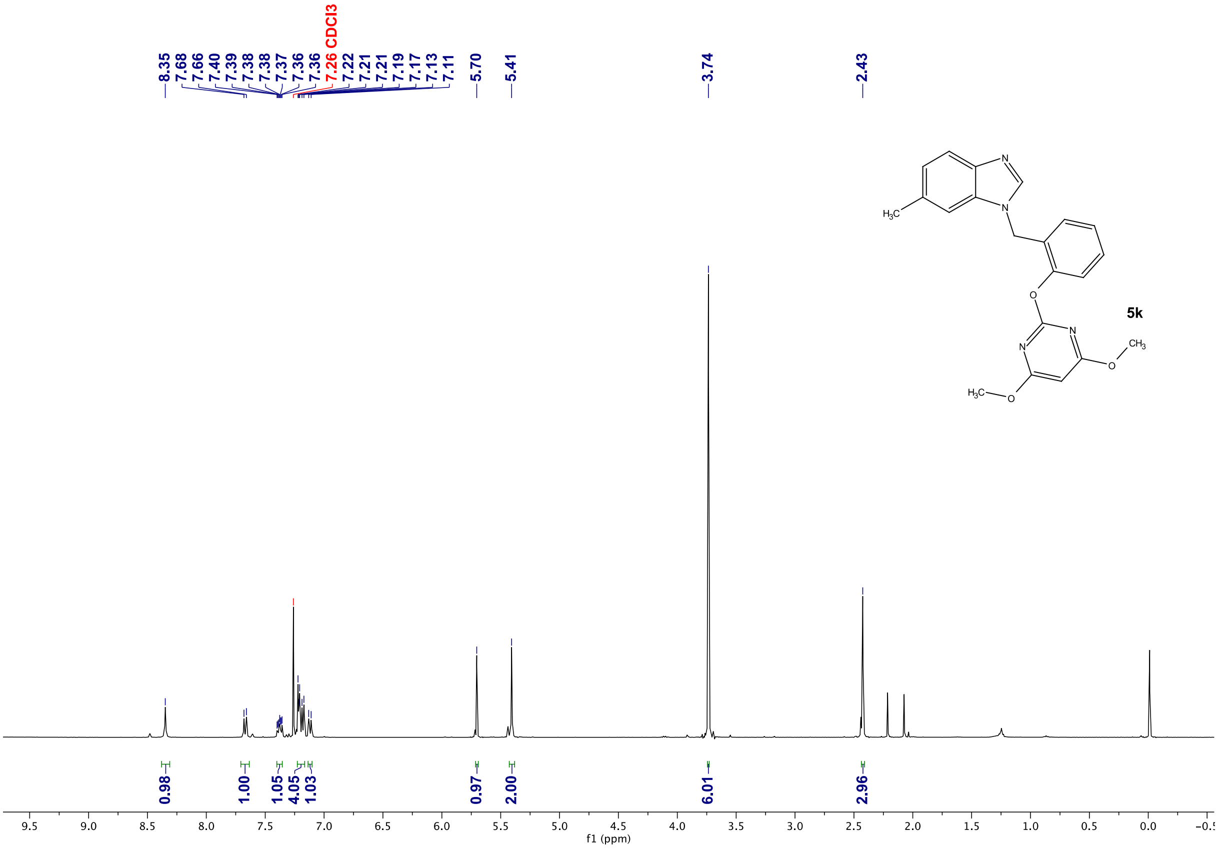

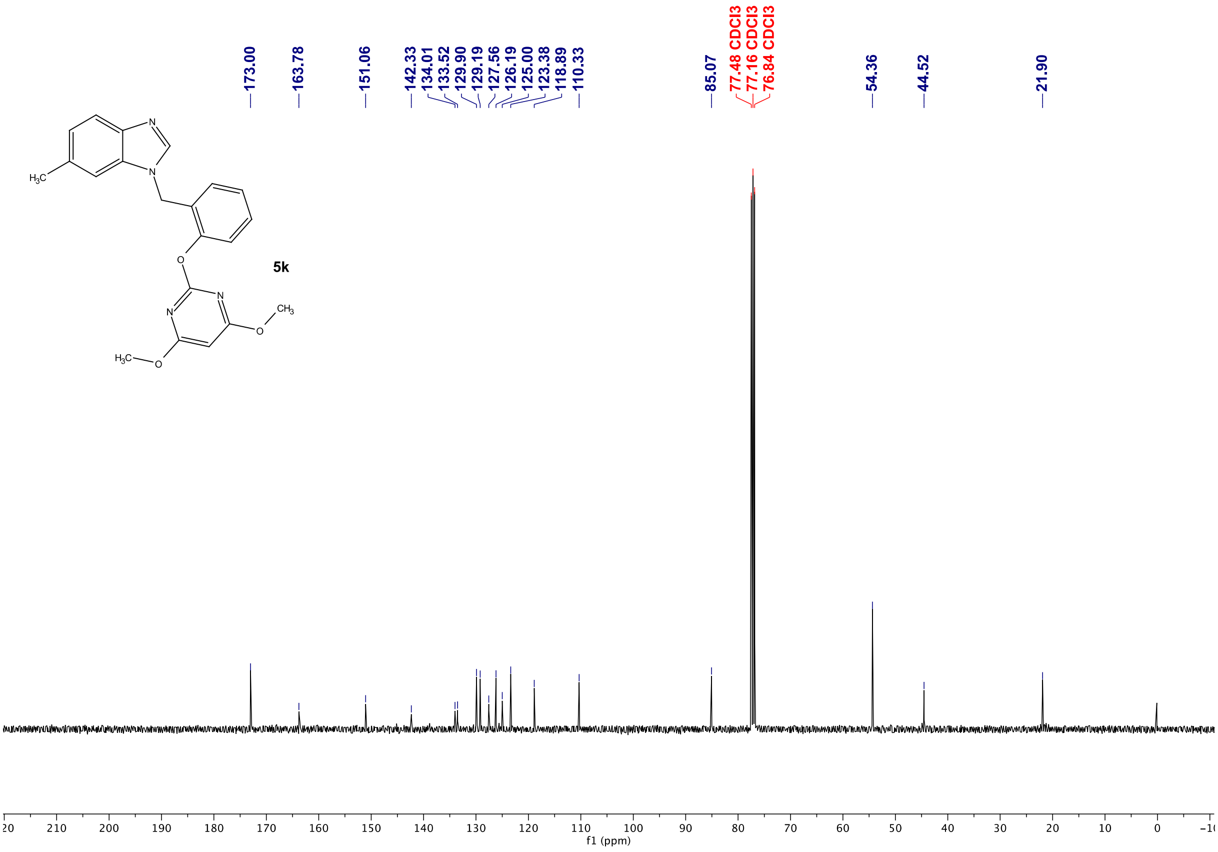

5l (400 MHz, $CDCl_3$)

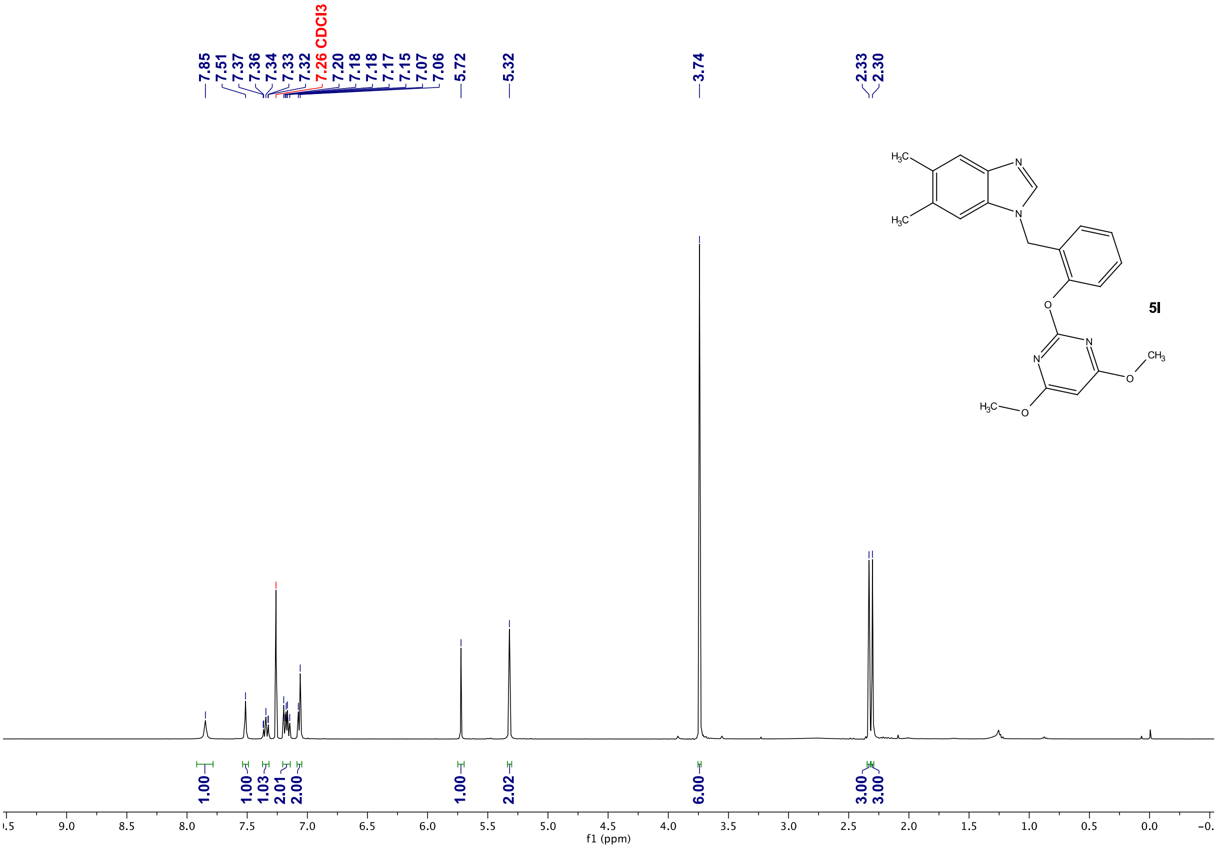

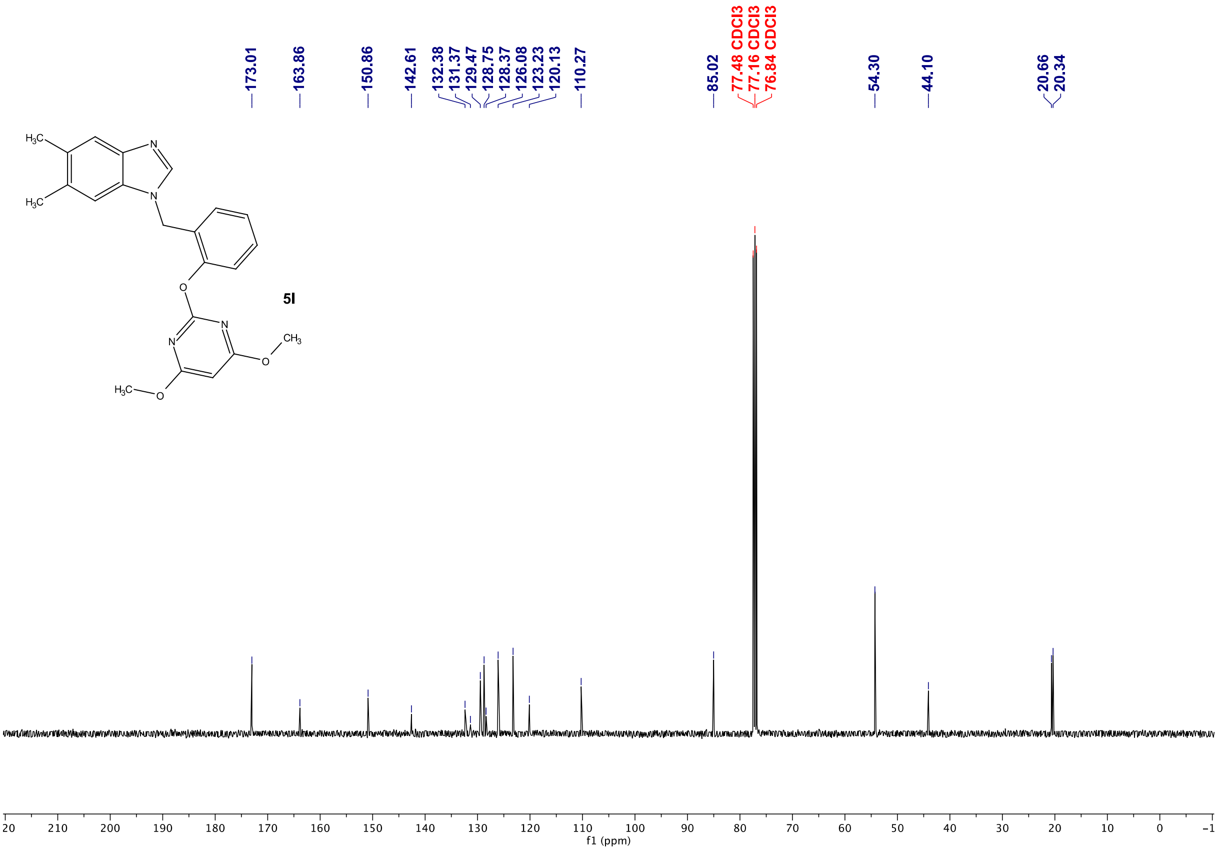

5m (400 MHz, $CDCl_3$)

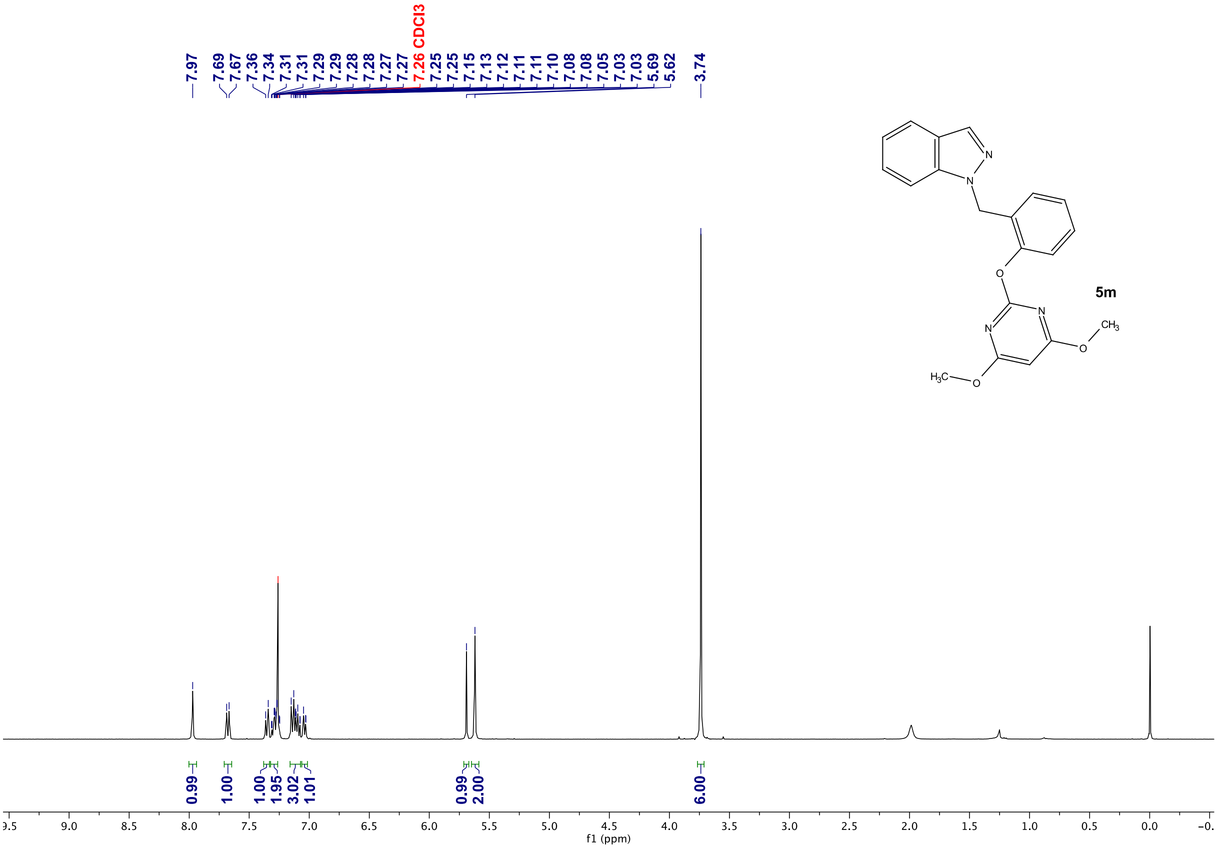

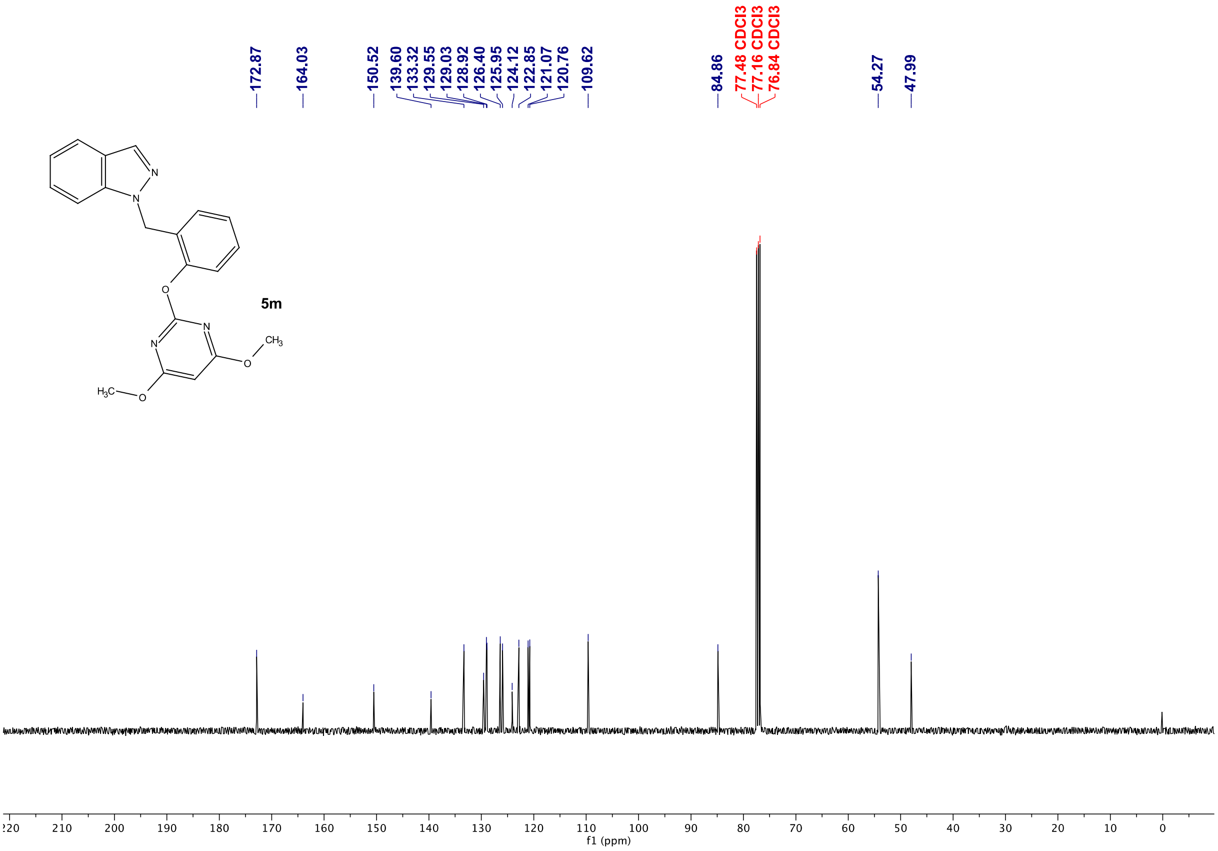

5n (400 MHz, DMSO-d_6)

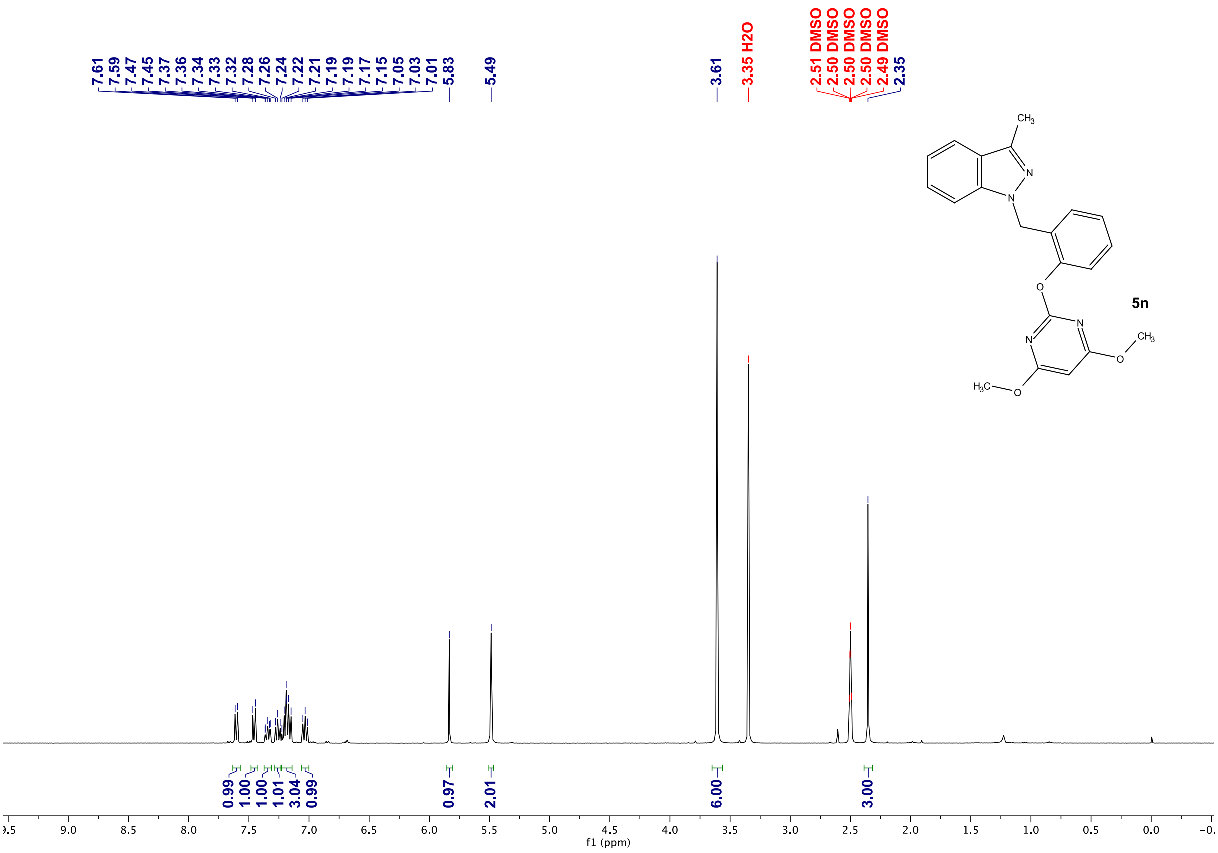

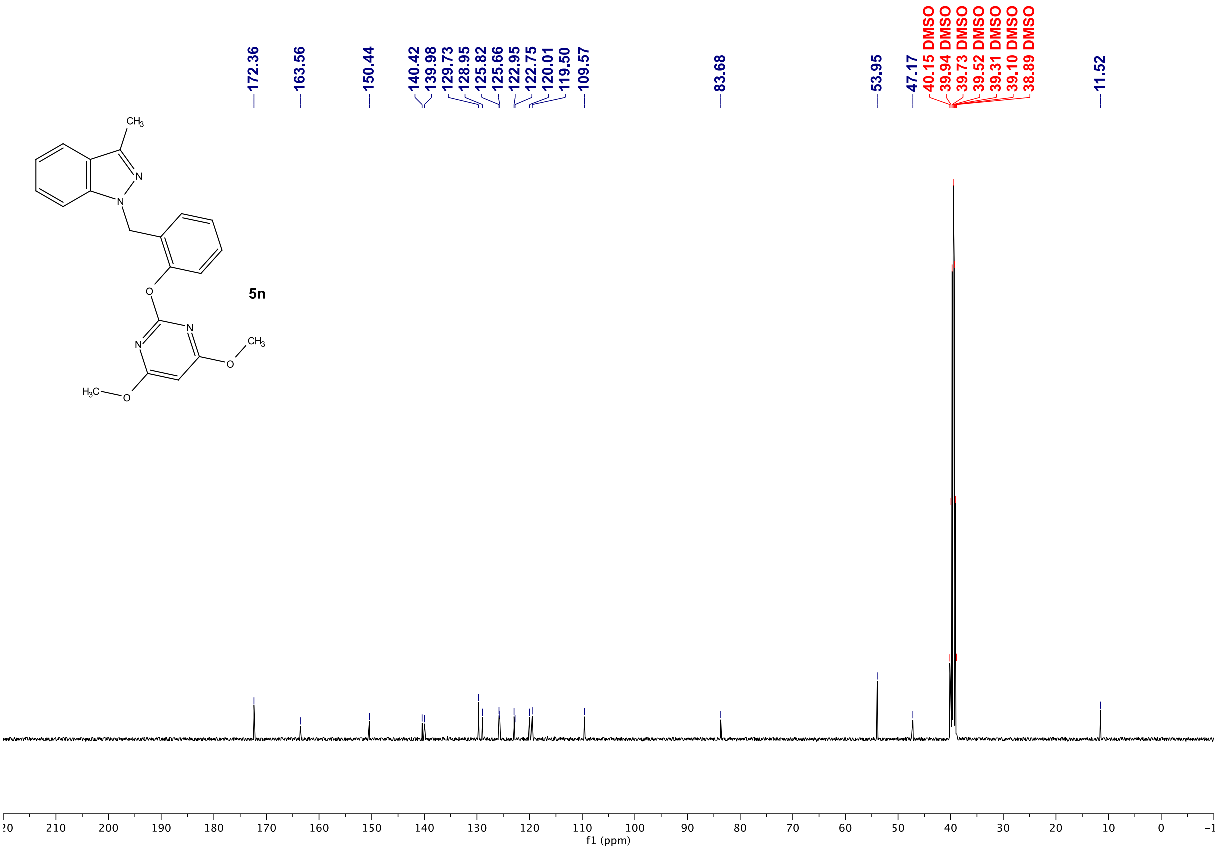

5o (400 MHz, $CDCl_3$)

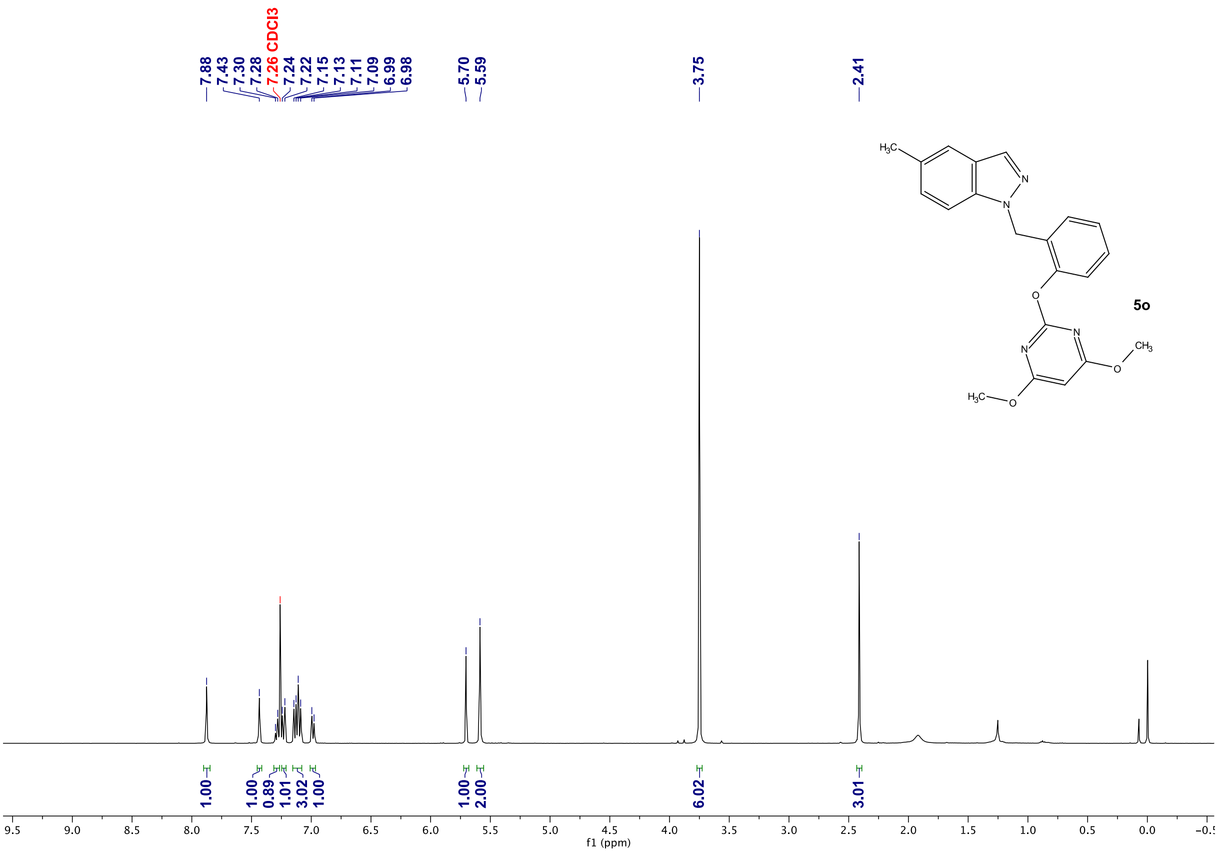

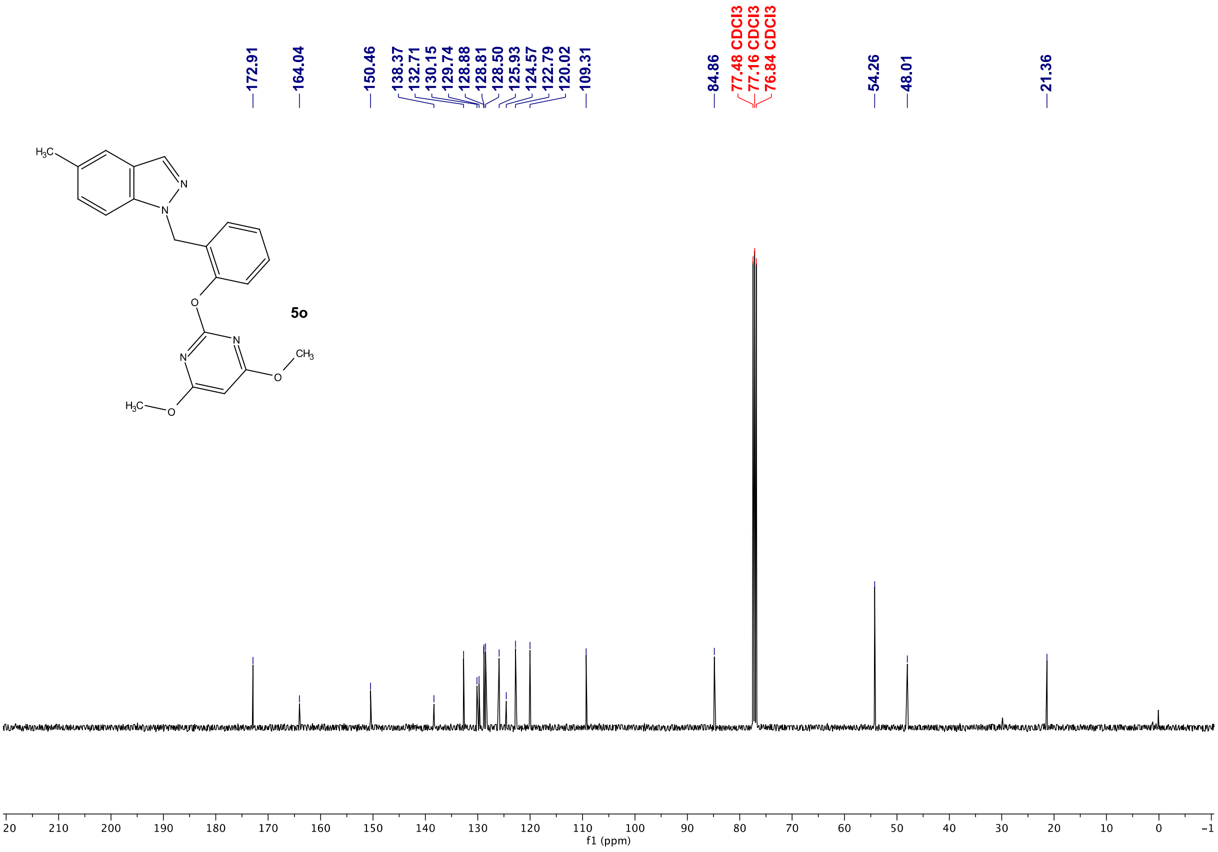

5p (400 MHz, $CDCl_3$)

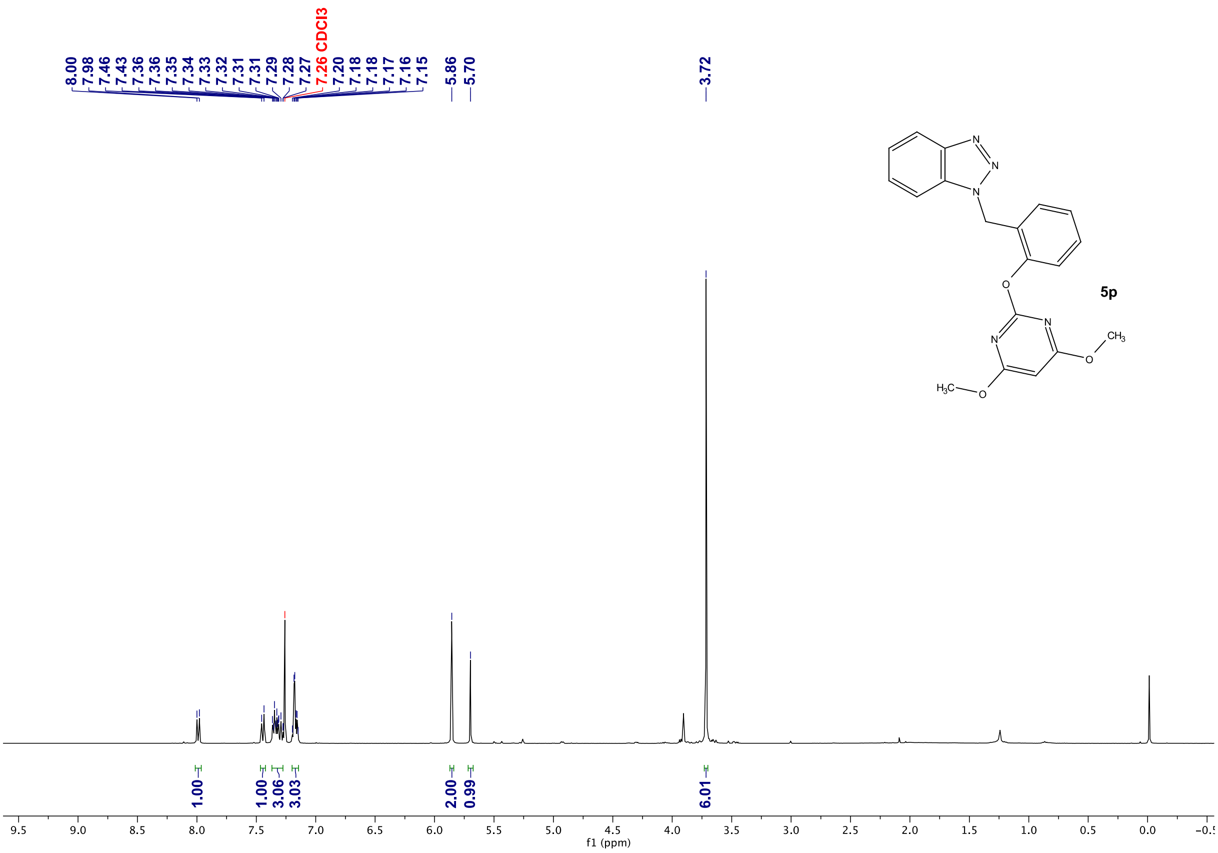

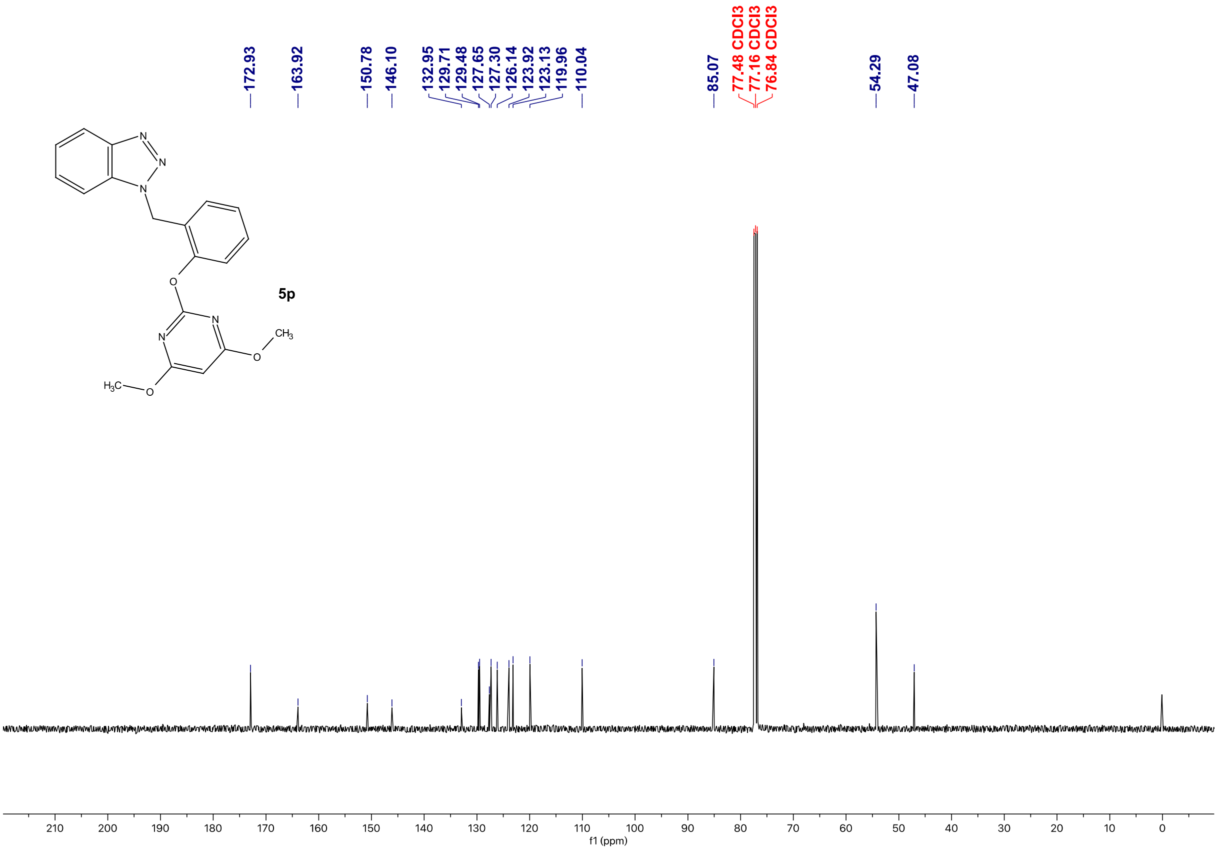

5q (400 MHz, $CDCl_3$)

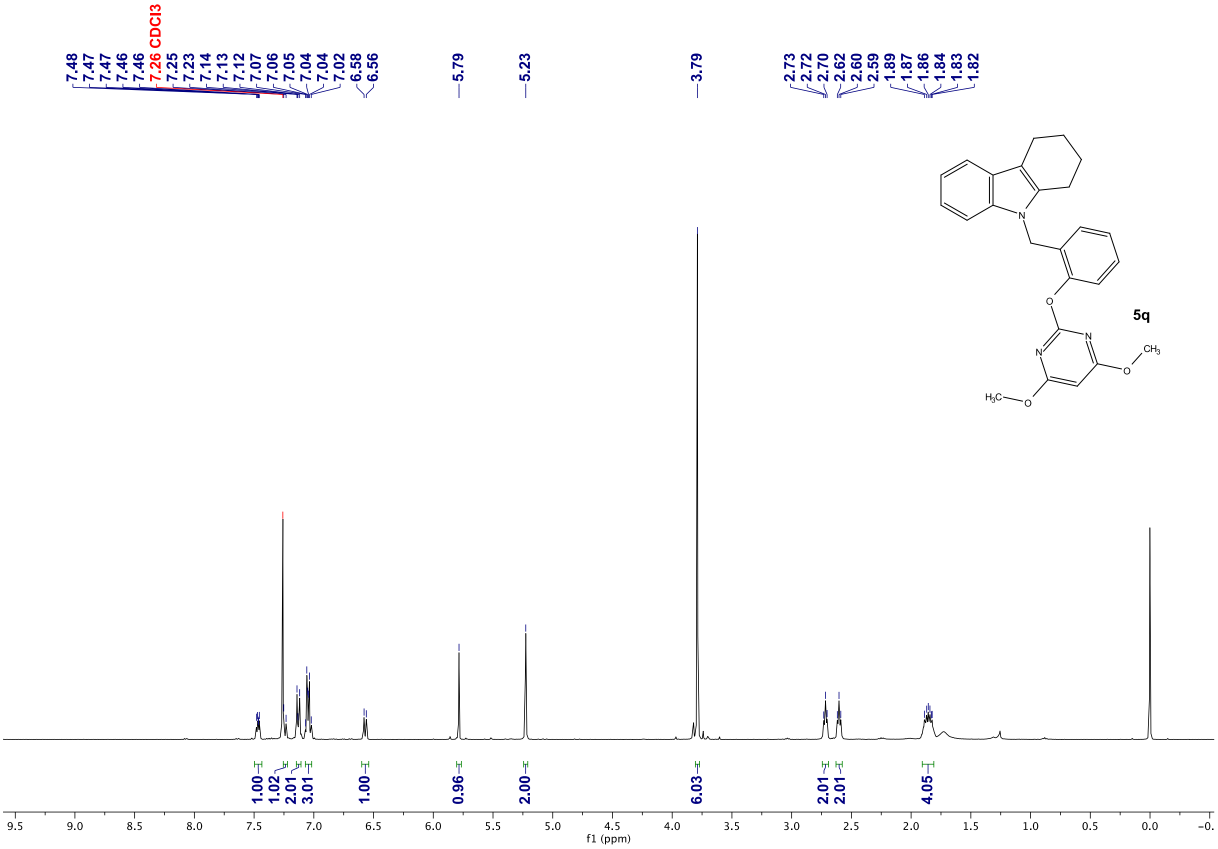

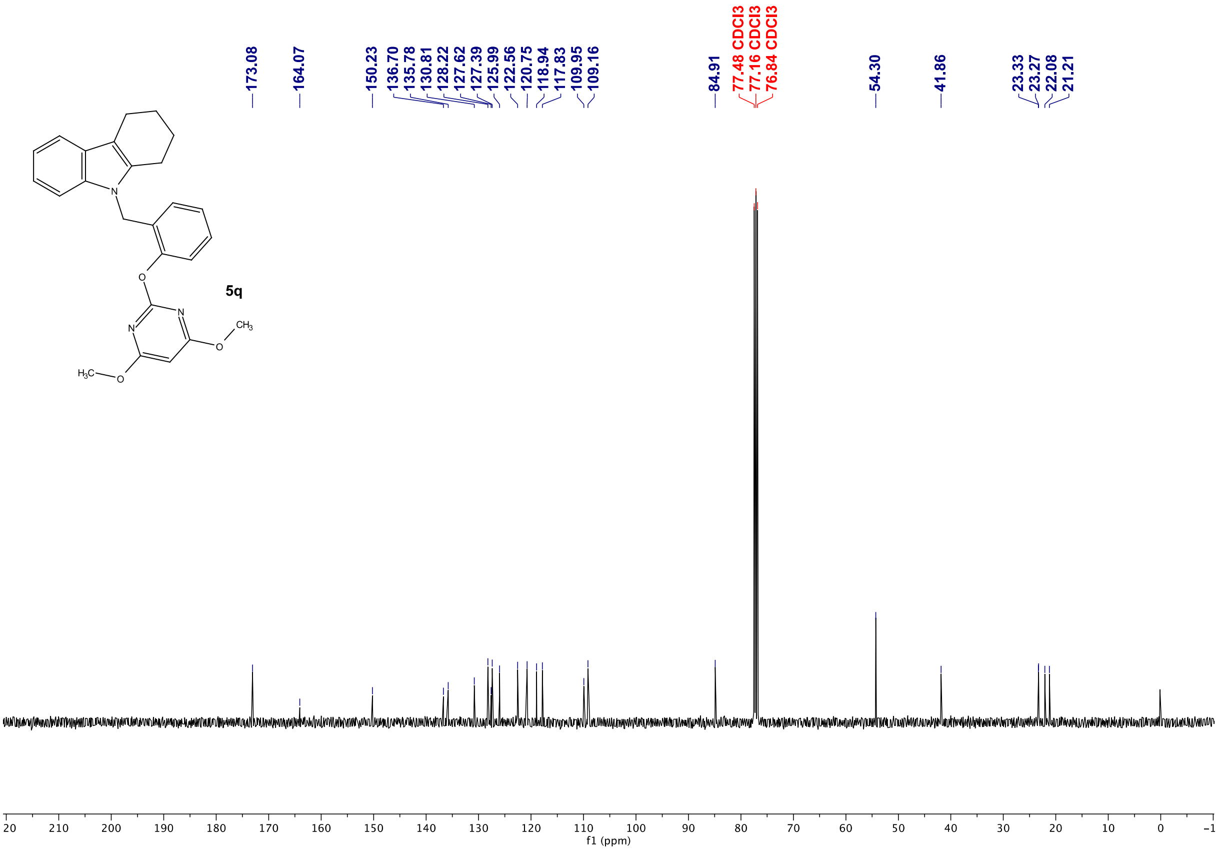

5r (400 MHz, $CDCl_3$)

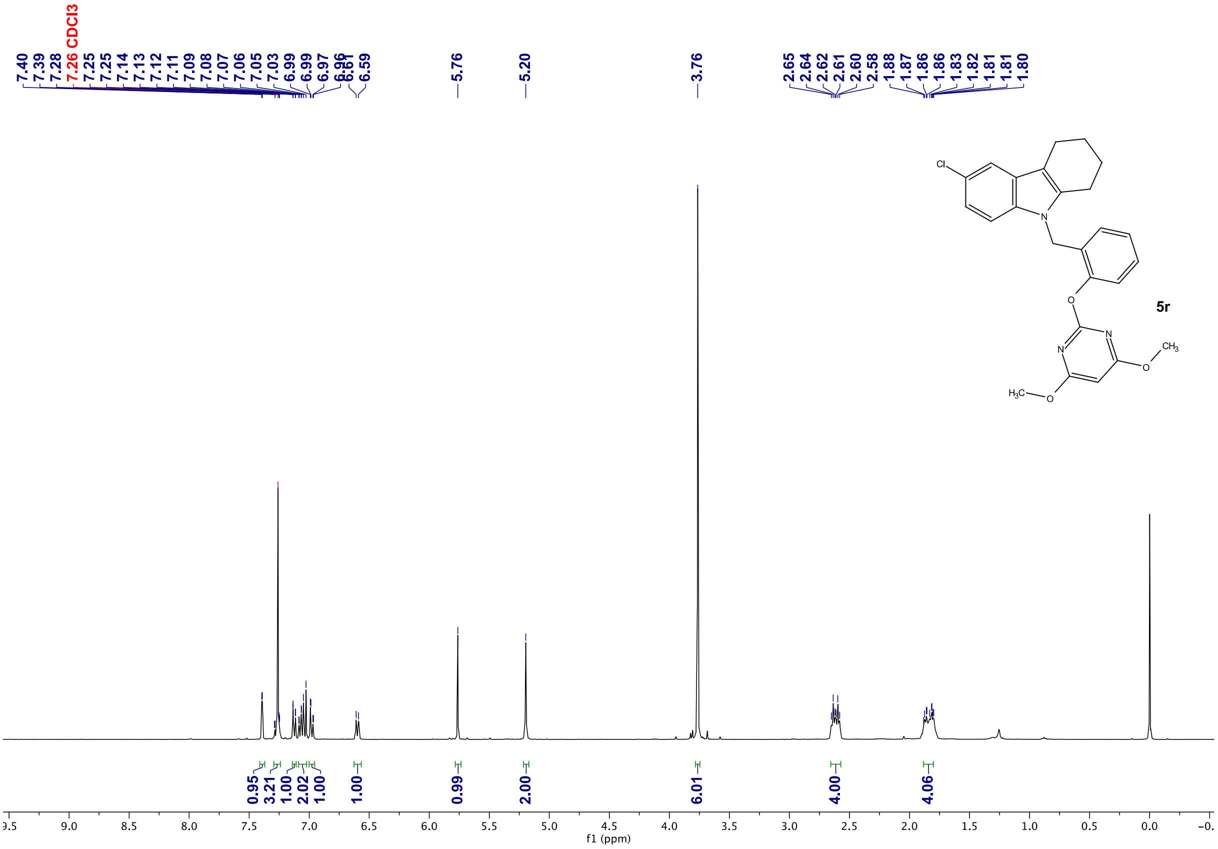

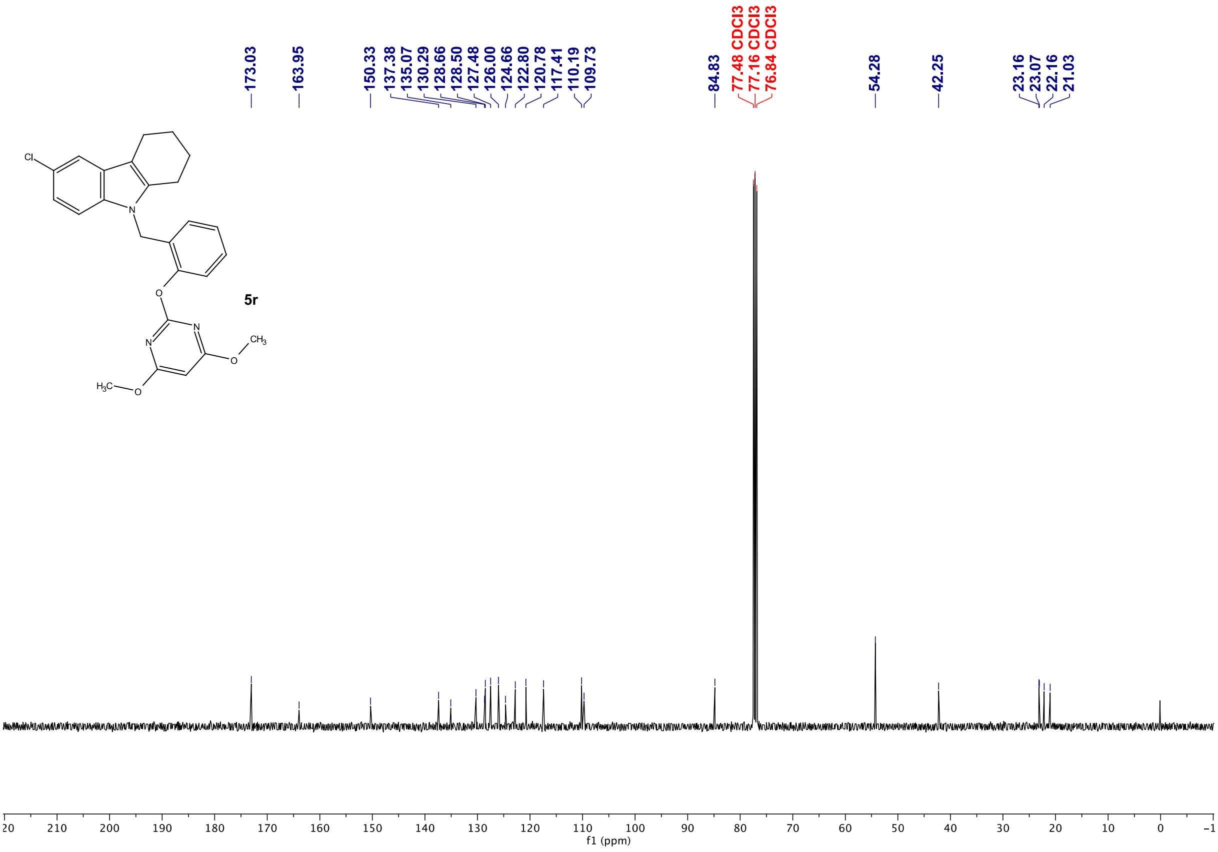

5s (400 MHz, $CDCl_3$)

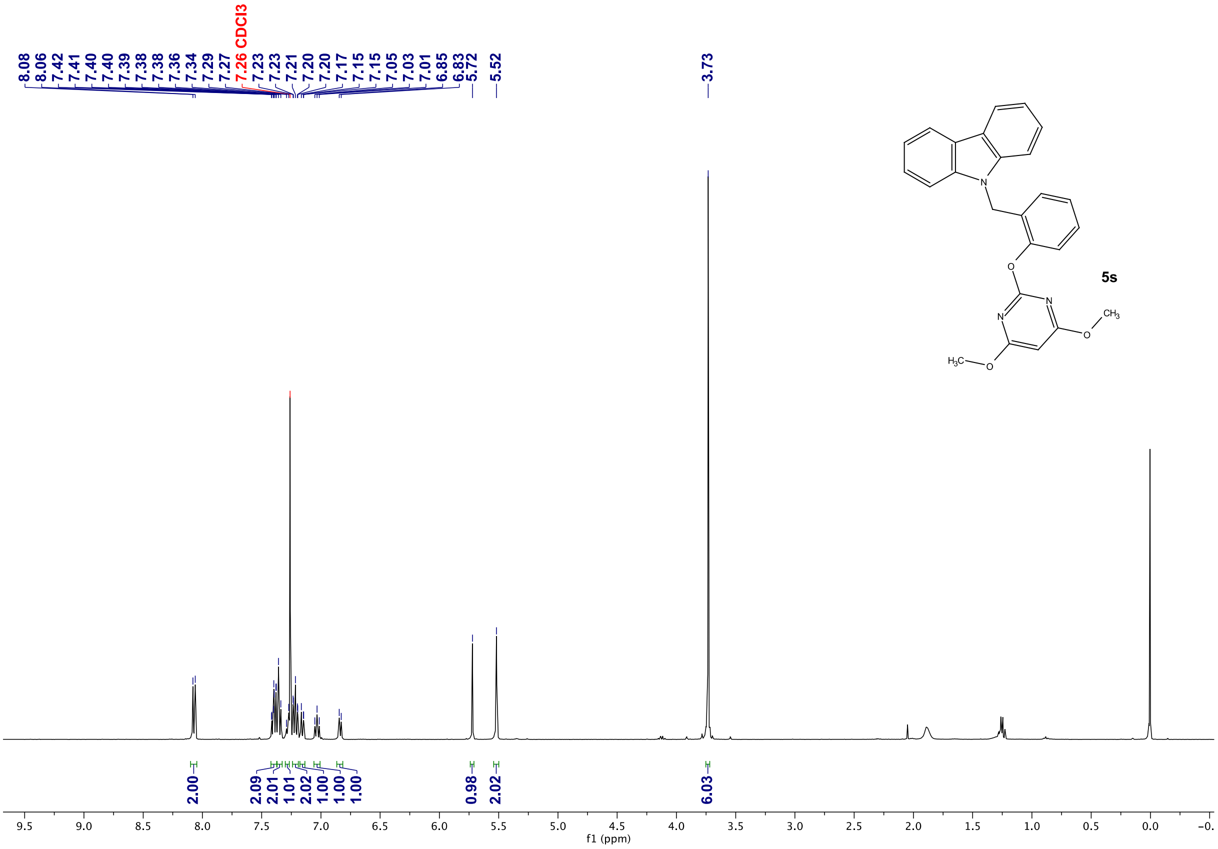

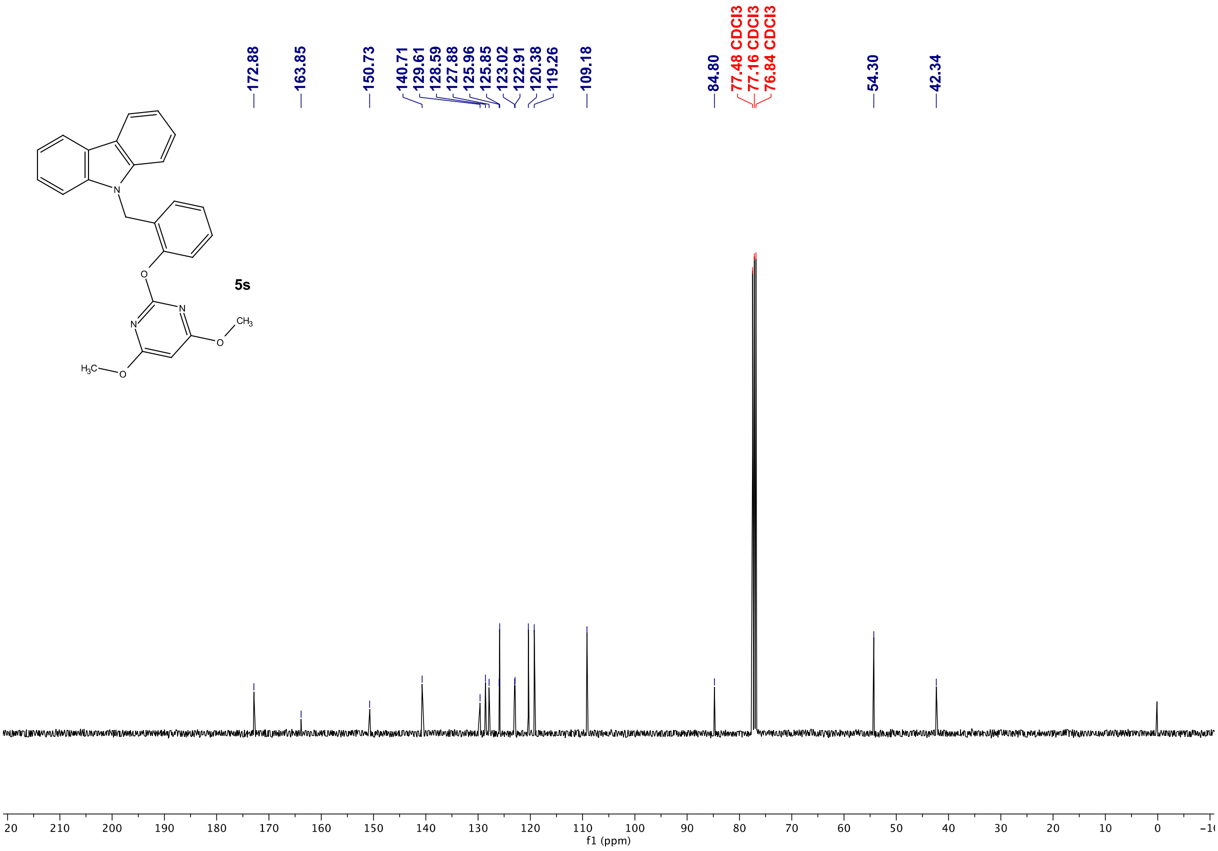

8a (400 MHz, $CDCl_3$)

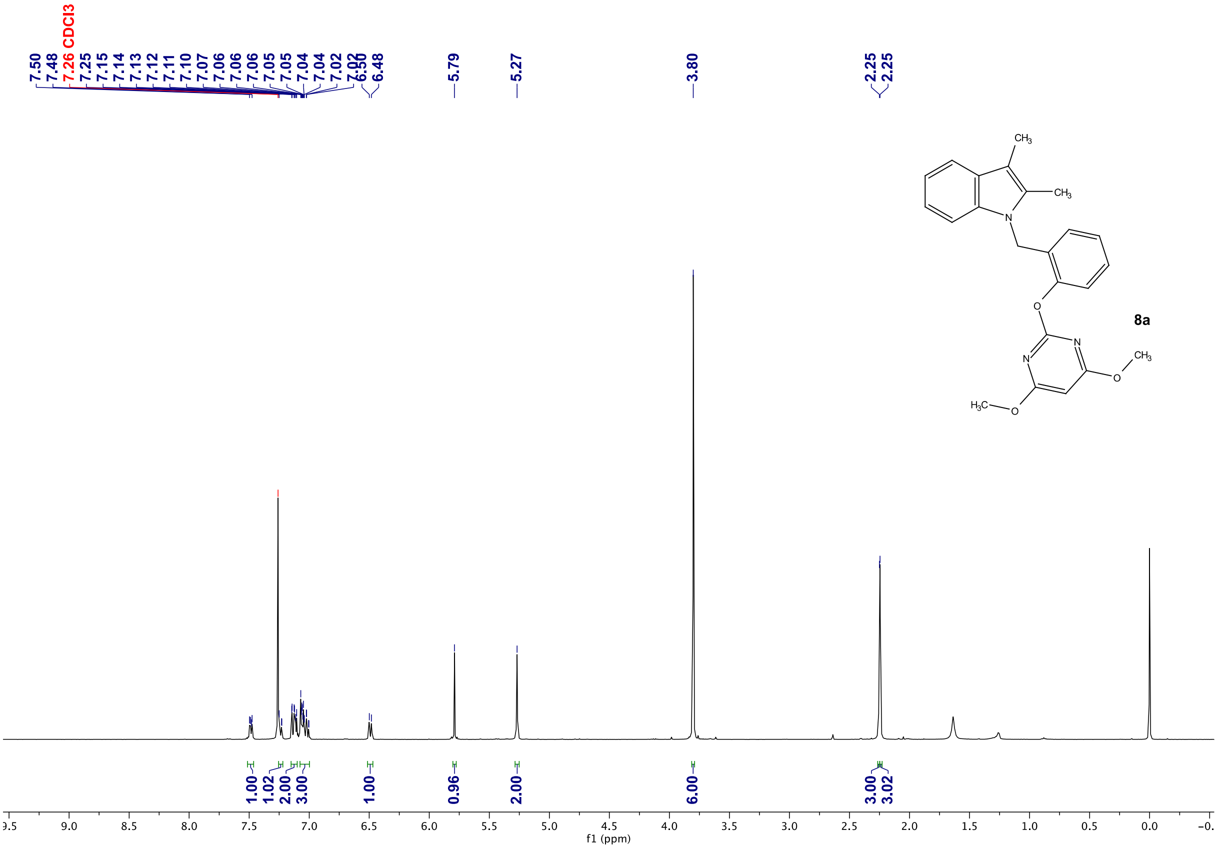

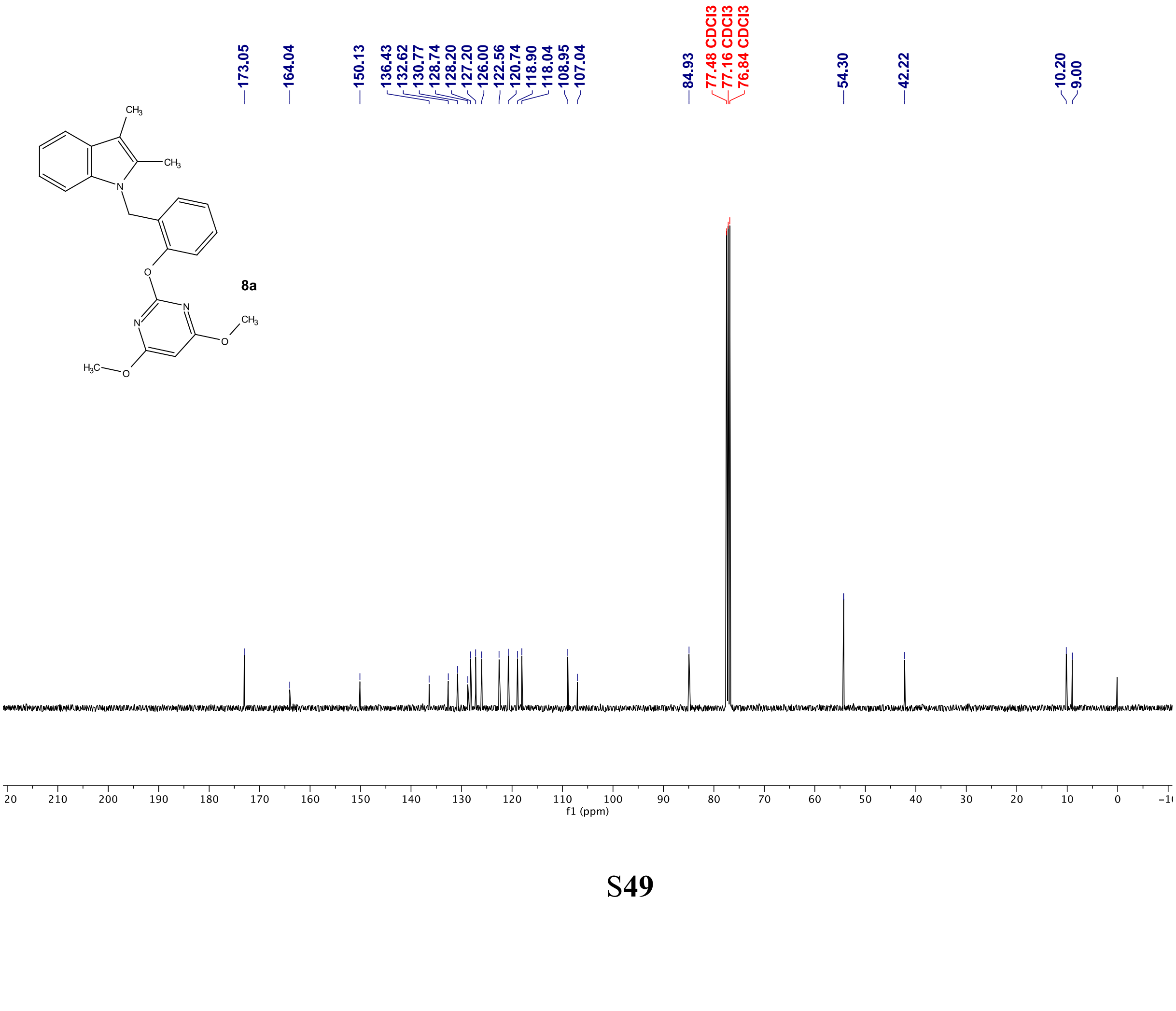

8b (400 MHz, $CDCl_3$)

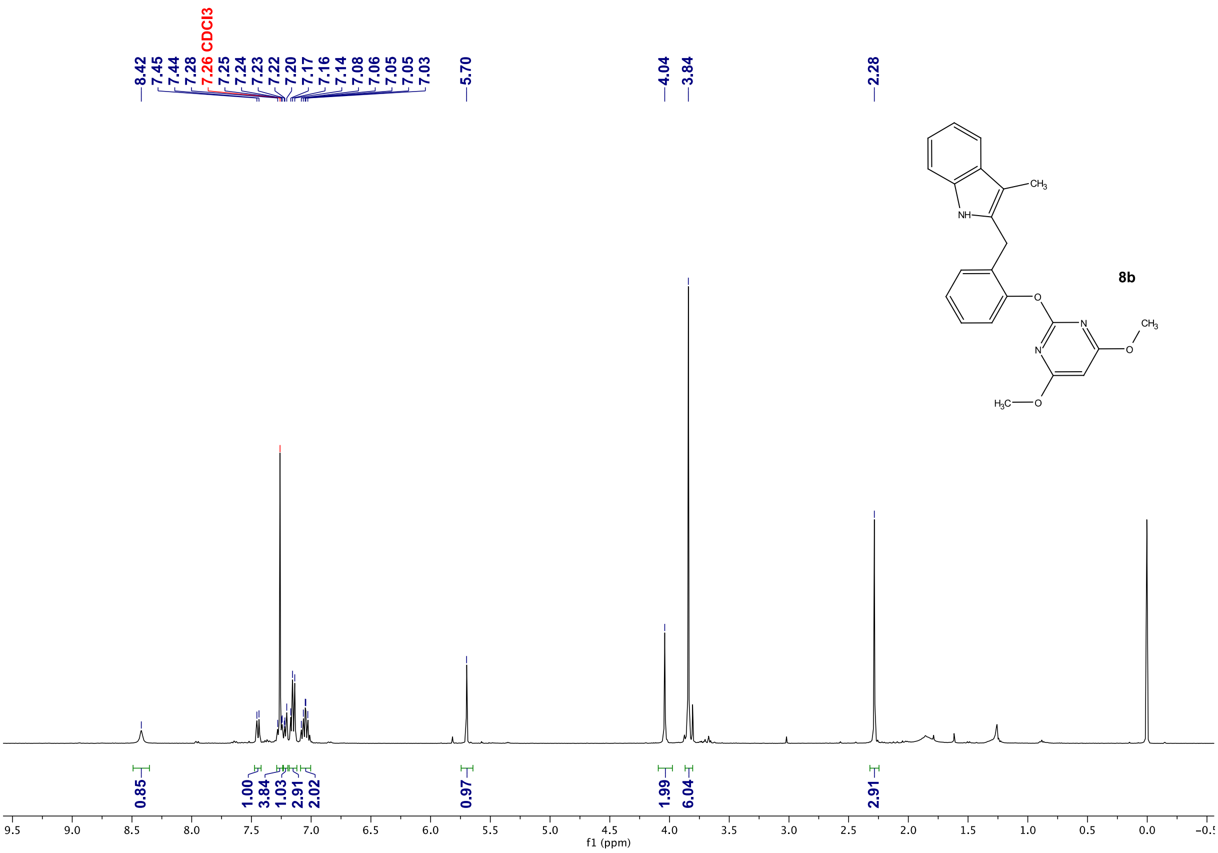

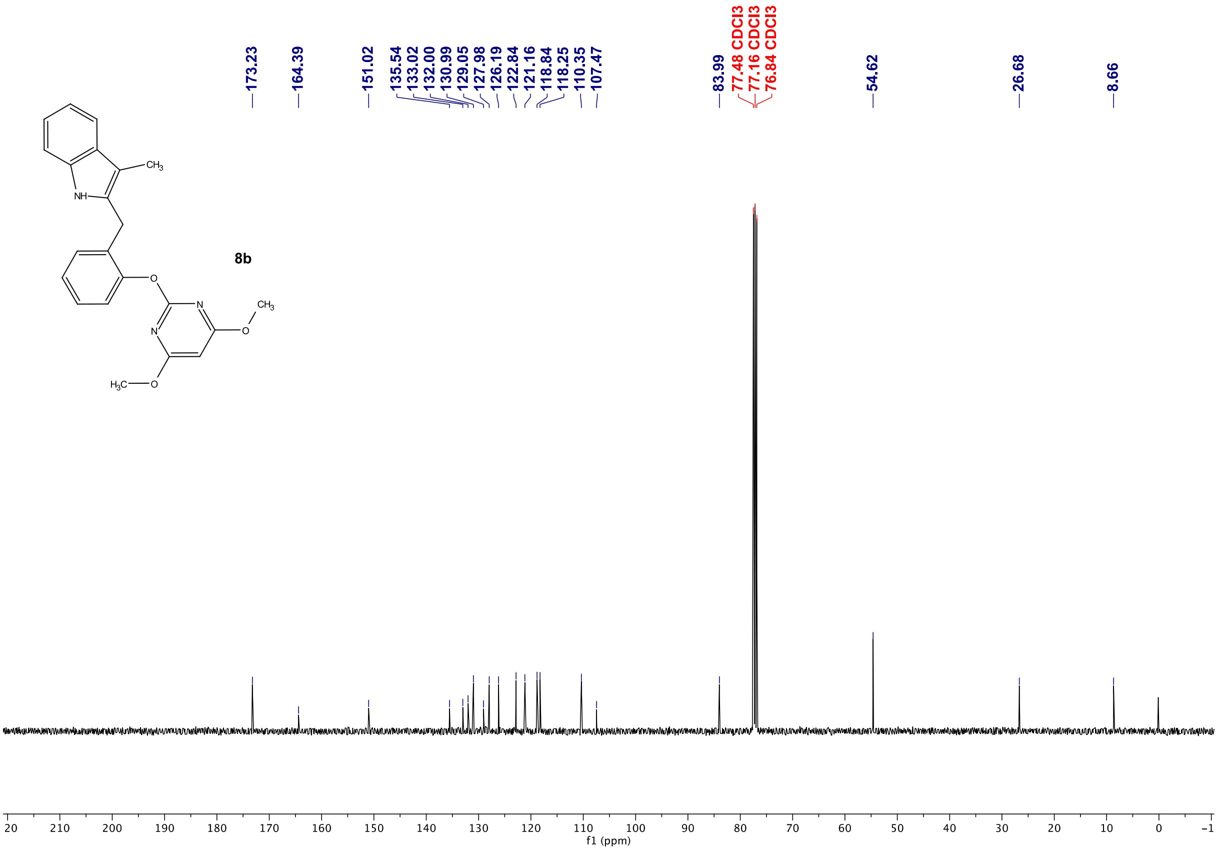

8c (400 MHz, $CDCl_3$)

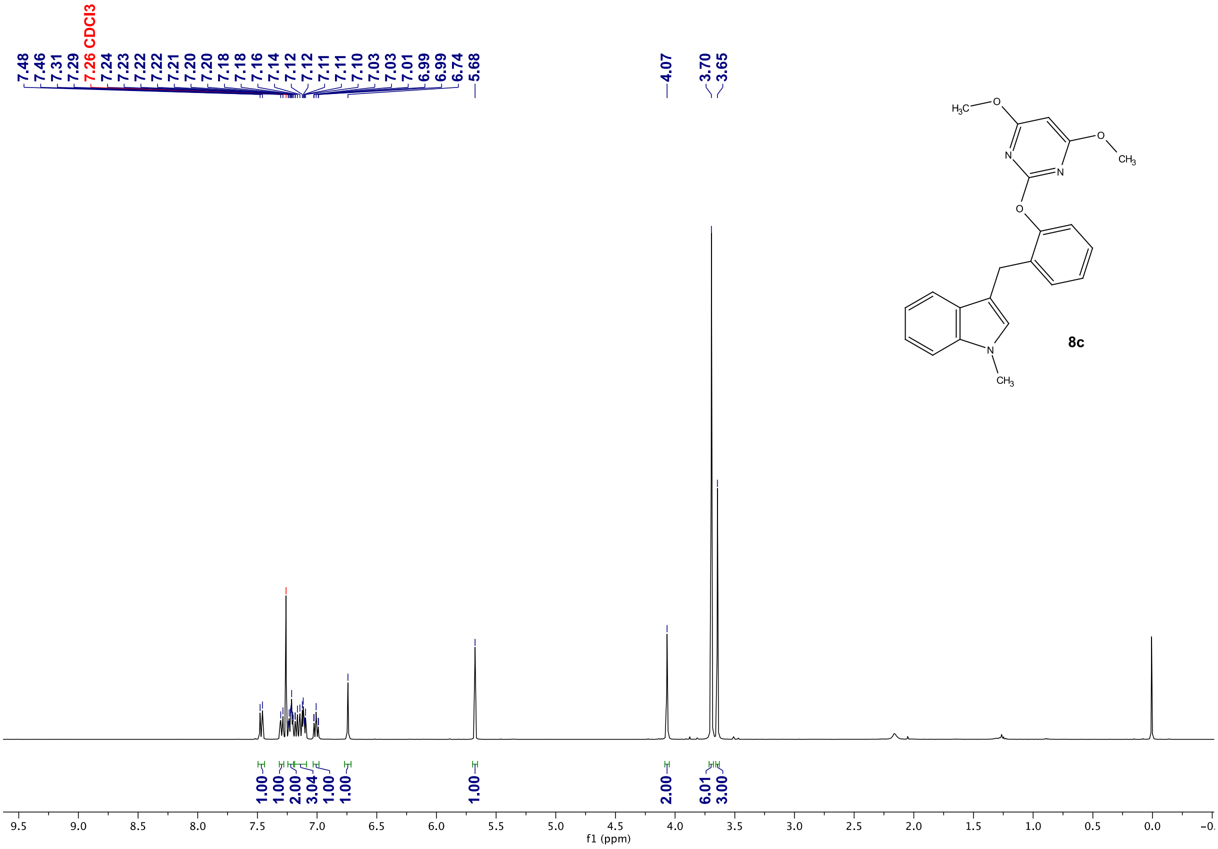

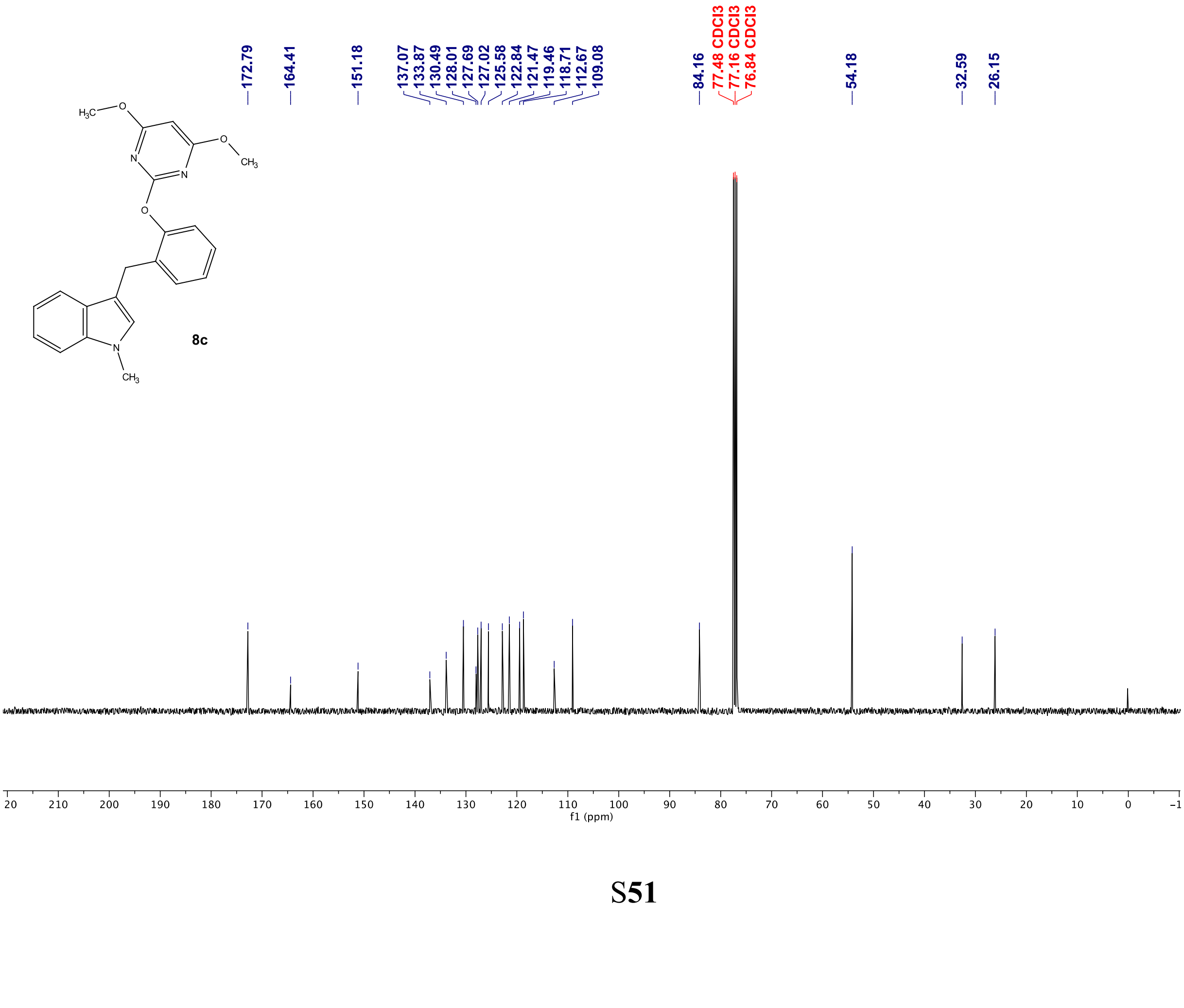

8d (400 MHz, $CDCl_3$)

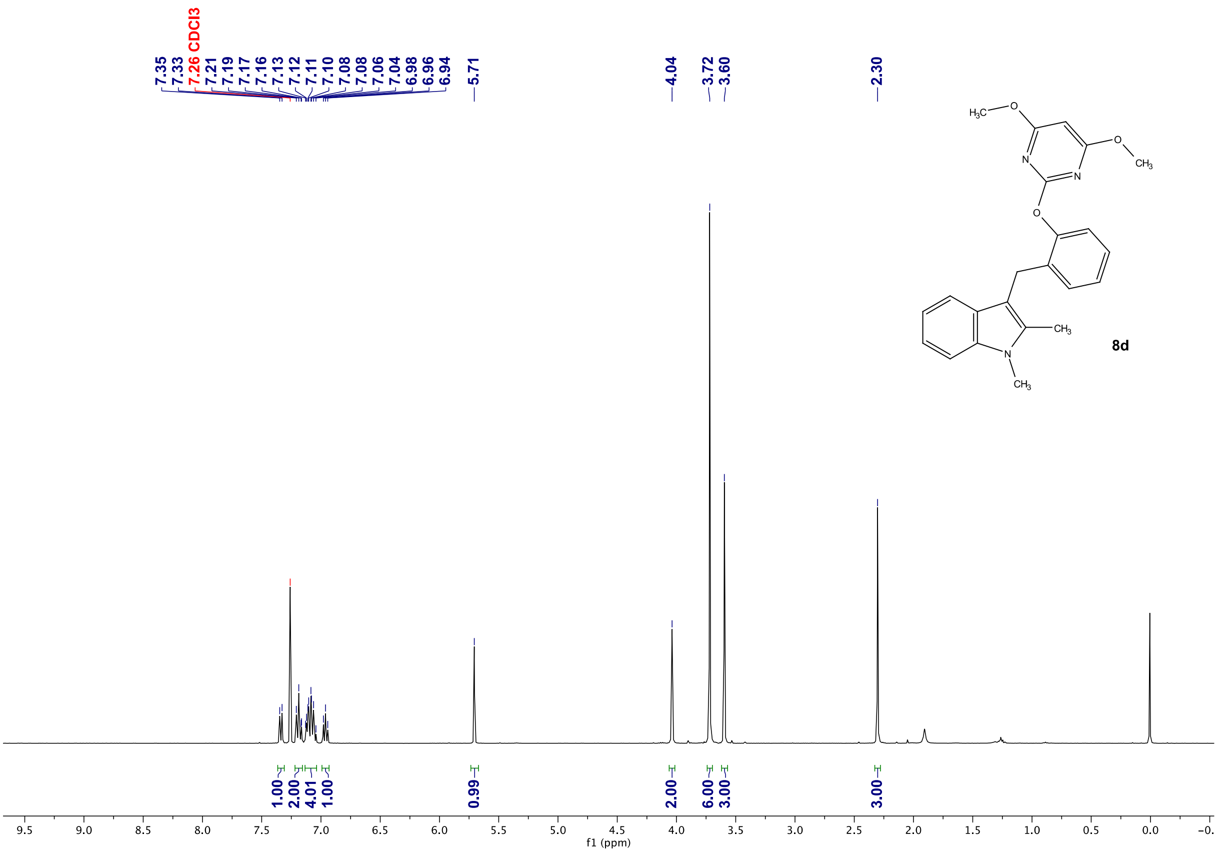

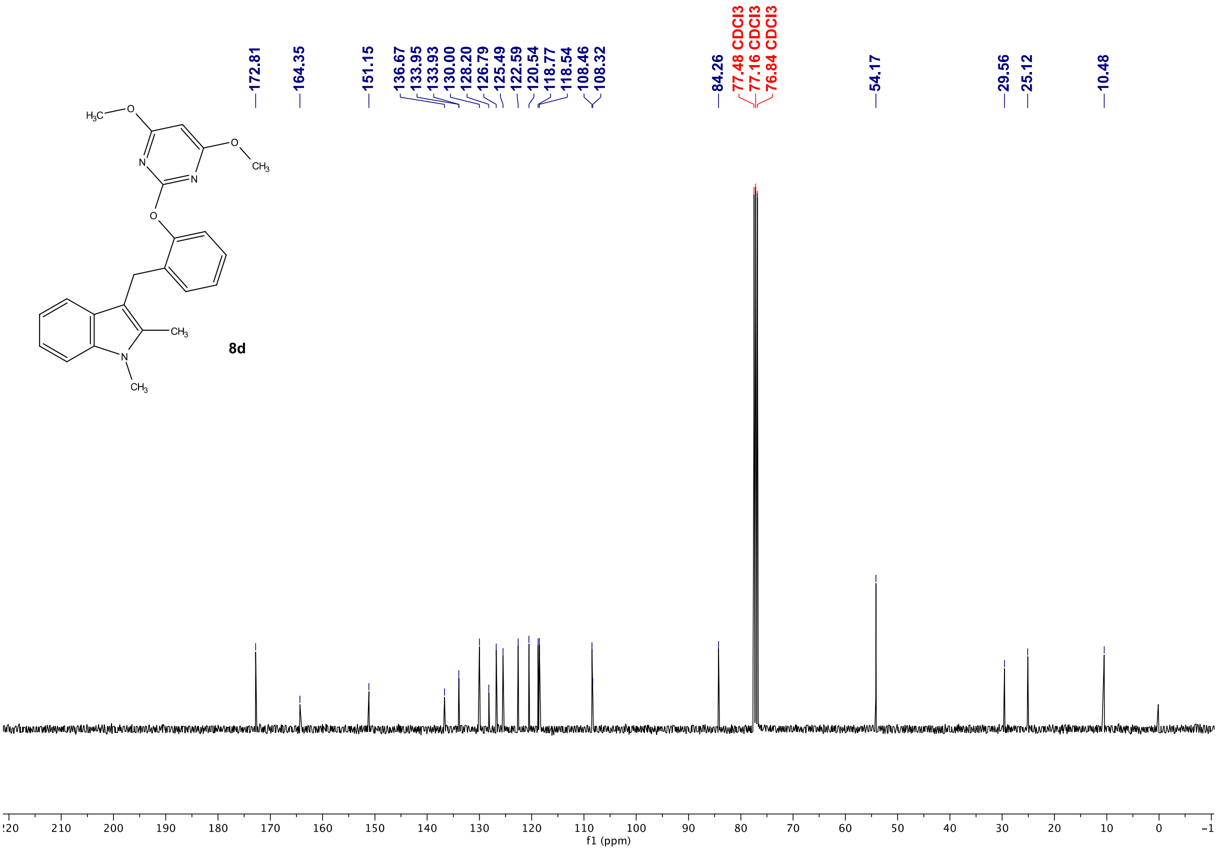

8e (400 MHz, $CDCl_3$)

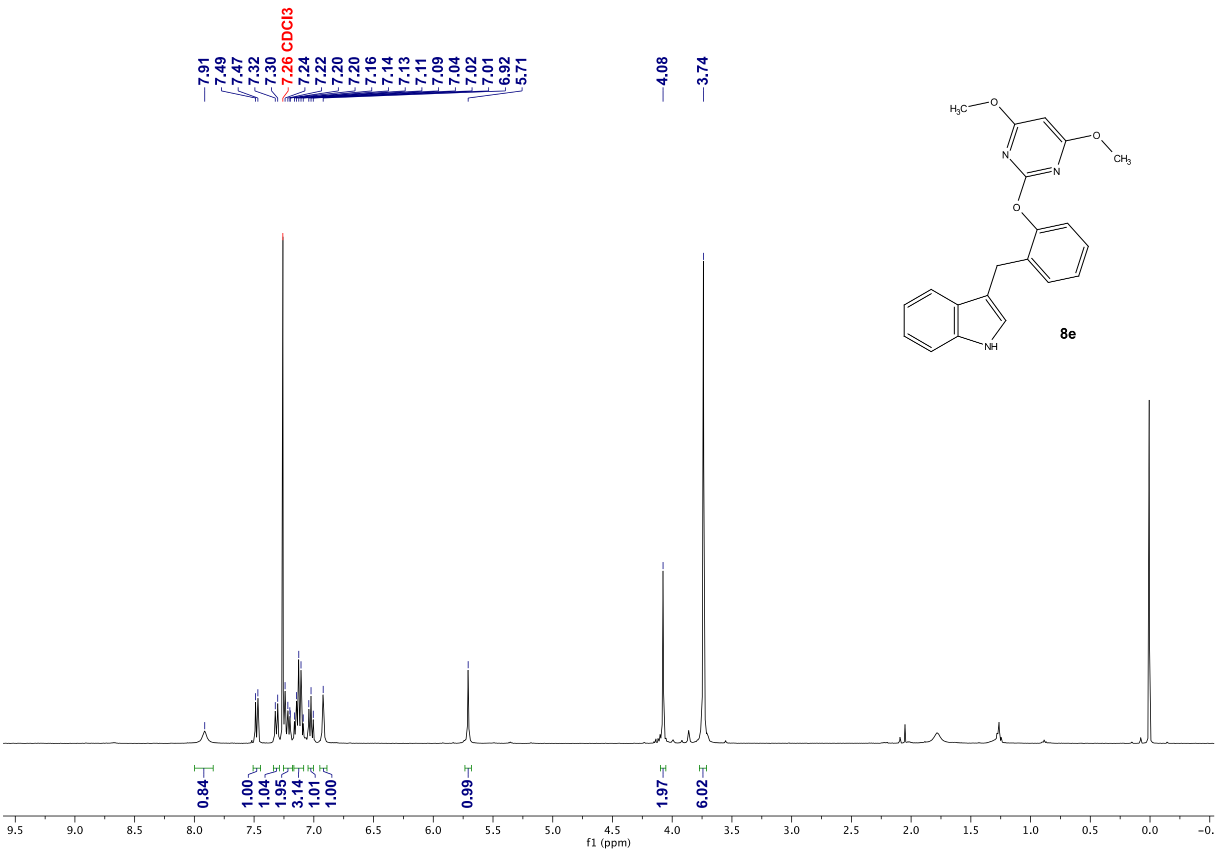

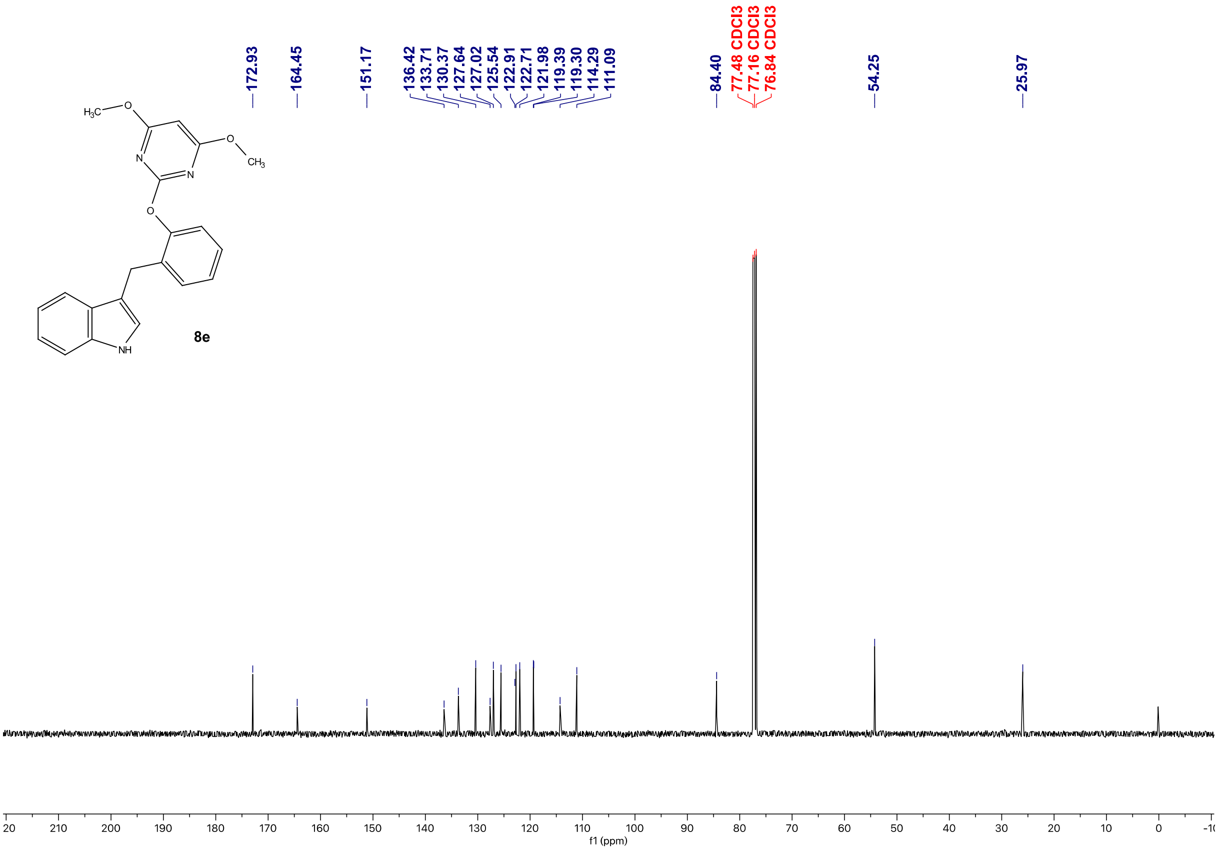

8f (400 MHz, $CDCl_3$)

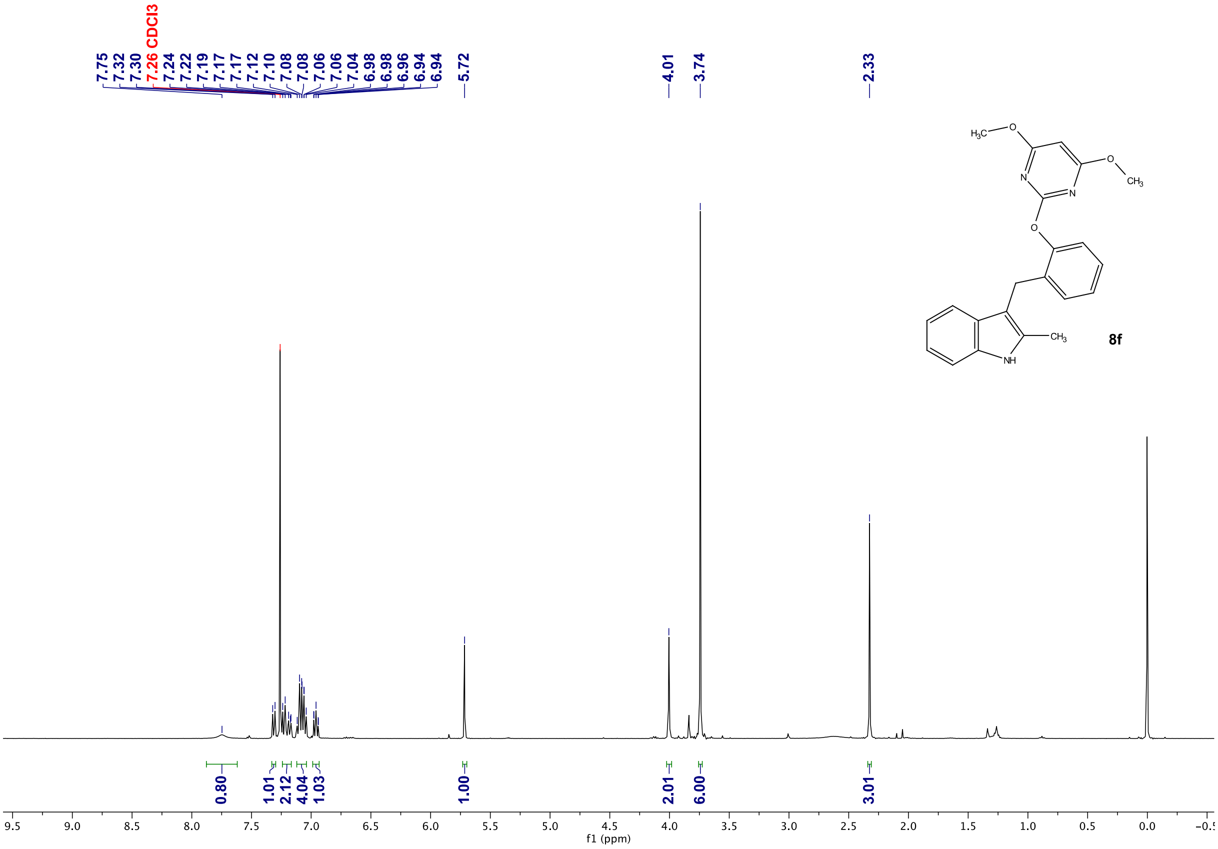

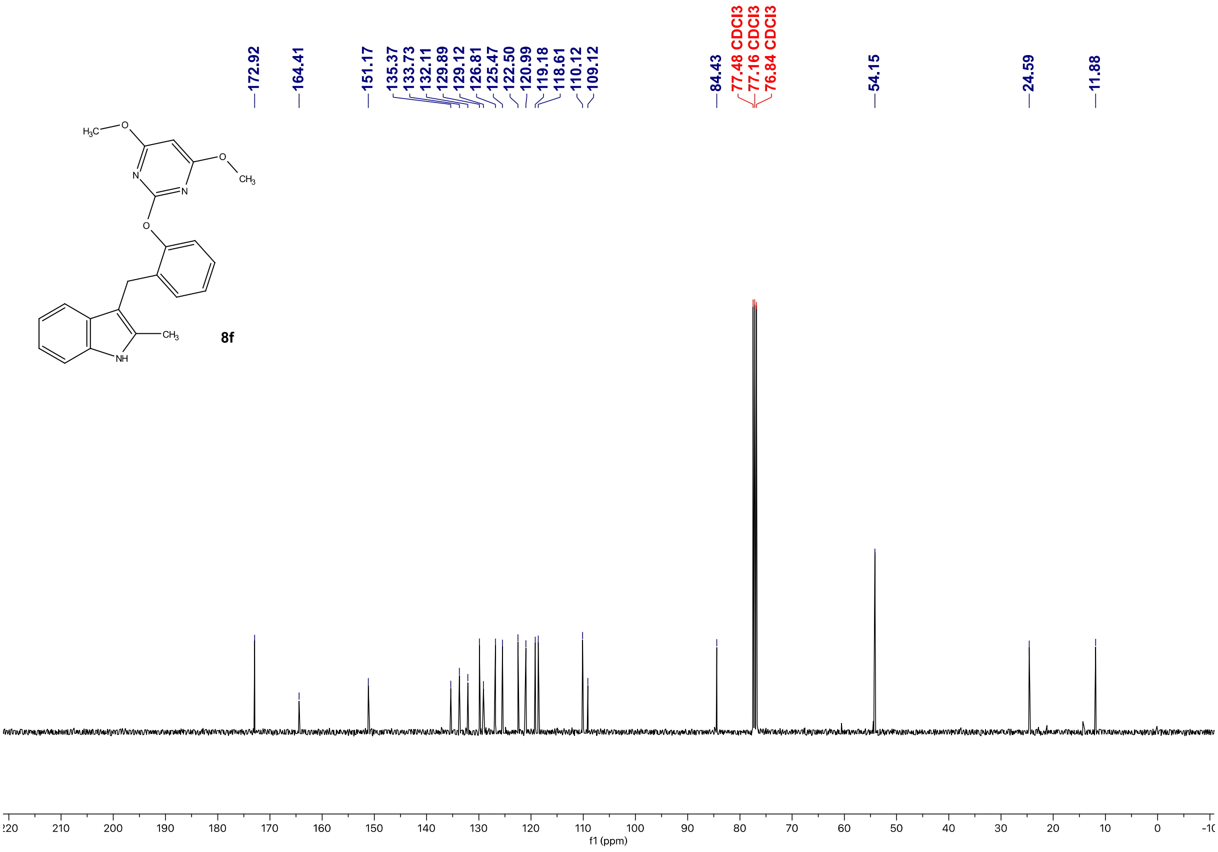

8g (400 MHz, $CDCl_3$)

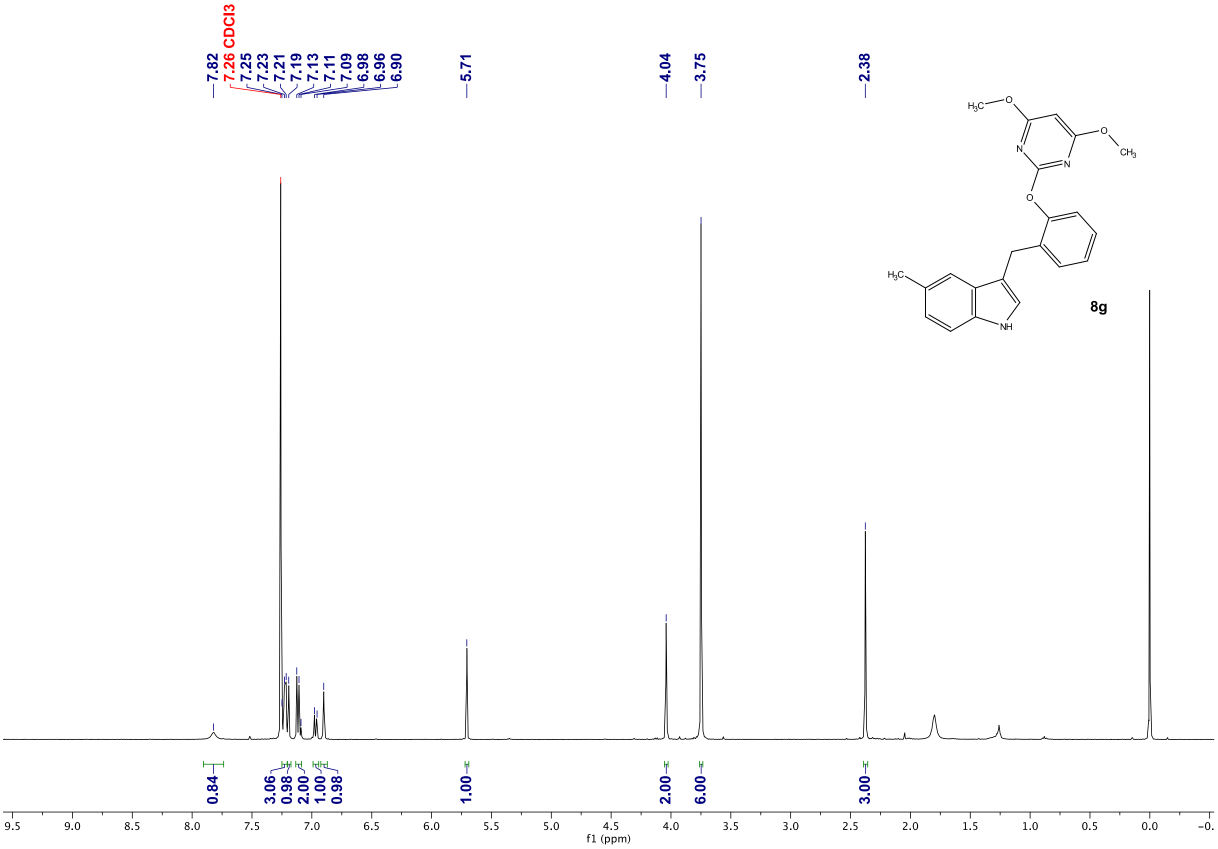

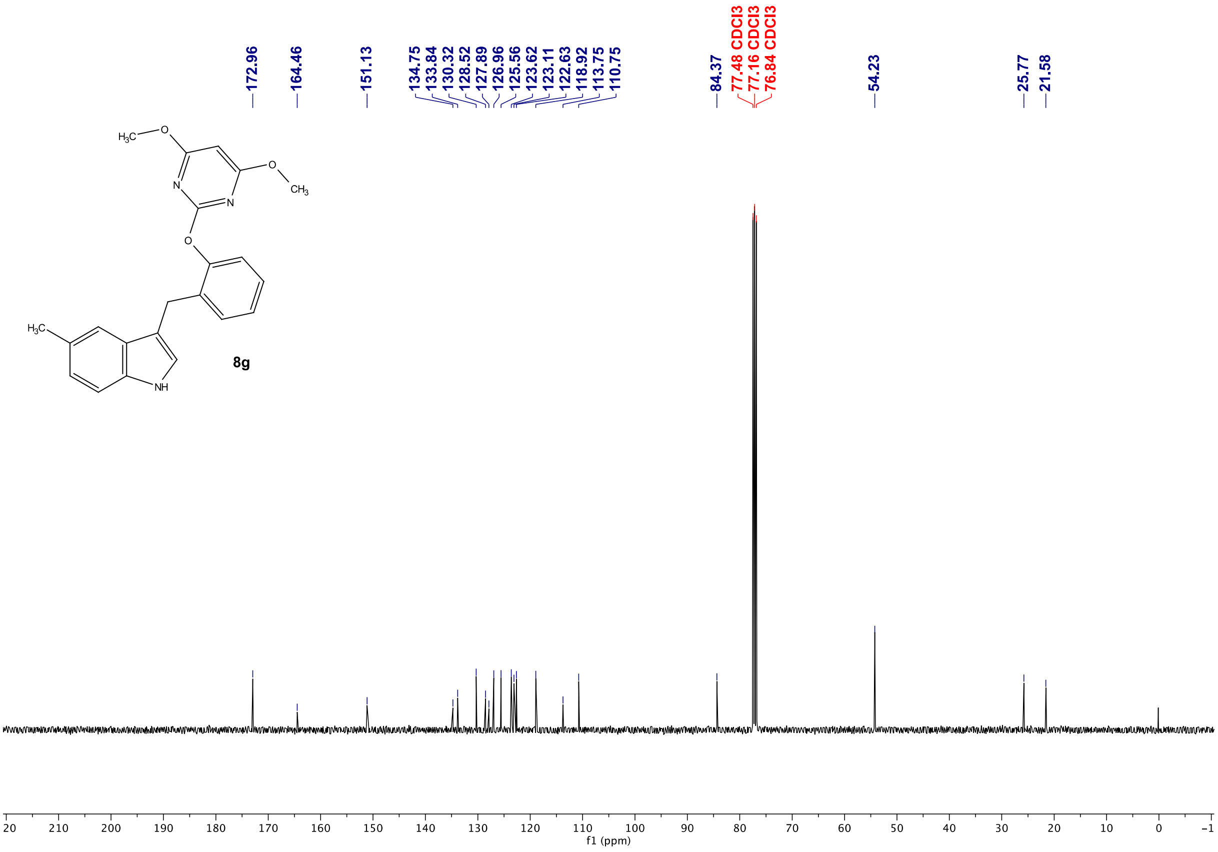

8h (400 MHz, $CDCl_3$)

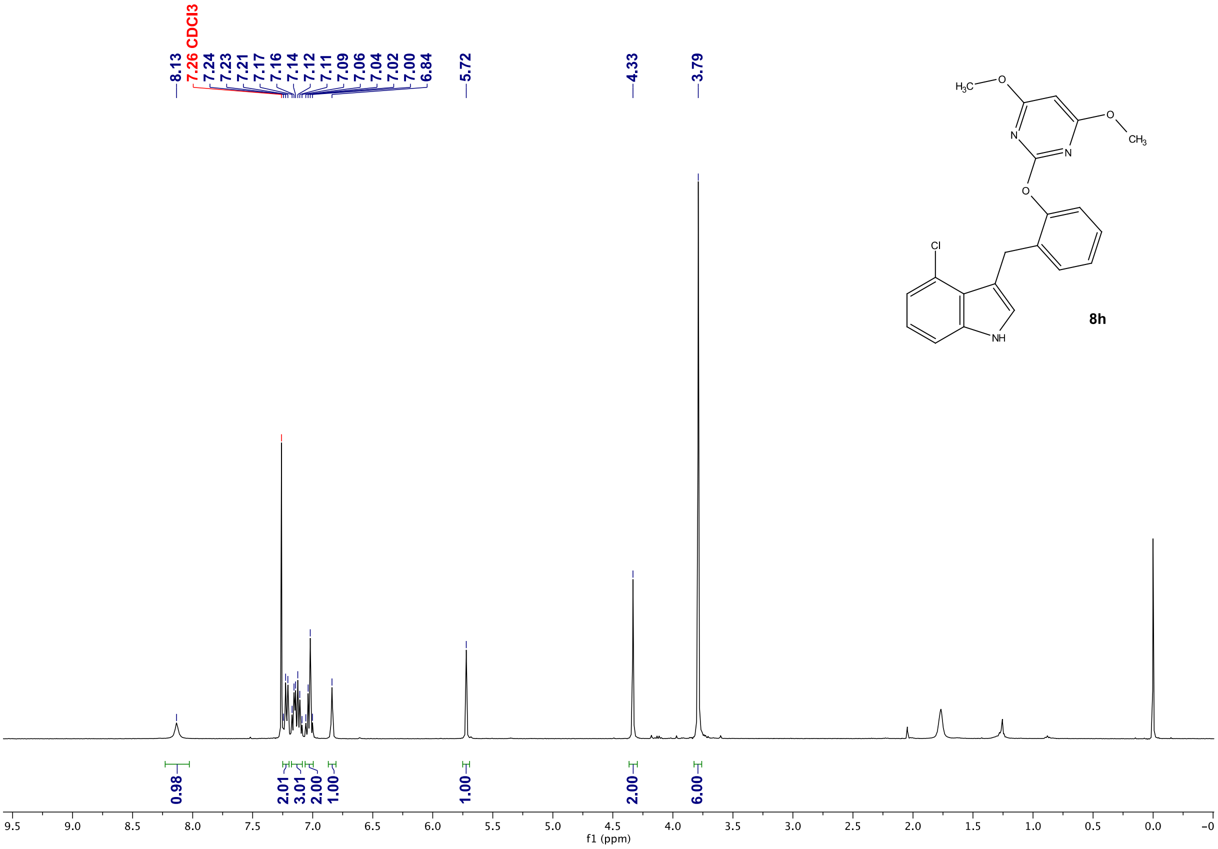

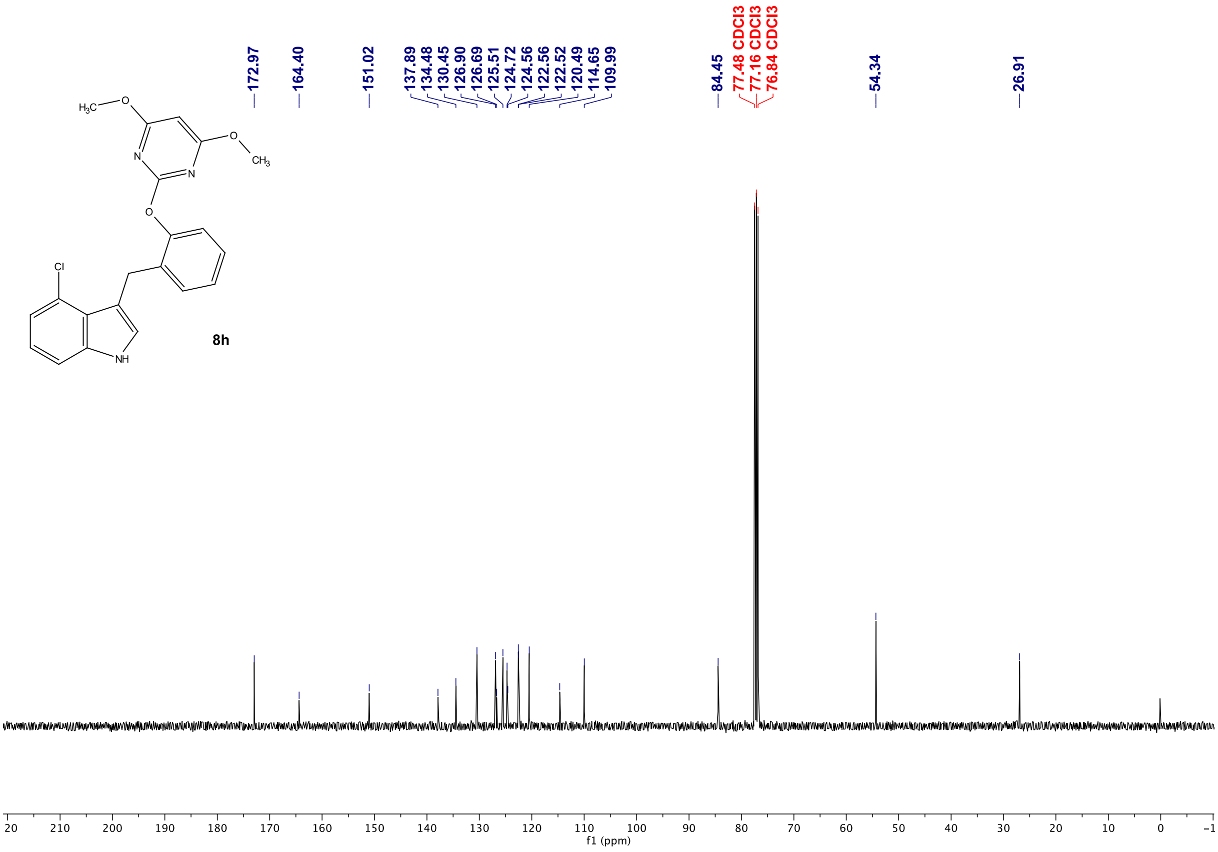

8i (400 MHz, $CDCl_3$)

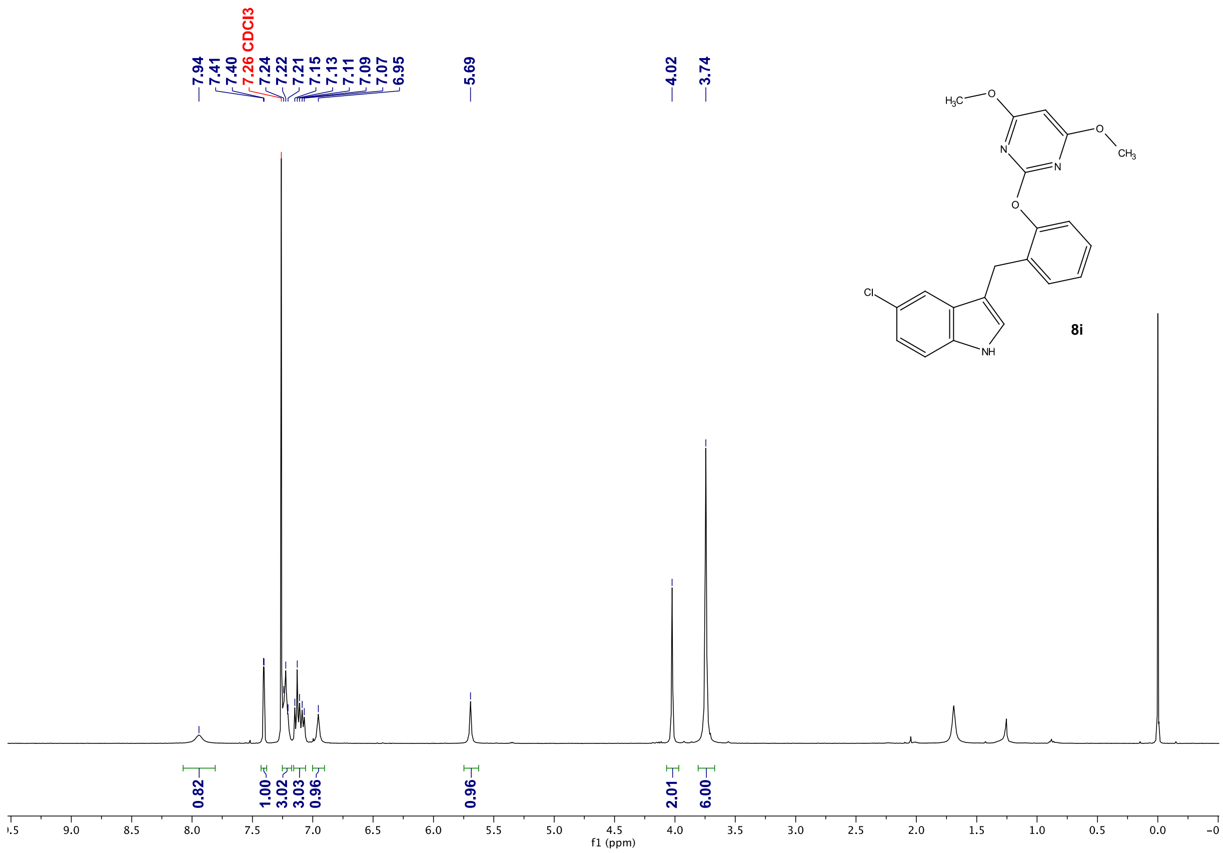

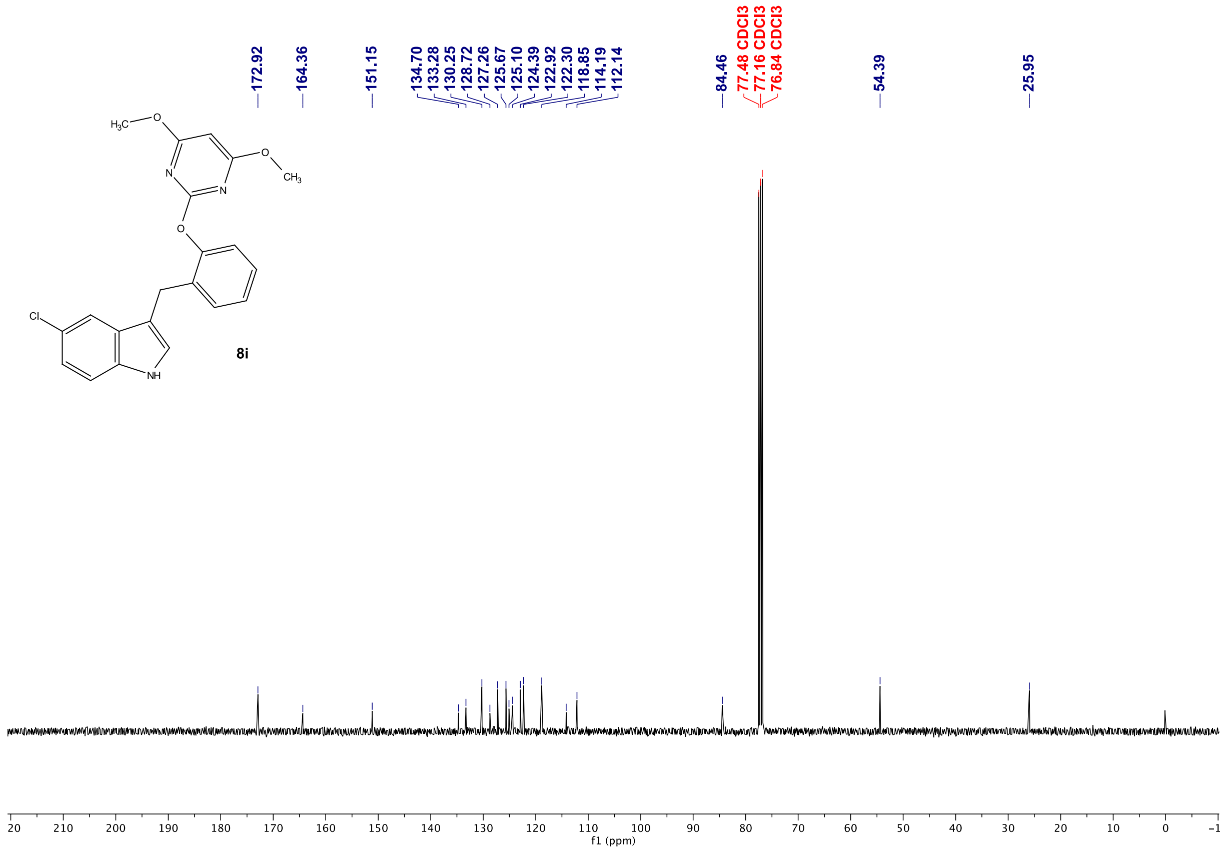

8j (400 MHz, DMSO-d_6)

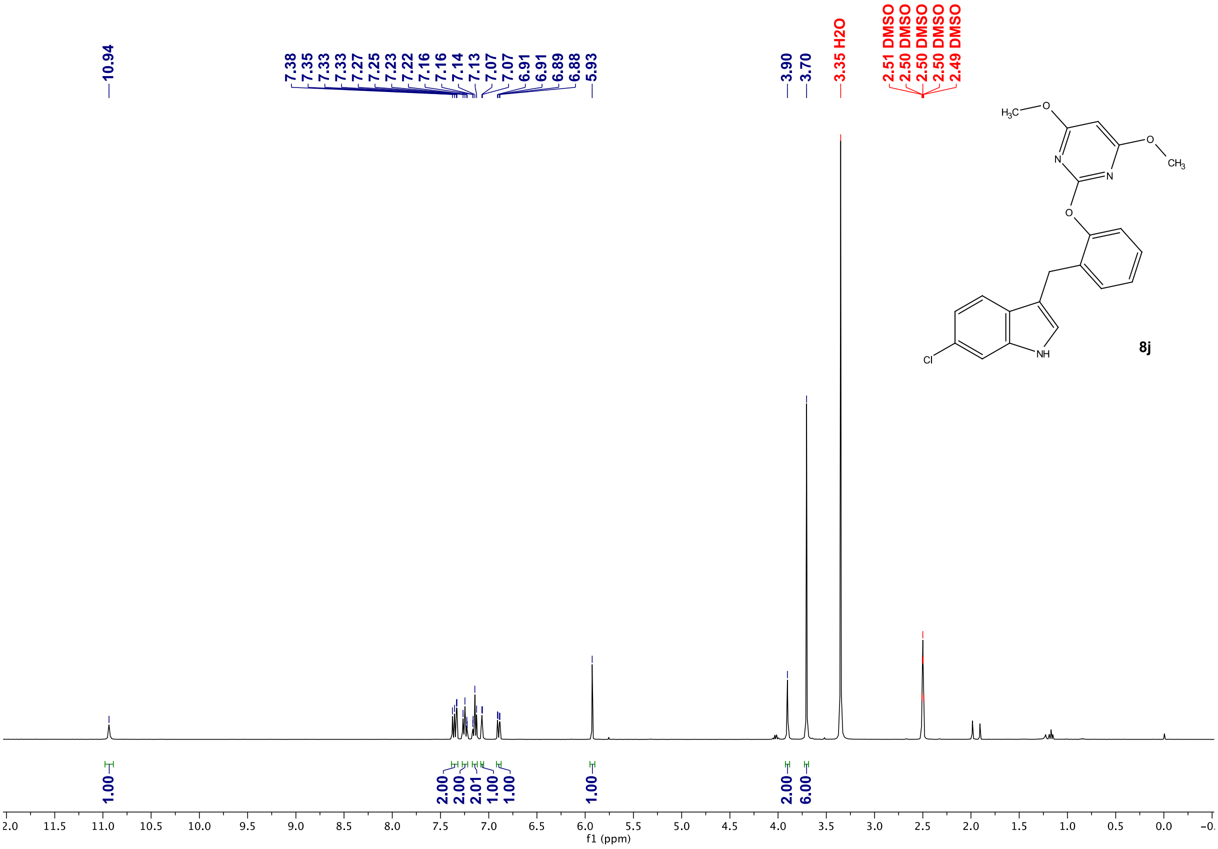

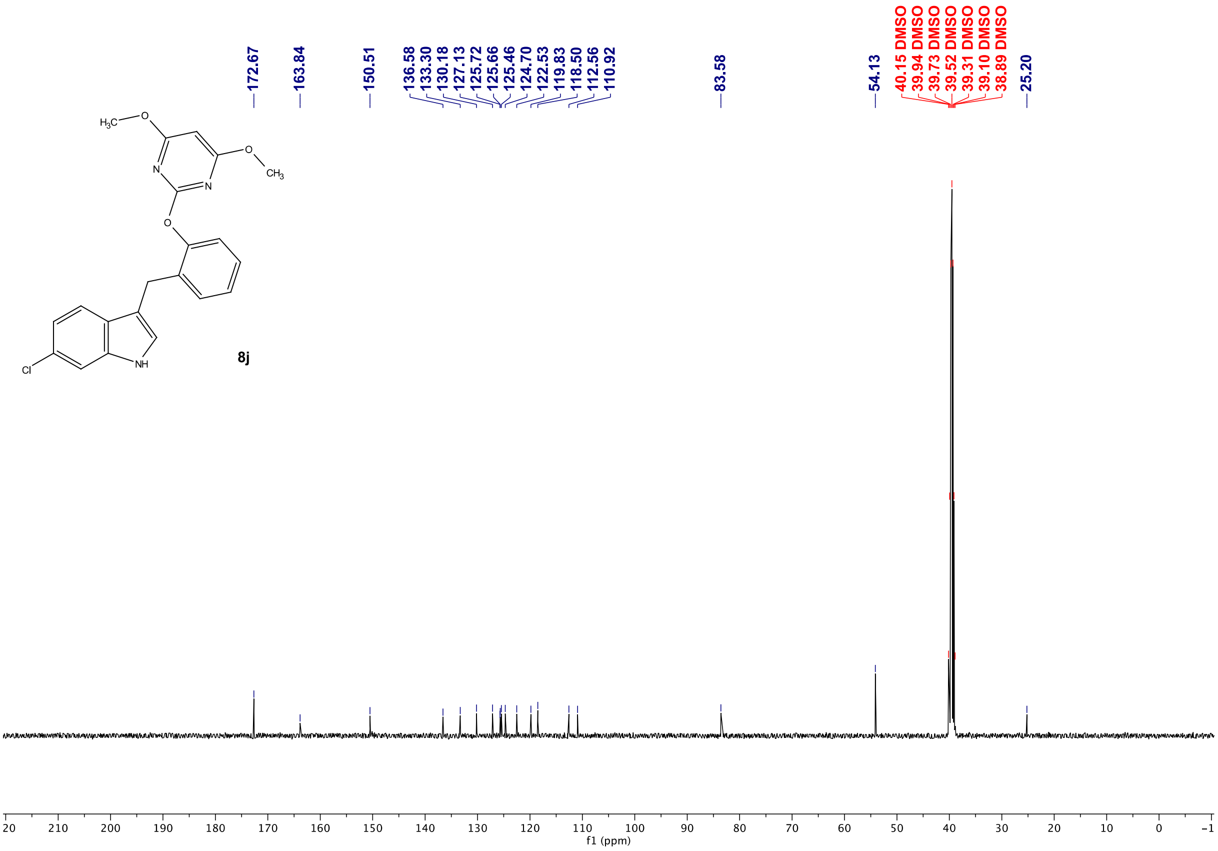

8k (400 MHz, $CDCl_3$)

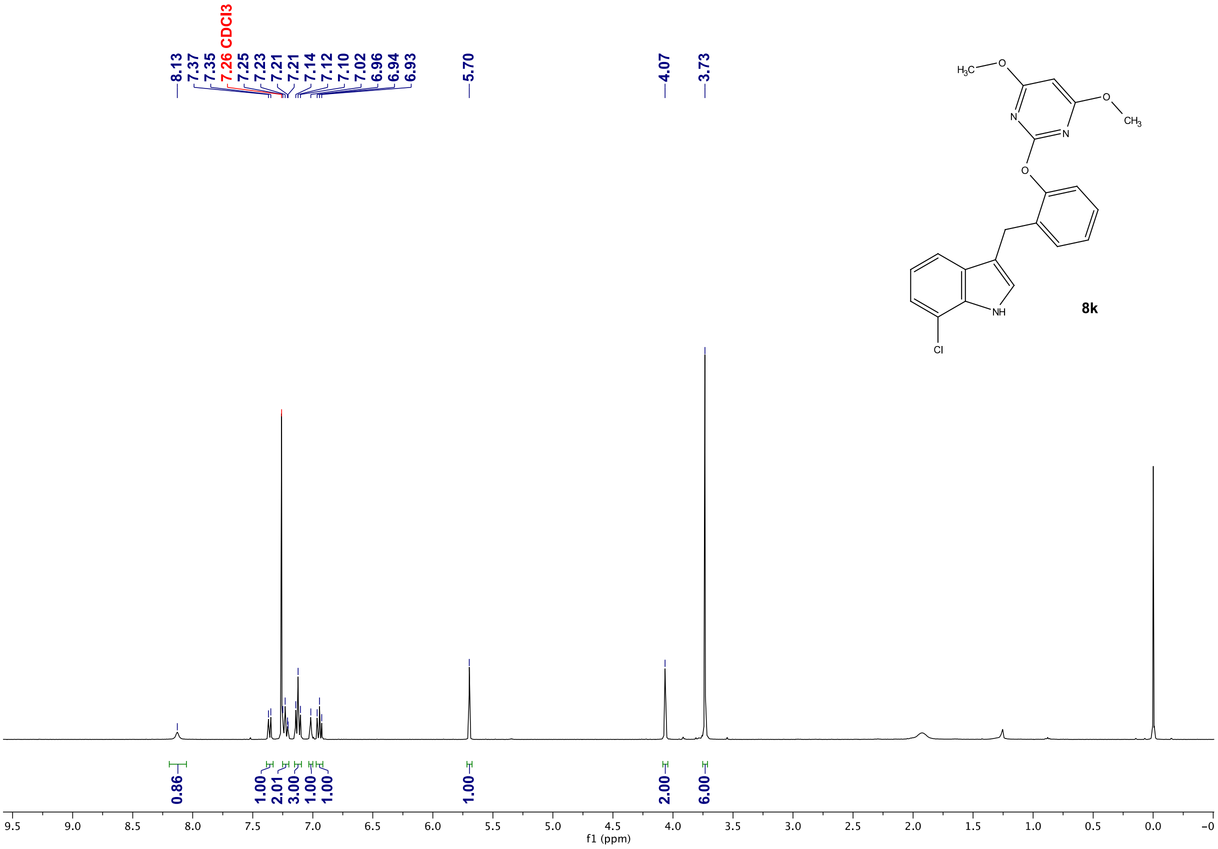

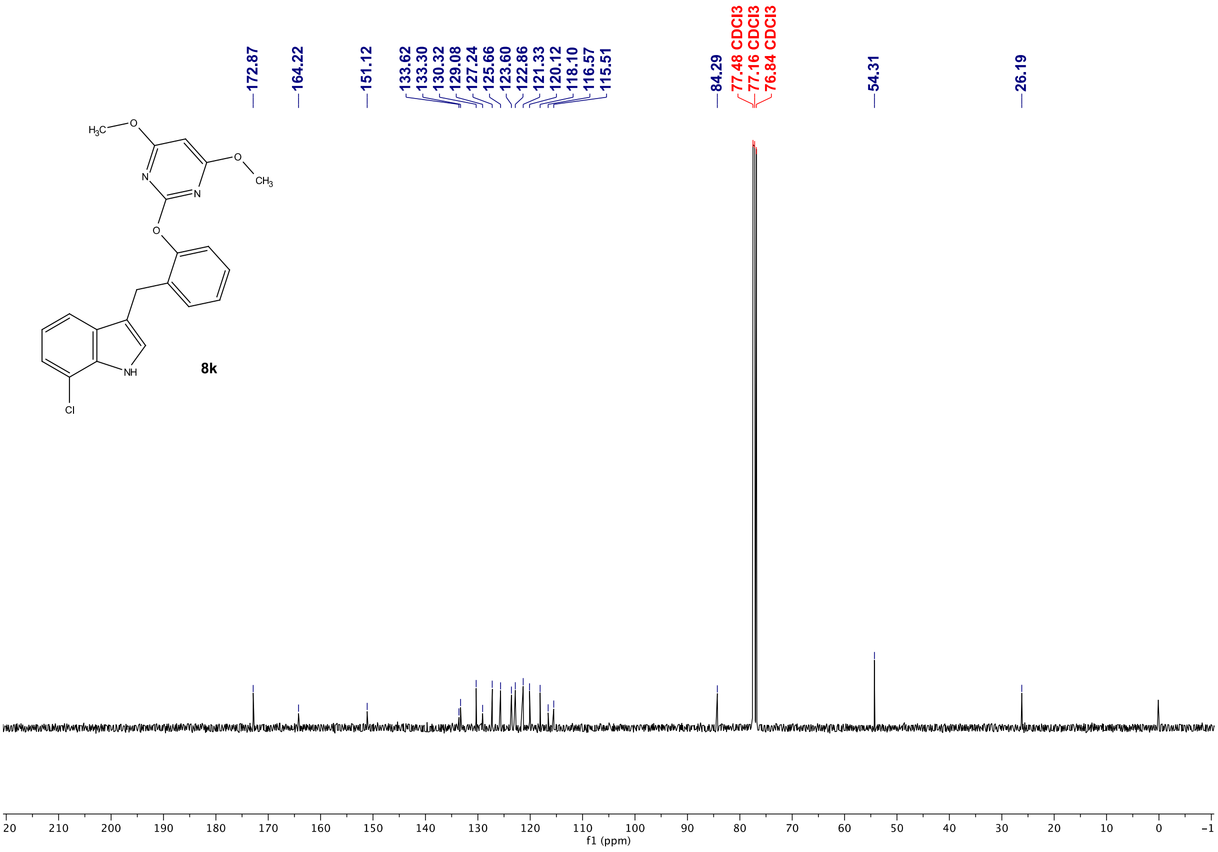

9g (400 MHz, $CDCl_3$)

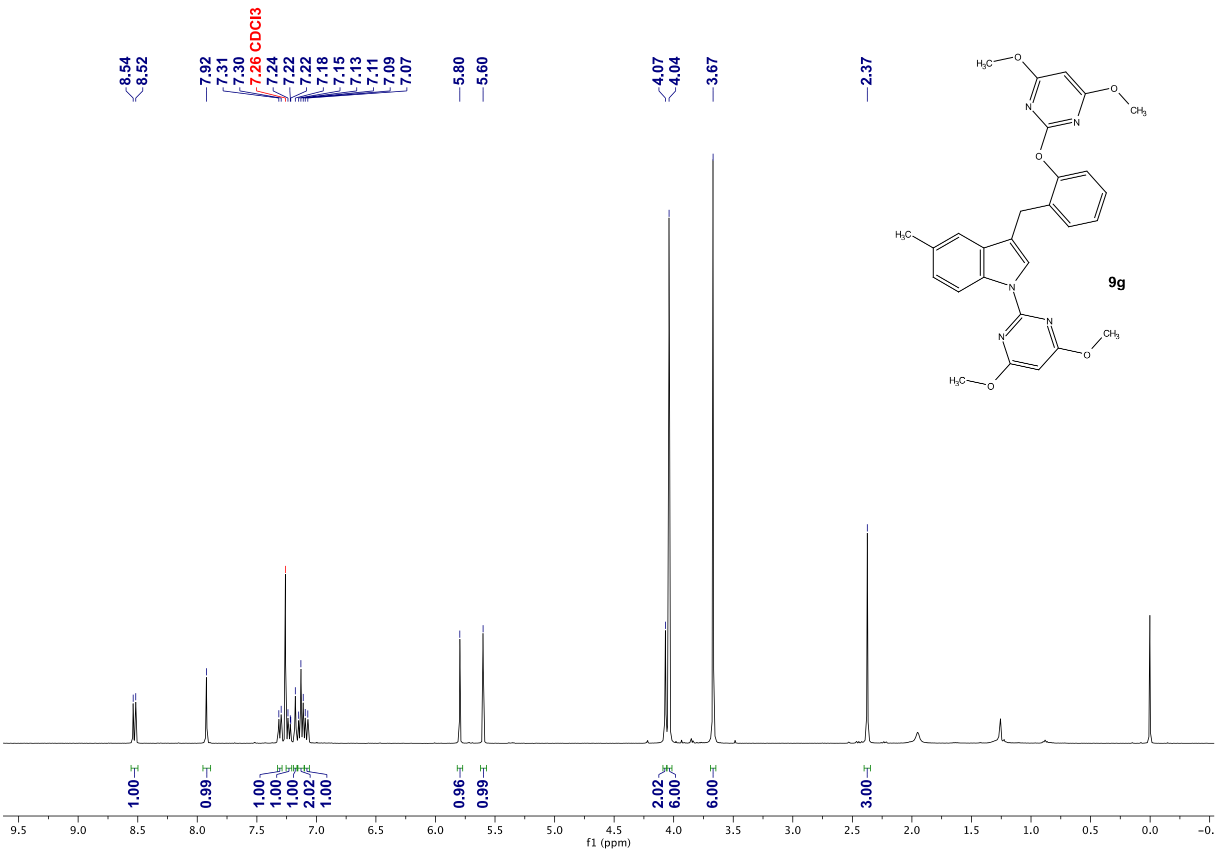

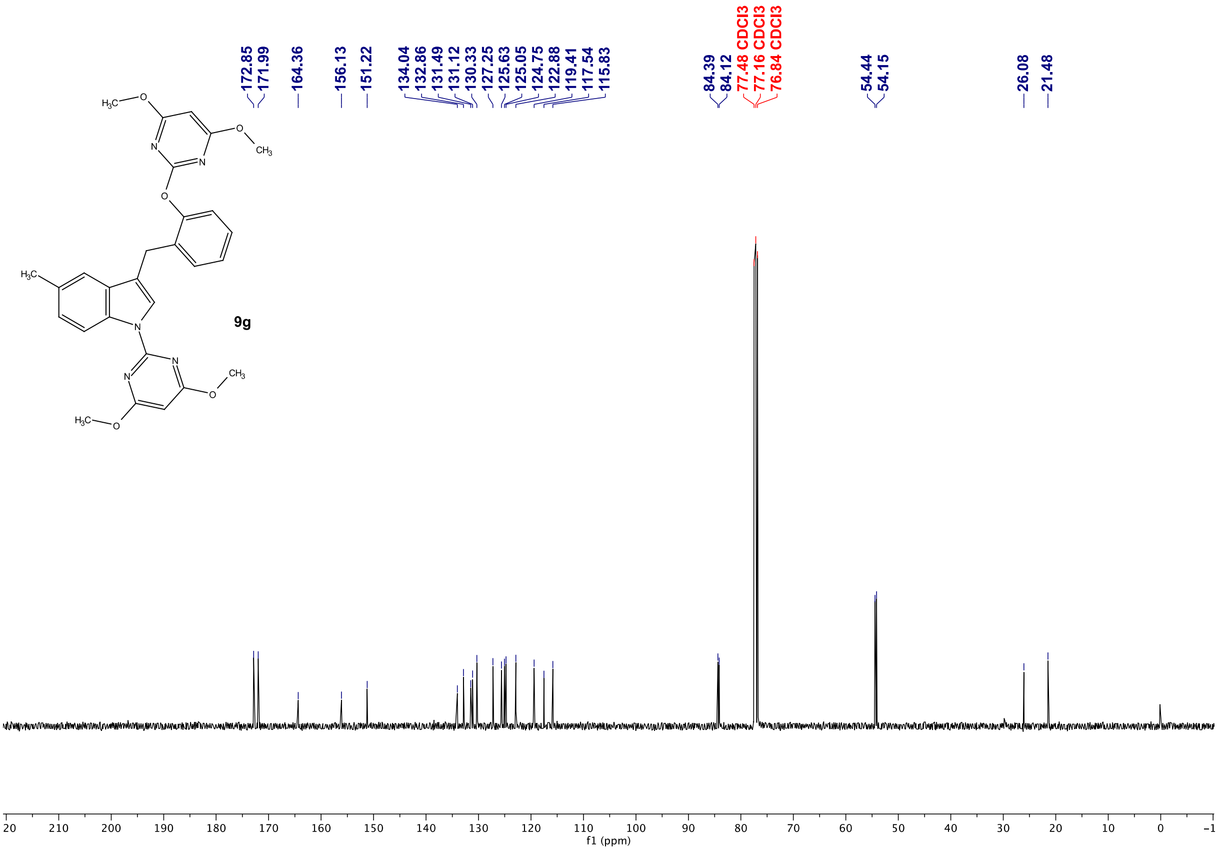

9h (400 MHz, $CDCl_3$)

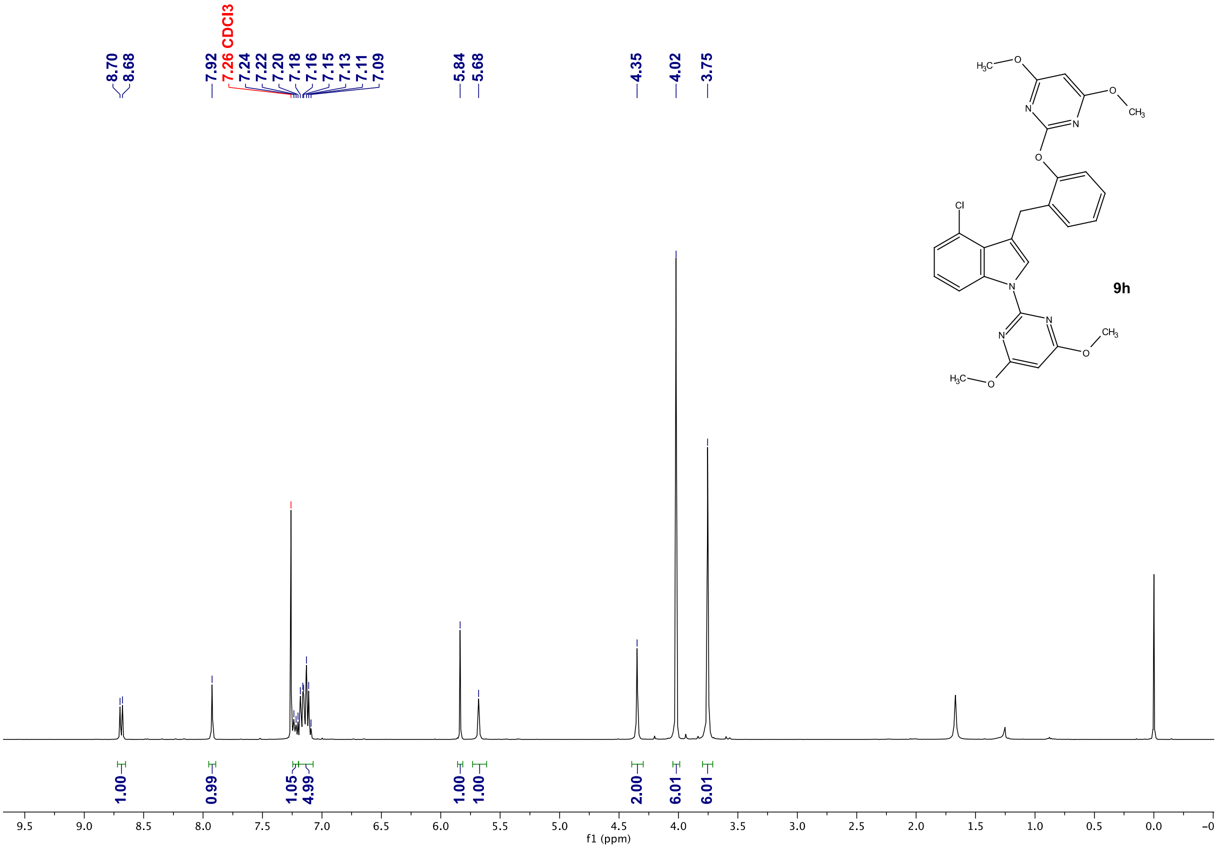

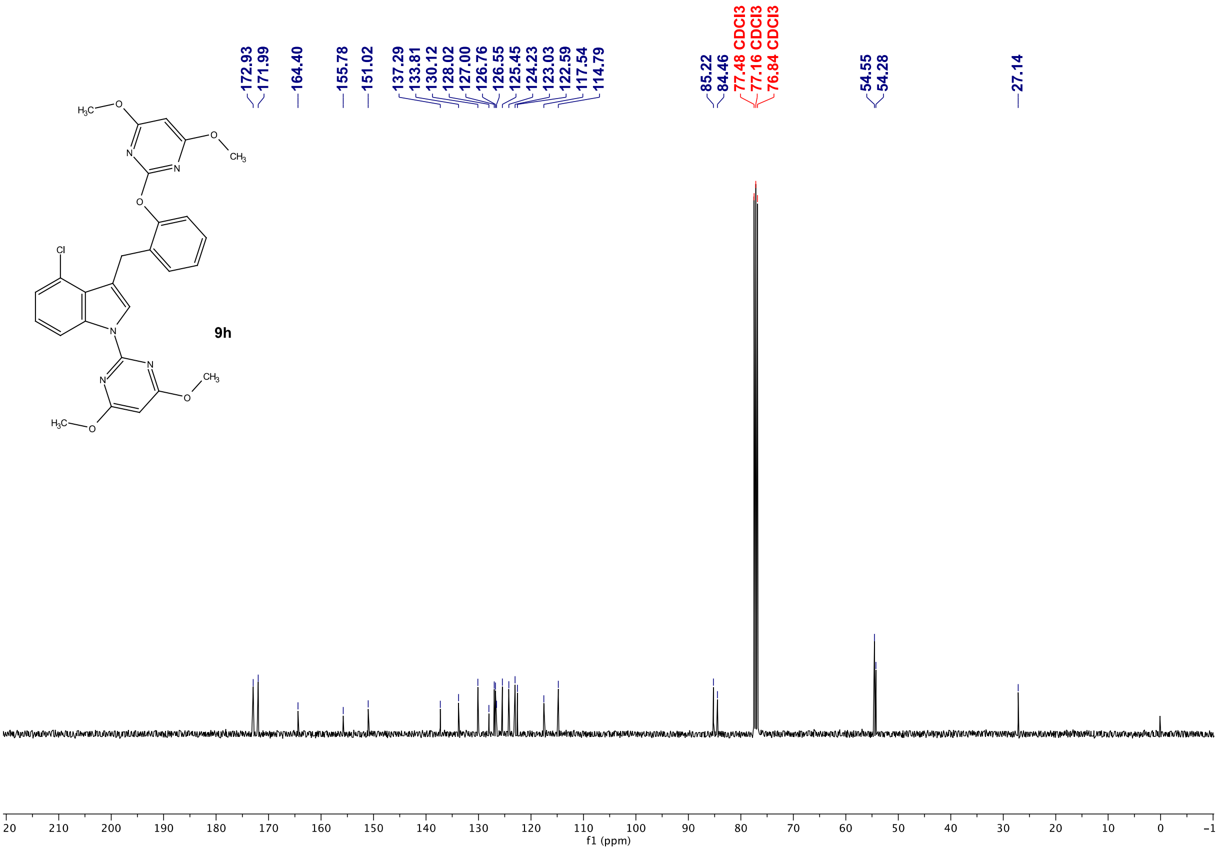

9i (400 MHz, $CDCl_3$)

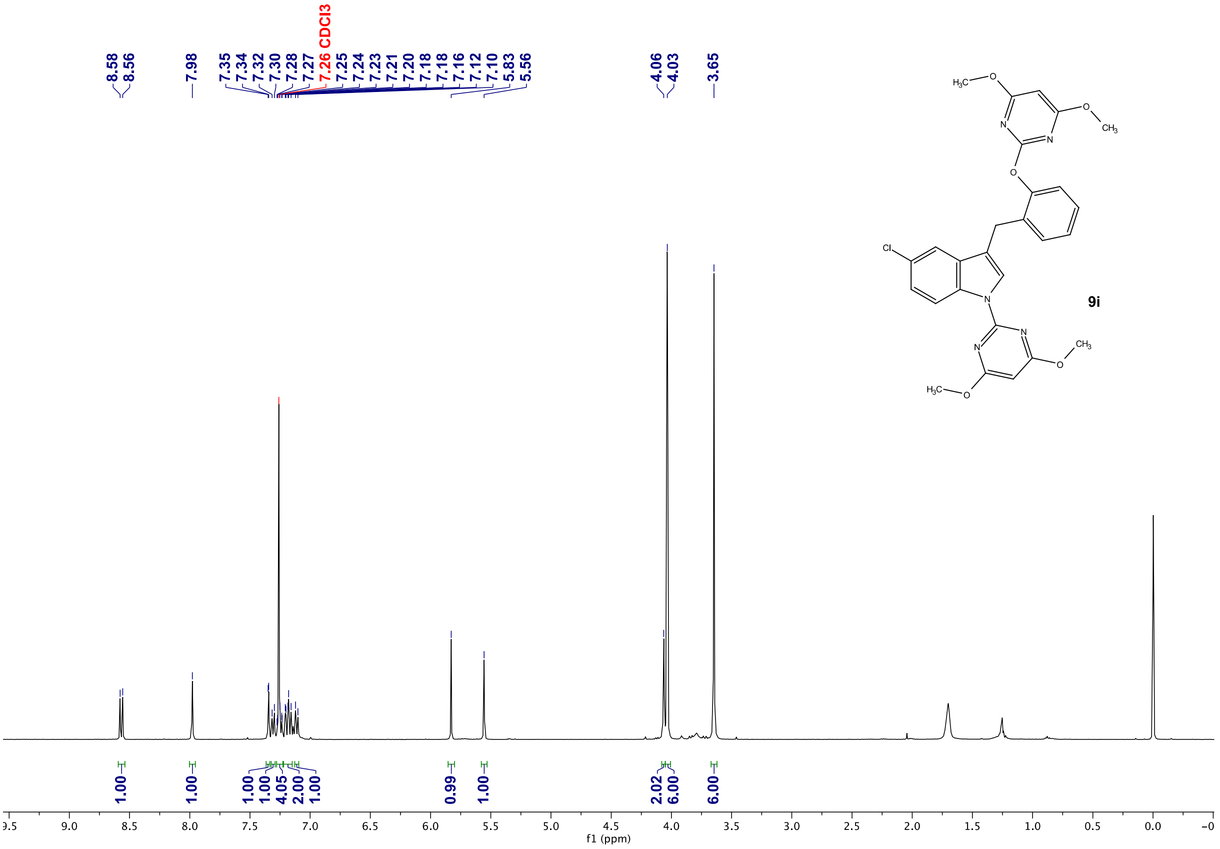

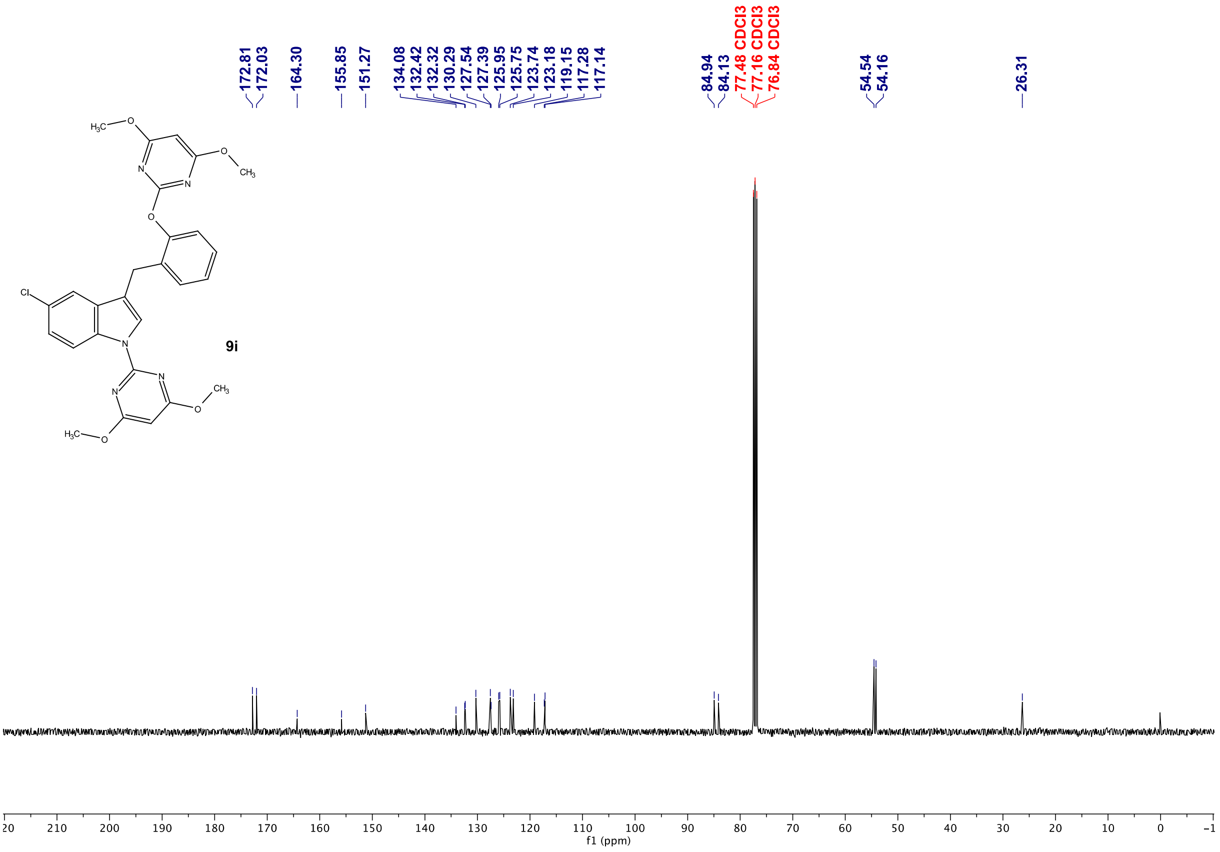

9j (400 MHz, $CDCl_3$)

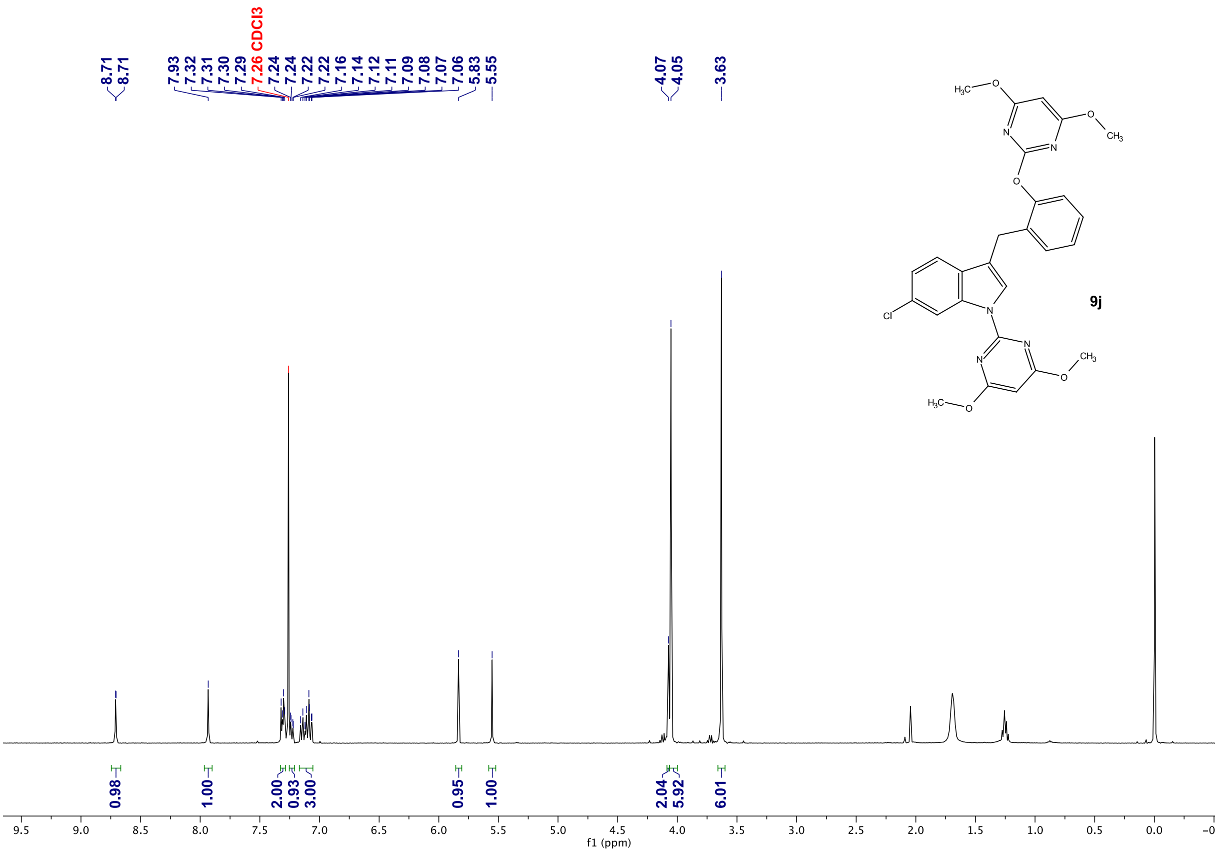

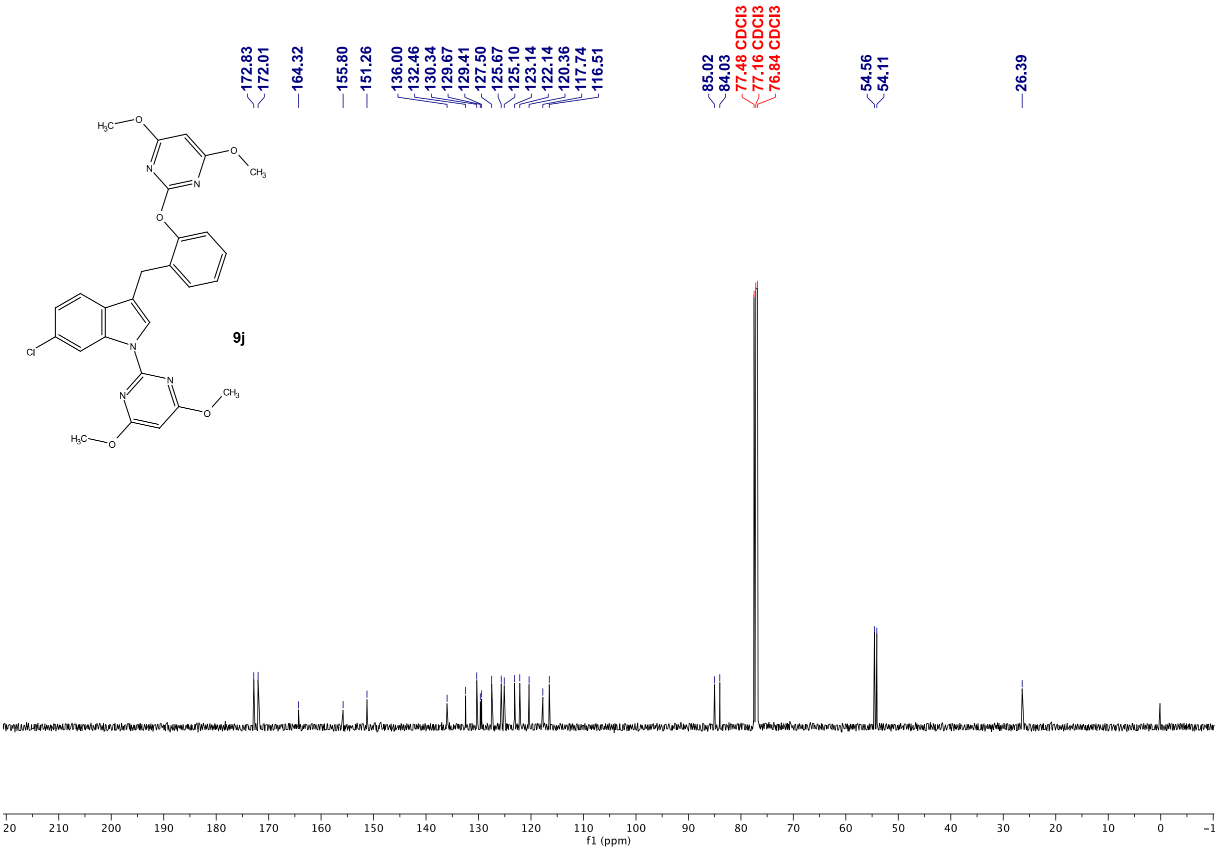

Supplement: RA-011-D1RA02663A-s001 [file RA-011-D1RA02663A-s001.pdf]
